# Macrophage DCLK1 promotes atherosclerosis via binding to IKKβ and inducing inflammatory responses

**DOI:** 10.15252/emmm.202217198

**Published:** 2023-03-10

**Authors:** Zhuqi Huang, Sirui Shen, Xue Han, Weixin Li, Wu Luo, Liming Lin, Mingjiang Xu, Yi Wang, Weijian Huang, Gaojun Wu, Guang Liang

**Affiliations:** ^1^ Department of Cardiology The First Affiliated Hospital of Wenzhou Medical University Wenzhou China; ^2^ Chemical Biology Research Center, School of Pharmaceutical Sciences Wenzhou Medical University Wenzhou China; ^3^ School of Pharmaceutical Sciences Hangzhou Medical College Wenzhou China

**Keywords:** atherosclerosis, DCLK1, IKKβ, inflammation, macrophage, Cardiovascular System, Vascular Biology & Angiogenesis

## Abstract

Atherosclerosis is a chronic inflammatory disease with high morbidity and mortality rates worldwide. Doublecortin‐like kinase 1 (DCLK1), a microtubule‐associated protein kinase, is involved in neurogenesis and human cancers. However, the role of DCLK1 in atherosclerosis remains undefined. In this study, we identified upregulated DCLK1 in macrophages in atherosclerotic lesions of ApoE^−/−^ mice fed an HFD and determined that macrophage‐specific DCLK1 deletion attenuates atherosclerosis by reducing inflammation in mice. Mechanistically, RNA sequencing analysis indicated that DCLK1 mediates oxLDL‐induced inflammation via NF‐κB signaling pathway in primary macrophages. Coimmunoprecipitation followed by LC–MS/MS analysis identified IKKβ as a binding protein of DCLK1. We confirmed that DCLK1 directly interacts with IKKβ and phosphorylates IKKβ at S177/181, thereby facilitating subsequent NF‐κB activation and inflammatory gene expression in macrophages. Finally, a pharmacological inhibitor of DCLK1 prevents atherosclerotic progression and inflammation both *in vitro* and *in vivo.* Our findings demonstrated that macrophage DCLK1 promotes inflammatory atherosclerosis by binding to IKKβ and activating IKKβ/NF‐κB. This study reports DCLK1 as a new IKKβ regulator in inflammation and a potential therapeutic target for inflammatory atherosclerosis.

## Graphical abstract

Macrophage DCLK1 promotes inflammatory atherosclerosis by directly binding to IKKβ and activating IKKβ/NF‐κB signal. DCLK1 is a new regulator of IKKβ and a potential therapeutic target for atherosclerosis.

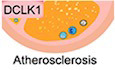

**The paper explained**

**Problem**

Atherosclerosis is a chronic inflammatory disease with high morbidity and mortality rates worldwide. Doublecortin‐like kinase 1 (DCLK1), a microtubule‐associated protein kinase, is involved in neurogenesis and human cancers. Recent studies suggest that DCLK1 may be involved in inflammatory colitis. However, the cellular pathways engaged by DCLK1 regulating inflammation, and its potential role in atherosclerosis remain undefined.

**Results**

We determined upregulated DCLK1 in macrophages in atherosclerotic lesions of HFD‐fed ApoE^−/−^ mice. Macrophage‐specific DCLK1 deletion in ApoE^−/−^ mice attenuates HFD‐induced atherosclerosis by reducing inflammation. Mechanistically, DCLK1 directly interacts with IKKβ and phosphorylates IKKβ at S177/181, resulting in subsequent NF‐κB activation and inflammatory gene expression in macrophages. Pharmacological inhibitor of DCLK1 also prevents inflammatory atherosclerosis in mice.

**Impact**

This study extends our understanding of the role of DCLK1 as a new IKKβ/NF‐κB activator in inflammatory regulation and indicates DCLK1 as a potential therapeutic target for inflammatory atherosclerosis.

## Introduction

Atherosclerosis is a major cardiovascular disease with high morbidity and mortality rates worldwide (Herrington *et al*, 2016). One decisive event in atherosclerotic progression is the chronic inflammatory response (Hansson, 2005), which is characterized by the infiltration of inflammatory cells (macrophages, neutrophils, and monocytes) and secretion of proinflammatory factors (Rocha & Libby, 2009). The pivotal process is initiated when the vascular endothelium is activated by the accumulation of low‐density lipoproteins (LDL). Consequently, macrophages are recruited to atherosclerotic lesions and secrete abundant proinflammatory factors, which predominates in both atherosclerotic initiation and evolution (Libby, 2002; Hansson *et al*, 2006). Therefore, identifying innovative regulatory molecules of inflammatory atherosclerosis and elucidating their mechanisms of action have great scientific implications and clinical value and may provide potential targets for treating atherosclerosis.

Doublecortin‐like kinase 1 (DCLK1) is a microtubule‐associated serine/threonine kinase initially recognized in the nervous system where it manages microtubule polymerization and facilitates neuronal migration (Nevi *et al*, 2021). Outside of the nervous system, DCLK1 was proposed to mark quiescent gastrointestinal and pancreatic stem cells (Bailey *et al*, 2014; Westphalen *et al*, 2016). Since then, a number of studies have shown a range of DCLK1 activities in tumorigenesis (Sureban *et al*, 2014; Chandrakesan *et al*, 2017). Increased levels of DCLK1 have also been reported in various human cancers (Nevi *et al*, 2021; Kim *et al*, 2022). Recently, DCLK1 has been reported to play an important role in inflammatory diseases. Kim *et al* (2022) reported that DCLK1 promotes colorectal cancer by generating an inflammatory tumor microenvironment. Roy *et al* (2021) determined that the Notch‐DCLK1 axis is integral to colitis progression. Meanwhile, another study confirmed that DCLK1 mediates the response to inflammatory factors, facilitates inflammation‐driven epithelial restitution, and alleviates chronic colitis (Yi *et al*, 2019). These studies suggest that DCLK1 may regulate inflammation. However, the cellular pathways that may be engaged by DCLK1 as well as its substrates in inflammation remain undefined.

The role of DCLK1 in atherosclerosis and cardiovascular diseases has not been studied yet. The background check on DCLK1 has inspired us to examine the possible involvement of DCLK1 in the aortic tissues of atherosclerotic mice, which were originally used in other projects of our group. Interestingly, we found that DCLK1 expression is upregulated in macrophages in atherosclerotic lesions of mice. Then, we investigated the role of DCLK1 in high‐fat diet‐induced atherosclerosis. We showed that macrophage‐specific deletion or pharmacological inhibition of DCLK1 attenuates atherosclerotic progression by inhibiting NF‐κB‐mediated inflammation both *in vivo* and *in vitro*. Mechanistically, DCLK1 directly interacts with IKKβ to promote IKKβ phosphorylation at S177/181 and subsequent NF‐κB activation. Collectively, we have identified DCLK1 as a novel inflammatory regulator in atherosclerosis.

## Results

### DCLK1 is upregulated in macrophages of mouse atherosclerotic lesion

**Figure 1:**
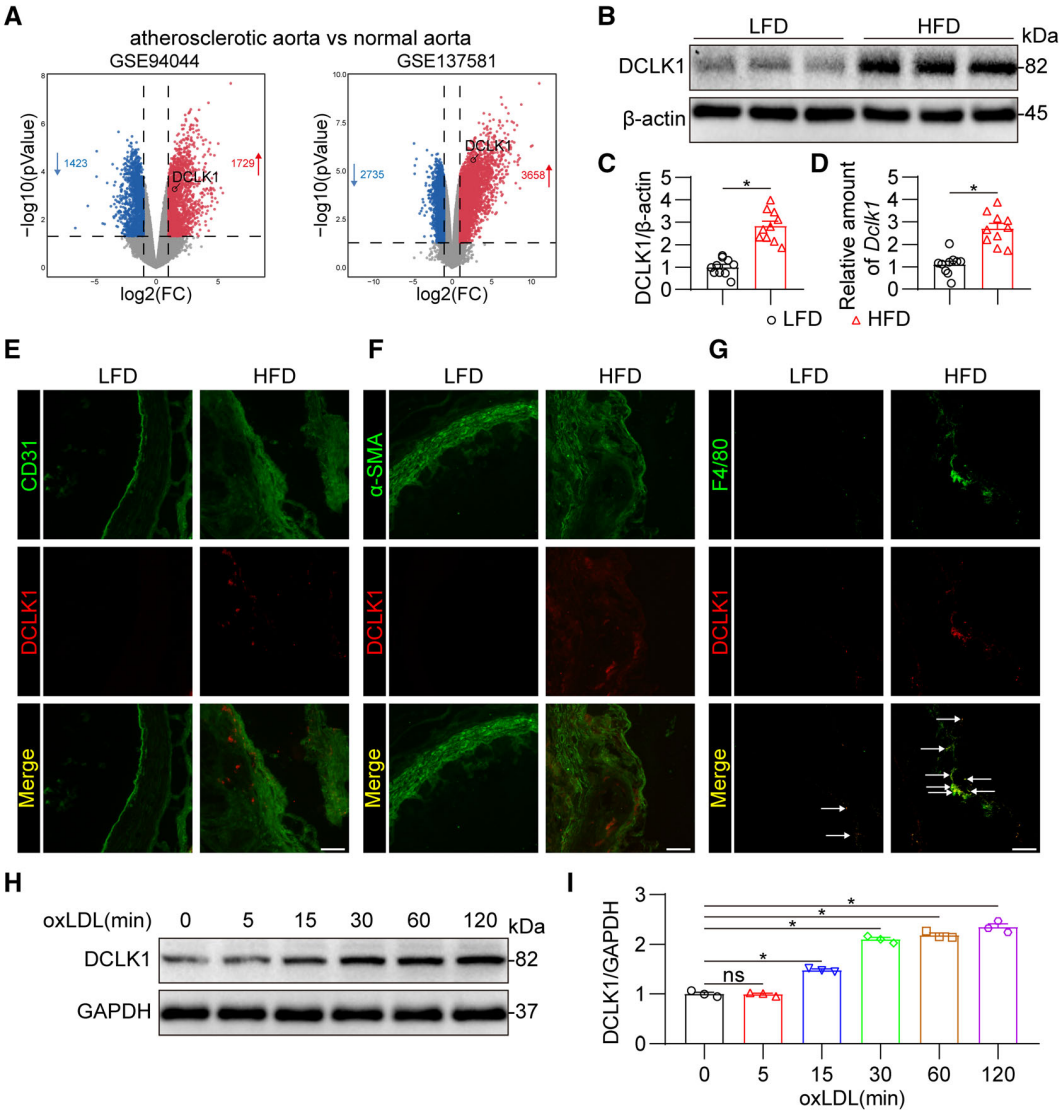
DCLK1 is up‐regulated in macrophages in atherosclerotic lesion AVolcano plot analysis of DEGs up‐regulated (red) or down‐regulated (blue) in atherosclerotic aortas compared to normal aortas from datasets GSE94044 and GSE137581. FC, fold change.B, CWestern blot analysis (B) and densitometric quantification (C) of DCLK1 in aortas of LFD and HFD‐fed ApoE^−/−^ mice. β‐actin was used as the loading control (*n* = 10 biological replicates).DmRNA levels of DCLK1 in aortas of LFD and HFD‐fed ApoE^−/−^ mice were determined by RT‐qPCR. The values were normalized to *Rn18s* (*n* = 10 biological replicates).ERepresentative immunofluorescence staining of CD31 (green) and DCLK1 (red) in aortic roots (scale bar = 25 μm).FRepresentative immunofluorescence staining of α‐SMA (green) and DCLK1 (red) in aortic roots (scale bar = 25 μm).GRepresentative immunofluorescence staining of F4/80 (green) and DCLK1 (red) in aortic roots (scale bar = 25 μm). White arrows indicate co‐location of F4/80 and DCLK1.H, ITime‐course of DCLK1 induction in response to oxLDL in mouse primary peritoneal macrophages (MPMs). MPMs were exposed to oxLDL (50 μg/mL) for indicated time. Western blot analysis (H) and densitometric quantification (I) of DCLK1 were shown. GAPDH was used as the loading control (*n* = 3 biological replicates). Data information: Data were shown as mean ± SEM; *P < 0.05; ns, not significant, two‐tailed unpaired Student's *t*‐test. Source data are available online for this figure.

To determine whether DCLK1 is involved in atherosclerosis, we first examined the levels of DCLK1 in experimental atherosclerotic mouse models using publicly available transcriptome data. As shown in Fig 1A, DCLK1 was upregulated in atherosclerotic aortas of mice compared to that in normal mouse aortas. We built atherosclerotic mice using ApoE^−/−^ mice fed with a high‐fat diet (HFD) for 4 months. Consistently, our data validated that DCLK1 was upregulated in the aortas of HFD‐fed ApoE^−/−^ mice at both protein (Fig 1B and C) and mRNA (Fig 1D) levels. We then examined the cellular distribution of DCLK1 in aortas. Immunofluorescence staining showed that DCLK1 colocalized with F4/80, a macrophage marker, rather than with CD31, an endothelial cell marker, and α‐SMA, a smooth muscle cell marker in mouse aortas (Fig 1E–G). Figure 1E–G also showed that the expression of DCLK1 was markedly increased in macrophages in atherosclerotic plaques of HFD‐fed ApoE^−/−^ mice. We then identified that DCLK1 expression indeed increased in MPMs challenged with oxLDL in a time‐dependent manner (Fig 1H and I). These data suggest that DCLK1 is upregulated in macrophages of atherosclerotic lesions and may be involved in the atherosclerotic progression.

### Macrophage‐specific DCLK1 deletion reduces atherosclerotic plaques in HFD‐fed ApoE ^−/−^ mice

**Figure 2:**
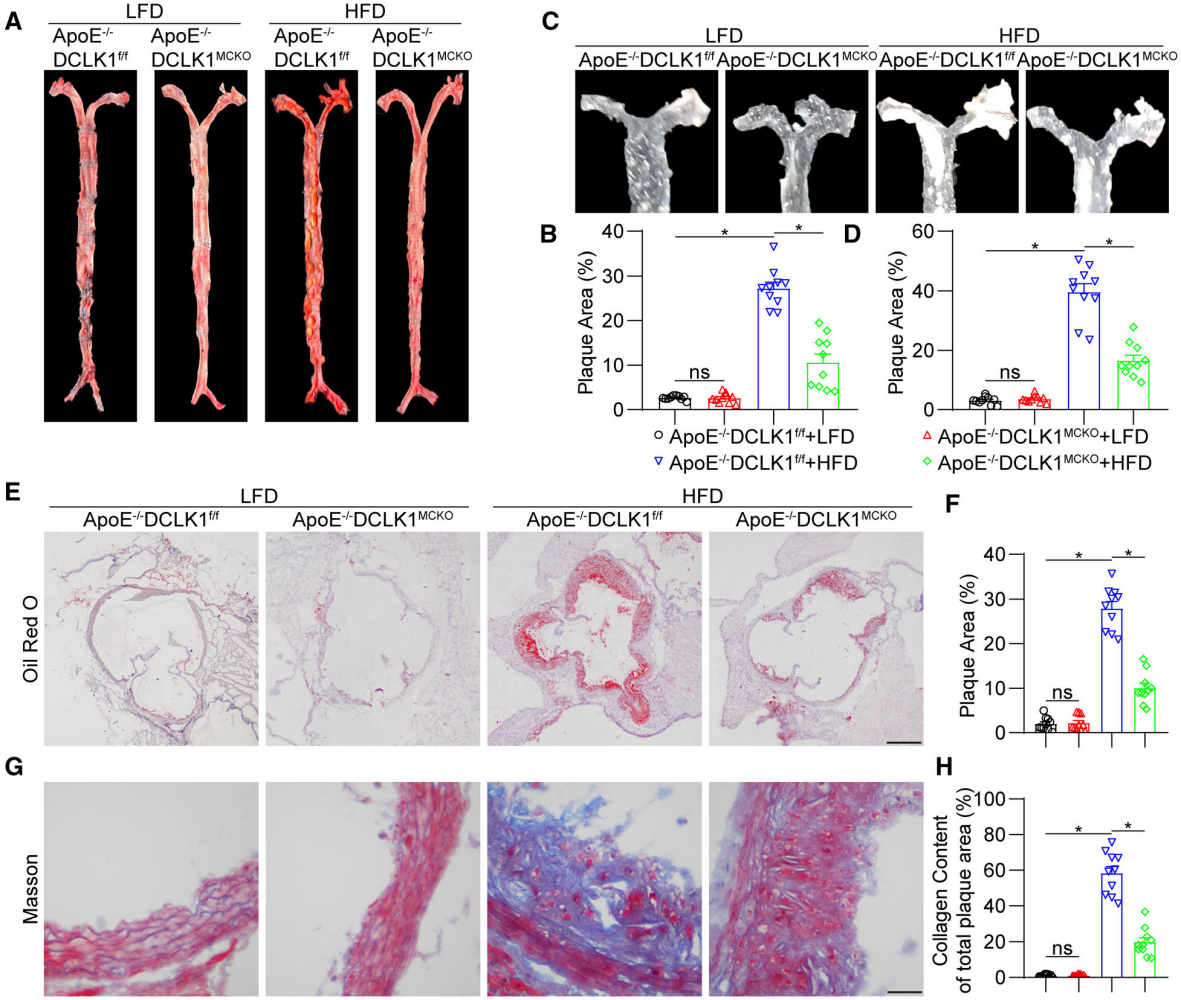
Macrophage‐specific DCLK1 deletion reduces atherosclerotic plaques in HFD‐fed ApoE^−/−^ mice The animal experiment using ApoE^−/−^DCLK1^f/f^ and ApoE^−/−^DCLK1^MCKO^ mice fed with or without HFD was described in the Materials and Methods section.
A, BRepresentative en face Oil Red O staining (A) and quantification (B) of Oil Red O‐positive plaque lesion area in aortas. Plaque area was defined as percentage of total surface area of the aorta (*n* = 10 biological replicates).C, DRepresentative images (C) and quantification (D) of plaque lesion area in aortic arches (*n* = 10 biological replicates). The plaque area was quantified by the proportion of plaque area to aortic arches area.E, FRepresentative images of Oil Red O staining (E) and quantification (F) of atherosclerotic lesion in aortic roots (scale bar = 250 μm, *n* = 10 biological replicates). Plaque area was quantified by the proportion of plaque area to aortic root area.G, HRepresentative images of Masson's Trichrome staining (G) and quantification (H) for collagen deposition in aortic roots (scale bar = 25 μm, *n* = 10 biological replicates). Data information: Data were shown as mean ± SEM; *P < 0.05; ns, not significant, two‐tailed unpaired Student's *t*‐test. Source data are available online for this figure.

To investigate the role of macrophage DCLK1 in atherosclerosis, macrophage‐specific DCLK1 knockout mice with an ApoE knockout background (ApoE^−/−^DCLK1^MCKO^) and the control DCLK1‐flox mice with an ApoE knockout background (ApoE^−/−^DCLK1^f/f^) were generated and used (Appendix Fig S1A and B). The DCLK1 deletion in macrophages was confirmed by Western blot analysis (Appendix Fig S1C). Feeding mice with a HFD for 16 weeks significantly increased the body weight and induced hyperlipidemia in ApoE^−/−^DCLK1^f/f^ mice compared to the low‐fat diet (LFD) group, while macrophage‐specific DCLK1 deletion did not affect the increased body weight and serum lipid profile in HFD‐fed ApoE^−/−^ mice (Appendix Fig S2A–D). Interestingly, HFD feeding resulted in atherosclerotic plaques in the aortas of ApoE^−/−^DCLK1^f/f^ mice, while macrophage‐specific DCLK1 deletion significantly reduced plaque size in the aortas of HFD‐fed ApoE^−/−^DCLK1^MCKO^ mice (Fig 2A–D). Similarly, Oil Red O staining of aortic roots showed that DCLK1 deletion attenuated atherosclerotic lesions in HFD‐fed ApoE^−/−^ mice (Fig 2E and F). Masson's trichome staining also revealed reduced collagen deposition in atherosclerotic plaques in the ApoE^−/−^DCLK1^MCKO^ mice with HFD compared to the ApoE^−/−^DCLK1^f/f^ + HFD group (Fig 2G and H). Collectively, these results indicate that macrophage‐specific DCLK1 deletion alleviates HFD‐induced atherosclerotic development without affecting serum lipid profile.

### Macrophage‐specific DCLK1 deletion suppresses inflammatory cell infiltration and alleviates inflammatory response in the aorta

**Figure 3:**
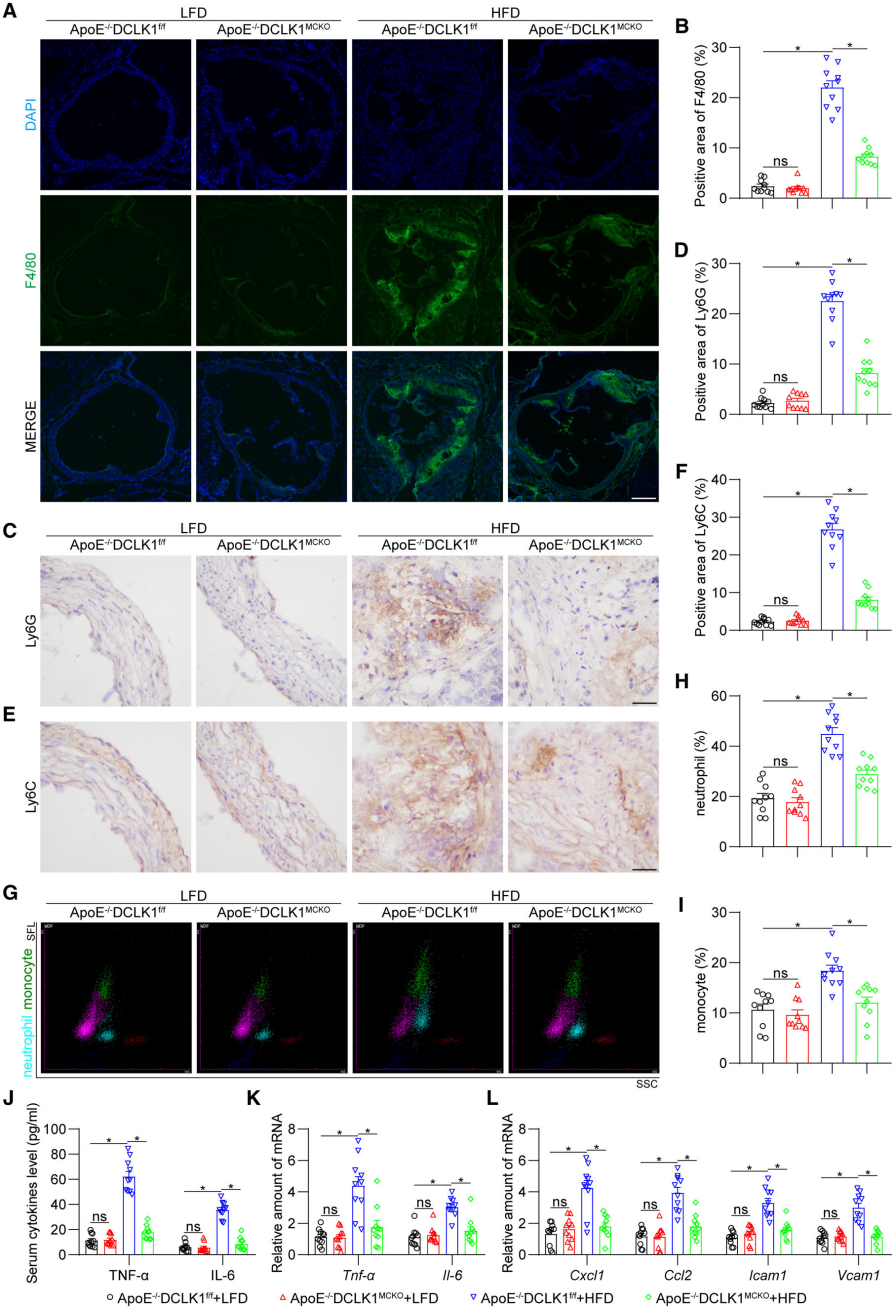
Macrophage‐specific DCLK1 deletion alleviates aortic inflammation and inflammatory cell infiltration in atherosclerotic lesions The animal experiment using ApoE^−/−^DCLK1^f/f^ and ApoE^−/−^DCLK1^MCKO^ mice fed with or without HFD was described in the Materials and Methods section.
A, BRepresentative immunofluorescence staining images (A) and quantification (B) of F4/80 (green) in aortic roots. Tissues were counterstained with DAPI (blue). F4/80 area was quantified by the proportion of F4/80‐positive area to aortic root area. Scale bar = 250 μm, *n* = 10 biological replicates.C–FRepresentative immunohistochemistry staining images and quantification of Ly6G (C, D) and Ly6C (E, F) in aortic roots (scale bar = 25 μm, *n* = 10 biological replicates).G–IScatter diagram (G) and quantification (H‐I) of neutrophil and monocyte in plasma measured by an automated blood cell analyzer (*n* = 10 biological replicates). SFL, side fluorescence; SSC, side scatter.J, KProtein (J) and mRNA (K) levels of inflammatory cytokines TNF‐α and IL‐6 in serum and aortas. The values of mRNA levels were normalized to *Rn18s* (*n* = 10 biological replicates).LmRNA levels of proinflammatory chemokines and adhesion molecules in aortas (*n* = 10 biological replicates). The values were normalized to *Rn18s*. Data information: Data were shown as mean ± SEM; *P < 0.05; ns, not significant, two‐tailed unpaired Student's *t*‐test. Source data are available online for this figure.

Considering that immune cell recruitment and inflammation are major promoters of atherosclerotic progression (Hansson *et al*, 2006), we examined the inflammatory indexes in these mice. Immunofluorescence staining showed that macrophage‐specific DCLK1 deletion significantly diminished the infiltration of F4/80‐positive macrophages into atherosclerotic lesions in HFD‐fed ApoE^−/−^ mice (Fig 3A and B). We also found that macrophage‐specific DCLK1 deletion reduces the level of F4/80‐ and iNOS‐positive proinflammatory macrophages in atherosclerotic lesions of HFD‐fed ApoE^−/−^ mice (Appendix Fig S3). Meanwhile, immunohistochemical results revealed that the recruitment of Ly6G‐positive neutrophils (Fig 3C and D) and Ly6C‐positive monocytes (Fig 3E and F) into atherosclerotic plaques was also significantly restrained by DCLK1 deletion, which was consistent with the quantitative analysis of neutrophils and monocytes in the plasma (Fig 3G–I). Macrophage‐specific DCLK1 deletion also reduced HFD‐increased serum proinflammatory cytokines (TNF‐α and IL‐6) at both the mRNA and protein levels in ApoE^−/−^ mice (Fig 3J and K). Similar changes were observed when we examined the mRNA levels of proinflammatory cytokines (*Il1β*, *Il18*), chemokines (*Cxcl1*, *Ccl2*), and adhesion molecules (*Icam1*, *Vcam1*) in mouse atherosclerotic aortas (Fig 3L and Appendix Fig S4). Together, these results indicate that macrophage‐specific DCLK1 deletion inhibits inflammatory cell infiltration and moderates the inflammatory response in the aorta of HFD‐fed ApoE^−/−^ mice.

### DCLK1 deletion attenuates inflammatory response in macrophages via inhibiting NF‐κB activation

**Figure 4:**
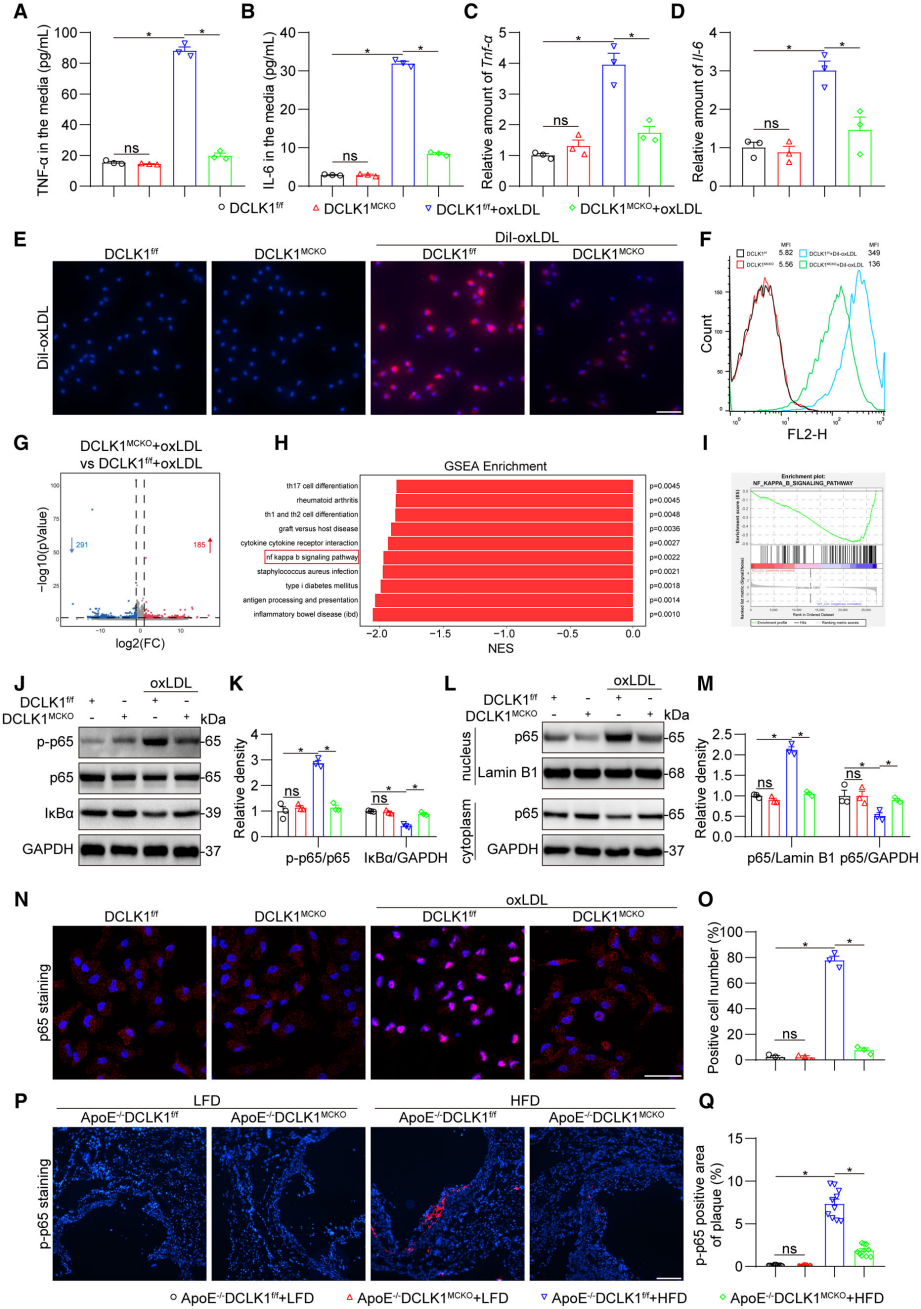
DCLK1 deletion attenuates inflammatory response in macrophages via inhibiting NF‐κB activation A, BMouse primary peritoneal macrophages (MPMs) isolated from DCLK1^f/f^ and DCLK1^MCKO^ mice were challenged with oxLDL (50 μg/ml) for 24 h. Protein levels of TNF‐α (A) and IL‐6 (B) were analyzed using ELISA (*n* = 3 biological replicates).C, DMPMs isolated from DCLK1^f/f^ and DCLK1^MCKO^ mice were challenged with oxLDL (50 μg/ml) for 6 h. mRNA levels of *Tnf‐α* (C) and *Il‐6* (D) were determined via RT‐qPCR (*n* = 3 biological replicates). The values were normalized to *β‐actin*.E, FMPMs isolated from DCLK1^f/f^ and DCLK1^MCKO^ mice were challenged with DiI‐oxLDL (50 μg/ml) for 24 h. Fluorescence staining (E) of DiI‐oxLDL (red) in MPMs. Cells were counterstained with DAPI (blue, scale bar = 25 μm). Flow cytometry analysis (F) of DiI‐oxLDL in MPMs.GMPMs isolated from DCLK1^f/f^ and DCLK1^MCKO^ mice were challenged with oxLDL (50 μg/ml) for 6 h. Total RNA was sequenced to identify differentially expressed genes (DEGs). Volcano plot analysis of DEGs up‐regulated in DCLK1^MCKO^ + oxLDL group compared to DCLK1^f/f^ + oxLDL group (red) and down‐regulated in DCLK1^MCKO^ + oxLDL group compared to DCLK1^f/f^ + oxLDL group (blue). FC, fold change.HGene‐set enrichment analysis (GSEA) of signaling pathways enriched in DCLK1^f/f^ + oxLDL versus DCLK1^MCKO^ + oxLDL group.INF‐κB signaling pathway is enriched in GSEA of DCLK1^f/f^ + oxLDL versus DCLK1^MCKO^ + oxLDL group. (J‐K) MPMs isolated from DCLK1^f/f^ and DCLK1^MCKO^ mice were challenged with oxLDL (50 μg/ml) for 1 h.J, KWestern blot analysis (J) and densitometric quantification (K) of p‐p65 and IκBα. GAPDH and p65 were used as loading controls (*n* = 3 biological replicates).L, MMPMs were treated as described in panel (J). Western blot analysis (L) and densitometric quantification (M) of p65 in nucleus and cytoplasm. GAPDH was used as the loading control for cytosolic fractions. Lamin B1 was used as the loading control for nuclear fractions (*n* = 3 biological replicates).N, OMPMs were treated as described in panel (J). Representative immunofluorescence staining images (N) and quantification (O) of NF‐κB p65 (red) translocating into nucleus in MPMs. Cells were counterstained with DAPI (blue). Scale bar = 25 μm, *n* = 3 biological replicates.P, QRepresentative immunofluorescence staining images (P) and quantification (Q) of p‐p65 (red) in aortic roots. Tissues were counterstained with DAPI (blue). Scale bar = 100 μm, *n* = 10 biological replicates. p‐p65 area was quantified by the proportion of p‐p65 positive area to plaque area. Data information: Data were shown as mean ± SEM; *P < 0.05; ns, not significant, two‐tailed unpaired Student's *t*‐test. Source data are available online for this figure.

To validate the regulation of macrophage DCLK1 on inflammation, mouse primary peritoneal macrophages (MPMs) were isolated from DCLK1^f/f^ and DCLK1^MCKO^ mice and challenged with oxLDL. As expected, DCLK1 deficiency inhibited oxLDL‐induced upregulation of inflammatory cytokines TNF‐α and IL‐6 at both mRNA and protein levels in MPMs (Fig 4A–D). Subsequently, we found that DCLK1 deletion substantially impeded oxLDL uptake in oxLDL‐challenged macrophages (Fig 4E and F), leading to less foam cell formation. Next, to explore the mechanism by which DCLK1 regulates inflammation in macrophages, we performed RNA sequencing analysis using oxLDL‐stimulated MPMs from DCLK1^f/f^ and DCLK1^MCKO^ mice (Fig 4G). A GSEA enrichment analysis of the RNA‐sequencing data indicated that the anti‐inflammatory effect of DCLK1 deficiency may be related to the NF‐κB signaling pathway (Fig 4H and I), a canonical transcriptional factor regulating inflammation and involved in inflammatory atherosclerosis (Monaco *et al*, 2004). We confirmed the effects of DCLK1 on NF‐κB through examining IκB degradation and NF‐κB p65 subunit phosphorylation and nuclear translocation. The data in Fig 4J–M show that DCLK1 knockout suppressed oxLDL‐induced IκB degradation and p65 phosphorylation and nuclear transclocation in MPMs. Immunofluorescence staining assay also showed significantly reduced nuclear p65 level in oxLDL‐challenged DCLK1‐deficient MPMs (Fig 4N and O). In addition, the immunofluorescence staining for phosphorylated p65 (p‐p65) in aortic roots further verified that DCLK1 deletion suppressed NF‐kB p65 activation in atherosclerotic mice (Fig 4P and Q). Taken together, these data demonstrate that DCLK1 deletion attenuates inflammatory response by inhibiting NF‐κB activation in macrophages.

### DCLK1 directly interacts with IKKβ to promote NF‐κB activation

**Figure 5:**
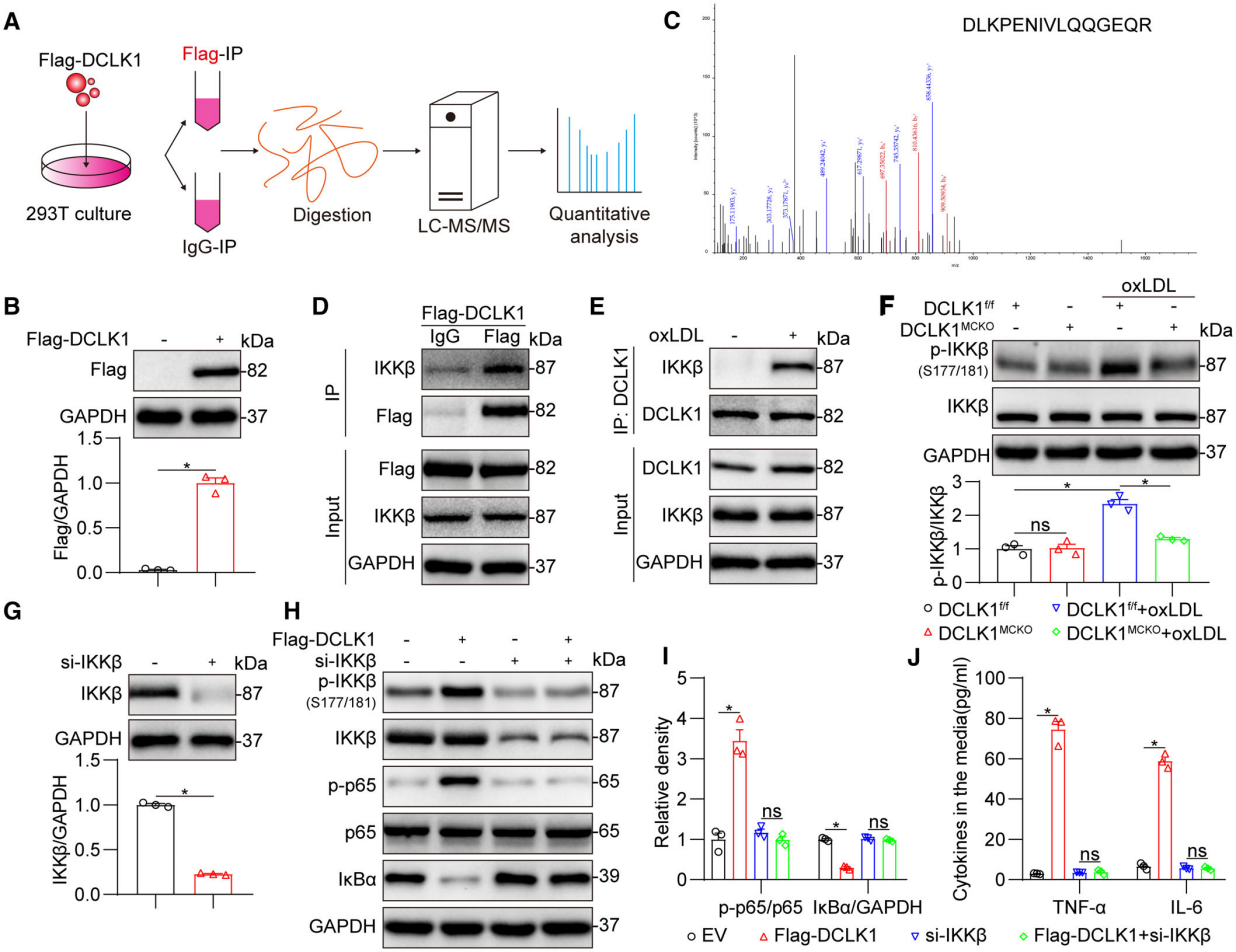
DCLK1 directly interacts with IKKβ to promote phosphorylation of IKKβ at S177/181 ASchematic illustration of quantitative proteomic screen to identify proteins binding to DCLK1.B293T cells were transfected with Flag‐DCLK1 plasmid for 24 h. Control cells were transfected with empty vector (EV). Levels of Flag were measured by Western blot (*n* = 3 biological replicates).CMS/MS spectrum of the peptide showing DLKPENIVLQQGEQR from DCLK1.DCo‐immunoprecipitation of DCLK1 and IKKβ in 293T cells transfected with Flag‐DCLK1. Flag‐DCLK1 was immunoprecipitated by anti‐Flag antibody. IgG, immunoglobulin G.ECo‐immunoprecipitation of DCLK1 and IKKβ in MPMs challenged with oxLDL (50 μg/ml) for 1 h. DCLK1 was immunoprecipitated by anti‐DCLK1 antibody.FMPMs isolated from DCLK1^f/f^ and DCLK1^MCKO^ mice were challenged with oxLDL (50 μg/ml) for 1 h. Western blot analysis and densitometric quantification of p‐IKKβ. GAPDH and IKKβ were used as loading controls (*n* = 3 biological replicates).G293T cells were transfected with IKKβ siRNA (si‐IKKβ) for 24 h, while control cells were transfected with negative control (NC) siRNA. Levels of IKKβ protein were measured by Western blot (*n* = 3 biological replicates).H, I293T cells were co‐transfected with Flag‐DCLK1 and si‐IKKβ for 24 h. Western blot analysis (H) and densitometric quantification (I) of IκBα, p‐IKKβ and p‐p65. GAPDH, IKKβ and p65 were used as loading controls (*n* = 3 biological replicates).J293T cells were co‐transfected with Flag‐DCLK1 and si‐IKKβ for 48 h. Protein levels of TNF‐α and IL‐6 were analyzed using ELISA (*n* = 3 biological replicates). Data information: Data were shown as mean ± SEM; *P < 0.05; ns, not significant, two‐tailed unpaired Student's *t*‐test. Source data are available online for this figure.

To further decipher how DCLK1 activates NF‐κB, we performed LC–MS/MS analysis using 293T cells transfected with Flag‐DCLK1 plasmid (Fig 5A and B). Interestingly, the mass spectrometry data identified IKKβ, a canonical and direct upstream kinase of NF‐κB (Hayden & Ghosh, 2008), as a binding protein of DCLK1 (Fig 5C). As a upstream of NF‐κB, IKKβ has also been reported to be involved in atherosclerosis (Strnad & Burke, 2007). Therefore, we hypothesize that DCLK1 regulates NF‐κB activation and inflammatory atherosclerosis by directly binding to IKKβ.

First, the interaction between DCLK1 and IKKβ was confirmed in 293T cells transfected with Flag‐DCLK1 by coimmunoprecipitation (Fig 5D). The DCLK1‐IKKβ complex was further found in oxLDL‐treated MPMs using coimmunoprecipitation assay (Fig 5E). Considering that DCLK1 is a protein kinase (Nevi *et al*, 2021), we hypothesize that the DCLK1‐IKKβ interaction may phosphorylate IKKβ. Two phosphorylating sites, serine 177 and serine 181 (S177/181), are critical for IKKβ phosphorylation (Delhase *et al*, 1999; Scheidereit, 2006). As shown in Fig 5F, oxLDL challenging induced IKKβ phosphorylation at S177/181, while DCLK1 deletion significantly reduced this change in MPMs. We then explored whether IKKβ is indispensable for DCLK1‐mediated NF‐κB activation and inflammatory response. We knocked IKKβ expression down in 293T cells using IKKβ siRNA (Fig 5G). These cells were simultaneously transfected with Flag‐DCLK1 plasmid to induce DCLK1 overexpression. As shown in Fig 5H–J, DCLK1 overexpression in 293T cells could increase the phosphorylation level of IKKβ at S177/181 and induce NF‐κB activation and inflammatory cytokine production. However, knocking down IKKβ completely reversed the NF‐κB activation and inflammatory cytokine production induced by DCLK1 overexpression in 293T cells (Fig 5H–J). These results show that DCLK1 directly interacts with IKKβ to promote the phosphorylation of IKKβ at S177/181 and NF‐κB‐mediated inflammation.

### Pharmacological inhibitor of DCLK1 mitigates NF‐κB activation and inflammatory response in oxLDL‐challenged macrophages

**Figure 6:**
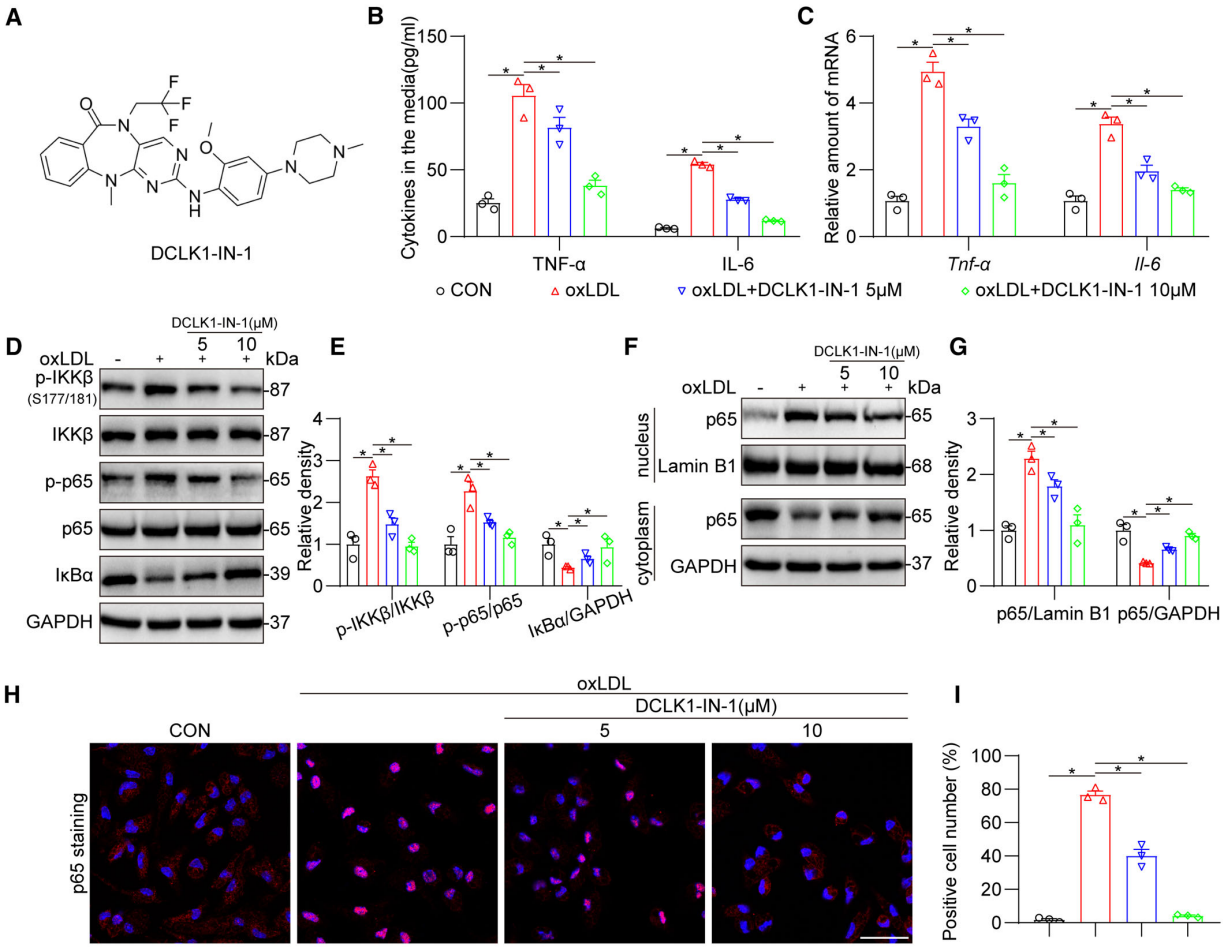
Pharmacological inhibitor of DCLK1 mitigates NF‐κB and inflammatory response in macrophages AThe chemical structure of DCLK1‐IN‐1.BMPMs were pretreated with DCLK1‐IN‐1 (5 and 10 μM) or vehicle (DMSO, 1‰) for 1 h, followed by exposure of oxLDL (50 μg/ml) for 24 h. Protein levels of TNF‐α and IL‐6 were analyzed using ELISA (*n* = 3 biological replicates).CMPMs were pretreated with DCLK1‐IN‐1 (5 and 10 μM) or vehicle (DMSO, 1‰) for 1 h, followed by exposure of oxLDL (50 μg/ml) for 6 h. mRNA levels of *Tnf‐α* and *Il‐6* were determined via RT‐qPCR (*n* = 3 biological replicates). The values were normalized to *β‐actin*.D, EMPMs were pretreated with DCLK1‐IN‐1 (5 and 10 μM) or vehicle (DMSO, 1‰) for 1 h, followed by exposure of oxLDL (50 μg/ml) for 1 h. Western blot analysis (D) and densitometric quantification (E) of IκBα, p‐IKKβ and p‐p65. GAPDH, IKKβ and p65 were used as loading controls (*n* = 3 biological replicates).F, GMPMs were treated as described in panel (E). Western blot analysis (F) and densitometric quantification (G) of p65 in nucleus and cytoplasm. GAPDH was used as the loading control for cytosolic fractions. Lamin B1 was used as the loading control for nuclear fractions (*n* = 3 biological replicates).H, IMPMs were treated as described in panel (D). Representative immunofluorescence staining images (H) and quantification (I) of NF‐κB p65 (red) translocating into nucleus in MPMs. Cells were counterstained with DAPI (blue). Scale bar = 25 μm, *n* = 3 biological replicates. Data information: Data were shown as mean ± SEM; *P < 0.05, two‐tailed unpaired Student's *t*‐test. Source data are available online for this figure.

To strengthen our findings, a selective small‐molecule inhibitor of DCLK1, DCLK1‐IN‐1 (Fig 6A; Ferguson *et al*, 2020) was used. The dose of DCLK1‐IN‐1 (5 and 10 μM) was selected based on previous studies (Ding *et al*, 2021; Patel *et al*, 2021). As expected, DCLK1‐IN‐1 treatment dose‐dependently inhibited oxLDL‐induced upregulation of pro‐inflammatory cytokines at both mRNA and protein levels in MPMs (Fig 6B and C). We also found that DCLK1‐IN‐1 significantly inhibited IKKβ phosphorylation at S177/181, p65 phosphorylation, IkBa degradation, and p65 nuclear translocation in oxLDL‐challenged macrophages (Fig 6D–I). These results demonstrate that the pharmacological inhibition of DCLK1 mitigates NF‐κB activation and inflammatory response in macrophages.

### Pharmacological inhibitor of DCLK1 prevents atherosclerotic progression and inflammation in mice

**Figure 7:**
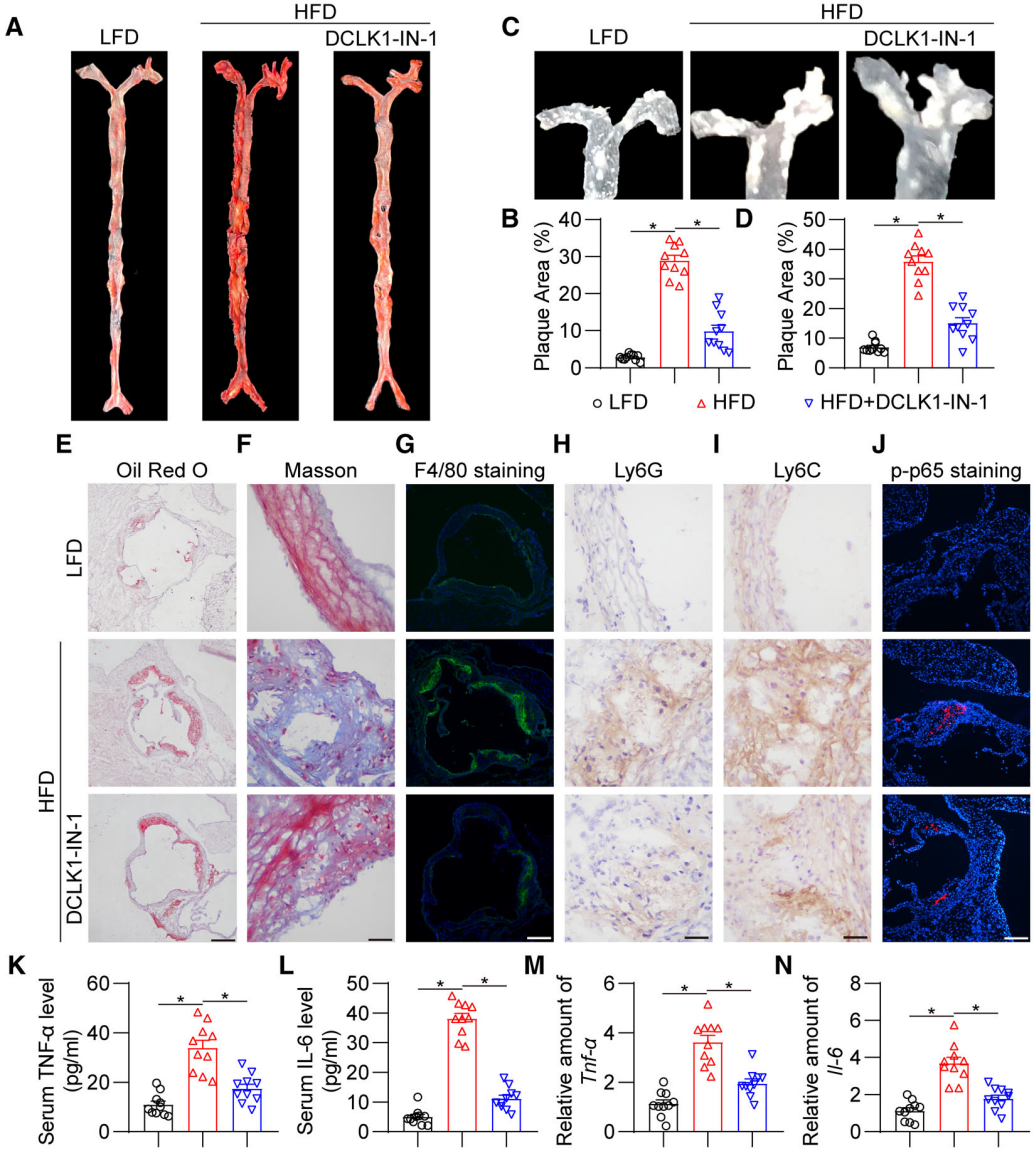
Pharmacological inhibitor of DCLK1 prevents atherosclerotic progression and inflammation in mice The animal experiment using HFD‐fed ApoE^−/−^ mice treated with or without DCLK1‐IN‐1 was described in the Materials and Methods section.
A, BRepresentative en face Oil Red O staining (A) and quantification (B) of Oil Red O‐positive plaque lesion area in aortas. Plaque area was defined as percentage of total surface area of the aorta (*n* = 10 biological replicates).C, DRepresentative images (C) and quantification (D) of plaque lesion area in aortic arches (*n* = 10 biological replicates). The plaque area was quantified by the proportion of plaque area to aortic arches area.ERepresentative images of Oil Red O staining of atherosclerotic lesion in aortic roots (scale bar = 250 μm).FRepresentative images of Masson's Trichrome staining for collagen deposition in aortic roots (scale bar = 25 μm).GRepresentative immunofluorescence staining images of F4/80 (green) in aortic roots. Tissues were counterstained with DAPI (blue). Scale bar = 250 μm.H, IRepresentative immunohistochemistry staining images of Ly6G (H) and Ly6C (I) in aortic roots (scale bar = 25 μm).JRepresentative immunofluorescence staining images of p‐p65 (red) in aortic roots. Tissues were counterstained with DAPI (blue). Scale bar = 100 μm.K–NProtein (K, L) and mRNA (M, N) levels of inflammatory cytokines TNF‐α and IL‐6 in serum and aortas. The values of mRNA levels were normalized to *Rn18s* (*n* = 10 biological replicates). Data information: Data were shown as mean ± SEM; *P < 0.05, two‐tailed unpaired Student's *t*‐test. Source data are available online for this figure.

DCLK1‐IN‐1 was further used in *in vivo* experiments to enhance a translational significance. The dose of DCLK1‐IN‐1 (10 mg/kg/day) was selected based on a previous study (Ferguson *et al*, 2020). As shown in Appendix Fig S5A–D, DCLK1‐IN‐1 treatment did not affect the increased body weight and serum lipid profile in HFD‐fed ApoE^−/−^ mice. However, DCLK1‐IN‐1 substantially decreased the plaque area in the aorta of HFD‐fed ApoE^−/−^ mice (Fig 7A–D). Similarly, Oil Red O staining of the aortic roots showed that DCLK1‐IN‐1 attenuated atherosclerotic lesions (Fig 7E; Appendix Fig S6A). Masson's trichome staining also revealed reduced collagen deposition in atherosclerotic plaques in the DCLK1‐IN‐1‐treated group (Fig 7F; Appendix Fig S6B). Immunofluorescence and immunohistochemical staining showed that DCLK1‐IN‐1 significantly reduced the infiltration of inflammatory cells (macrophages, neutrophils, and monocytes) into atherosclerotic lesions in HFD‐fed ApoE^−/−^ mice (Fig 7G–I; Appendix Fig S6C–E). The staining of p‐p65 in aortic roots showed that DCLK1‐IN‐1 constrained NF‐kB activation in the aortic roots of atherosclerotic mice (Fig 7J; Appendix Fig S6F), which was consistent with the *in vitro* results. Finally, DCLK1‐IN‐1 reduced the levels of proinflammatory cytokines (TNF‐α, IL‐6) upregulated in HFD‐fed ApoE^−/−^ mice (Fig 7K–N). Collectively, these results suggest that pharmacological inhibitor of DCLK1 significantly prevent atherosclerotic progression and inflammation in mice.

## Discussion

In this study, we evaluated the role of DCLK1 in atherosclerosis both *in vivo* and *in vitro*. The three key findings were as follows: (i) macrophage‐specific deletion or pharmacological inhibition of DCLK1 attenuates atherosclerotic progression; (ii) macrophage‐specific deletion or pharmacological inhibition of DCLK1 alleviates chronic inflammation by inhibiting NF‐κB activation; and (iii) DCLK1 directly binds to IKKβ and phosphorylates IKKβ at S177/181.

Known functions of DCLK1 are almost exclusively derived from studies in neurogenesis (Koizumi *et al*, 2006; Shu *et al*, 2006) and carcinogenesis (Chandrakesan *et al*, 2017; Nevi *et al*, 2021). Increased levels of DCLK1 in various human cancers including renal (Ge *et al*, 2018), pancreatic (Bailey *et al*, 2014), colorectal (Sureban *et al*, 2009), and liver (Nevi *et al*, 2021) have been correlated with cellular activities such as epithelial‐to‐mesenchymal transition, and cell proliferation and migration. However, a few recent studies (Yi *et al*, 2019) reporting DCLK1 in colitis promoted us to test whether DCLK1 plays a role in inflammatory diseases, such as atherosclerosis. Our studies show that DCLK1 is increased in infiltrated macrophages in atherosclerotic lesions of HFD‐fed ApoE^−/−^ mice. This elevated expression of DCLK1 in macrophages mediated oxLDL‐induced inflammatory responses, which subsequently promotes the pathogenesis of atherosclerosis. *In vitro* assays showed that DCLK1 deletion and DCLK1‐IN‐1 treatment reduced inflammatory factors in primary macrophages. Macrophage‐specific DCLK1 deletion and the pharmacological inhibitor DCLK1‐IN‐1 significantly prevented atherosclerotic progression in HFD‐fed ApoE^−/−^ mice by suppressing inflammatory cell infiltration and alleviating the inflammatory response in the aorta. Thus, this is the first time to identify a new function of DCLK1 in inflammatory atherosclerosis and show that DCLK1 mediates oxLDL‐induced inflammation in macrophages.

The inflammatory response involves atherosclerosis from atherosclerotic inception to the emergence of complications (Soehnlein & Libby, 2021). During the symptom phase, inflammatory mediators regulate the progression of atheroma, plaque disruption, and healing process (Libby, 2021). Macrophages play critical roles in all phases of atherosclerosis, from lesion origination and expansion to necrosis resulting in the rupture and clinical symptoms of atherosclerosis (Tabas & Bornfeldt, 2016, 2020; Koelwyn *et al*, 2018). In atherosclerosis, infiltrated and activated macrophages secrete proinflammatory chemokines and cytokines that in turn facilitate further recruitment of inflammatory cells (neutrophils, monocytes, and macrophages) into the lesion area (Back *et al*, 2019; Wolf & Ley, 2019). These inflammatory cells promote plaque growth and make plaques become more advanced (Doran, 2022; Engelen *et al*, 2022). Thus, pathological processes linked to macrophages are significant targets for diagnostic imaging as well as innovative treatments for atherosclerosis, resulting in enhanced inflammation resolution and plaque stability. Nuclear factor kappa B (NF‐κB) is a classical transcription factor that modulates the expression of a plethora of inflammatory genes (Baker *et al*, 2011). The NF‐κB p65 subunit, which is usually sequestered in the cytoplasm by IκBα, is released and translocated into the nucleus. Phosphorylated NF‐κB p65 binds to DNA and induces the transcription of its target inflammatory genes (Hayden & Ghosh, 2004). NF‐κB activation has been observed in atherosclerotic lesions in both human and animal models (Hernandez & Zhou, 2021). In addition, the NF‐κB signaling pathway in atherosclerosis has been identified to be IKKβ‐dependent, which is related to an increase in proinflammatory and prothrombotic responses (Monaco *et al*, 2004). IKKβ is involved in many chronic inflammatory diseases such as atherosclerosis (Karin & Delhase, 2000; Strnad & Burke, 2007; Durand & Baldwin, 2017). Park *et al* (2012) reported that IKKβ deletion in macrophages moderated atherosclerosis in mice. Thus, IKKβ may be a therapeutic target for the clinical management of atherosclerosis. In the present study, using pulldown assays and HPLC‐tandem MS, we report that DCLK1 directly interacts with IKKβ. Interaction of DCLK1 with IKKβ was reduced by DCLK1‐IN‐1, suggesting that the binding site may be common. Moreover, we demonstrated that the interaction of DCLK1 with IKKβ induces IKKβ phosphorylation at S177/181 to facilitate NF‐κB activation and the subsequent secretion of inflammatory mediators in oxLDL‐challenged macrophages. These studies have provided empirical evidencing linking DCLK1 to inflammatory responses and have identified a mechanism involving IKKβ/NF‐κB.

This study has some limitations. The specific domains of DCLK1 and IKKβ that interact with each other have not been determined. Although we observed that DCLK1 phosphorylated IKKβ at S177/181, which is located in the activation loop of IKKβ and is essential for IKKβ phosphorylation (Delhase *et al*, 1999; Scheidereit, 2006), we could not confirm whether DCLK1 also phosphorylates other sites of IKKβ. Structurally, the N‐terminal region of DCLK1, which includes a tandem DCX domain, drives the microtubule‐associating function, while the C‐terminal region, harboring a serine/threonine kinase domain, is highly related to a Ca^2+^/calmodulin‐dependent protein 1 kinase domain, despite lacking the canonical calmodulin‐binding site. The exact binding mode needs to be uncovered using DCLK1‐IKKβ crystal structural biology. Another unanswered question arising from our study is how oxLDL/hyperlipidemia upregulates DCLK1 in macrophages. Currently, no physiological activators of DCLK1 have been reported. In nervous system, a neuronal calcium sensor family member HPCAL1 has been suggested as an activator of DCLK1 (Cheng *et al*, 2022). Although the mechanisms need to be identified upstream of DCLK1, it is possible that increased expression of DCLK1 is all that is needed. We also show that overexpression of DCLK1 induces IKKβ/NF‐κB activation and inflammatory gene expression.

In conclusion, the current study identified upregulated DCLK1 in macrophages in atherosclerotic lesions and showed that macrophage‐specific deletion and pharmacological inhibition of DCLK1 attenuated atherosclerotic progression by inhibiting NF‐κB‐mediated inflammation both *in vivo* and *in vitro*. Mechanistically, we found that DCLK1 directly interacted with IKKβ and phosphorylated IKKβ at S177/181. This finding extends our understanding of the role of DCLK1 in inflammatory regulation and indicates that DCLK1 is a potential therapeutic target for retarding atherosclerotic progression.

## Materials and Methods

### Reagents

Oxidized low‐density lipoproteins (oxLDL) and DiI‐labeled oxLDL (DiI‐oxLDL) were purchased from Peking Union‐Biology (Beijing, China). Antibodies against GAPDH (#5174, 1:1,000), β‐actin (#3700, 1:1,000), p‐IKKβ (#2697, 1:1,000), IKKβ (#8943, 1:1,000), p‐p65 (#3033, 1:1,000 for western blotting and 1:200 for immunofluorescence staining), NF‐κB p65 (#8242, 1:1,000), and IκBα (#9242, 1:1,000) were purchased from Cell Signaling Technology (Danvers, MA, USA). Antibodies against DCLK1 (#ab31704, 1:1,000 for western blotting and 1:200 for immunofluorescence staining), F4/80 (#ab6640, 1:200), CD31 (#ab9498, 1:200), α‐SMA (#ab7817, 1:200), iNOS (#ab178945, 1:200), and Lamin B1 (#ab133741, 1:1,000) were purchased from Abcam (Cambridge, UK). Antibodies against Ly6G (#sc‐53515, 1:100) and Ly6C (#sc‐271811, 1:100) were purchased from Santa Cruz Biotechnology (Santa Cruz, CA, USA). The antibody against Flag (#20543‐1‐AP, 1:1,000) was purchased from Proteintech (Wuhan, China). DCLK1‐IN‐1 (BD01203503) was purchased from Bidepharm (Shanghai, China).

### Animal experiments

All animal care and experimental procedures were approved by the Wenzhou Medical University Animal Policy and Welfare Committee (approval ID: wydw2021‐0057). All animal experiments followed the Guide for the Care and Use of Laboratory Animals (National Institutes of Health, USA). Wildtype C57BL/6 (Strain NO. N000013), ApoE knockout mice on a C57BL/6 background, B6/JGpt‐Dclk1em1Cflox/Gpt (DCLK1‐flox) mice, and B6/JGpt‐Lyz2em1Cin(iCre)/Gpt (lysozyme 2 (Lyz2)‐driven Cre) mice were obtained from GemPharmatech. Myeloid cell‐specific DCLK1 knockout mice (DCLK1‐Lyz^Cre^, DCLK1^MCKO^) were generated with technical expertise from GemPharmatech Co. Ltd (Nanjing, China). Then, the DCLK1‐flox (DCLK1^f/f^), DCLK1^MCKO^, and ApoE‐knockout (ApoE^−/−^) mice on a C57BL/6 background were further generated in Gempharmatech (Nanjing, China). ApoE^−/−^ mice were crossed with DCLK1^MCKO^ mice to generate ApoE^−/−^DCLK1^f/f^ and ApoE^−/−^DCLK1^MCKO^ mice, respectively. Validation of genotype of ApoE^−/−^DCLK1^f/f^ and ApoE^−/−^DCLK1^MCKO^ mice was performed by PCR using the gene primers in Appendix Table S1. Animals were housed in a 12:12 h light–dark cycle at a constant room temperature and fed a standard rodent diet. The animals were acclimatized to the laboratory for at least 2 weeks before initiating the studies. All animal experiments were performed and analyzed by blinded researchers.

For studies using DCLK1 knockout mice, male eight‐week‐old ApoE^−/−^DCLK1^f/f^ and ApoE^−/−^DCLK1^MCKO^ mice were fed an LFD containing 10 kcal.% fat, 20 kcal.% protein and 70 kcal.% carbohydrate (H10010, HFK Bioscience, Beijing, China) or a HFD containing 40 kcal.% fat, 20 kcal.% protein, 40 kcal.% carbohydrate and 1.25% cholesterol (H10540, HFK Bioscience) for 16 weeks, respectively. LFD and HFD groups were assigned in a randomized fashion. Body weight was recorded weekly. All mice were sacrificed under sodium pentobarbital anesthesia, and blood and aortas samples were collected.

For studies involving DCLK1 inhibitor DCLK1‐IN‐1, eight‐week‐old ApoE^−/−^ mice were randomly divided into three groups: (i) LFD group: mice fed with a LFD and intragastrically treated with 1% CMC‐Na vehicle control; (ii) HFD group: mice fed with a HFD and intragastrically treated with 1% CMC‐Na vehicle control; and (iii) HFD + DCLK1‐IN‐1 group: mice fed with a HFD and intragastrically treated with 10 mg/kg/2 days DCLK1‐IN‐1 reconstituted in 1% CMC‐Na solution. All mice were fed with a LFD or HFD for total 16 weeks, while mice were treated with the vehicle or DCLK1‐IN‐1 only for the final 8 weeks. Body weight was recorded weekly. All mice were sacrificed under sodium pentobarbital anesthesia at week 16, and blood samples were collected. Aortas were fixed in 4% paraformaldehyde or snap‐frozen in liquid nitrogen.

### Atherosclerotic lesion analysis

For en face lesion analysis of the aorta, the whole aorta and aortic sinus were dissected, opened longitudinally from the heart to the iliac arteries, and stained with Oil Red O (G1260, Solarbio, Beijing, China). The heart and proximal aorta were collected and embedded in optimum cutting temperature compound for quantification of plaque lesions. Serial 5 μm‐thick cryosections of the aortic sinus were obtained from each mouse. The sections were stained with Oil Red O and hematoxylin for plaque size analysis.

Paraffin‐embedded sections were used for Ly6G and Ly6C immunohistochemistry and Masson's trichrome staining (G1340, Solarbio). For immunohistochemistry, sections were deparaffinized and rehydrated, followed by heat‐induced antigen retrieval using 10 mM sodium citrate buffer (pH 6.5). Sections were blocked with 3% H_2_O_2_ and then with 5% bovine serum albumin for 30 min. Primary Ly6G (1:200) and Ly6C (1:200) antibodies were added. Sections were incubated at 4°C overnight. Horseradish peroxidase‐conjugated secondary antibodies and DAB were used for detection.

Frozen sections were used for immunofluorescence staining. Slides were fixed in cold methanol and permeabilized using 0.5% Triton‐X. Then, slides were blocked using 5% bovine serum albumin for 30 min and incubated overnight with primary antibodies. Alexa‐488 and Alexa‐647 conjugated secondary antibodies (Abcam, 1:200) were used for detection. Images were captured using a fluorescence microscope (Nikon, Tokyo, Japan).

### Analysis of leukocytes in plasma

Plasma neutrophils and monocytes were analyzed using an automated blood cell analyzer (XN‐1000, Sysmex, Kobe, Japan).

### Cytokine measurements

The levels of TNF‐α and IL‐6 in the serum and cell culture media were determined using ELISA kits (Cat#. 88‐7324‐76, 88‐7064‐76, 88‐7346‐76 and 88‐7066‐76, Thermo Fisher, Carlsbad, CA, USA).

### Cell culture

Mouse primary peritoneal macrophages (MPMs) were isolated as described previously (Chen *et al*, 2020; Huang *et al*, 2022). Briefly, mice received a single intraperitoneal injection of 6% thioglycolate solution. Two days later, the mice were euthanized, and the peritoneal cavity was flushed with RPMI‐1640 medium (Gibco, Eggenstein, Germany). Samples were centrifuged, and the cell suspension was plated in RPMI‐1640 medium containing 10% fetal bovine serum (FBS, Gibco) and 1% penicillin/streptomycin (Invitrogen, Waltham, MA, USA). Nonadherent cells were removed 2 h after seeding the cell suspension. 293T cells (GNHu17) were purchased from Shanghai Institute of Biochemistry and Cell Biology (Shanghai, China) and cultured in high‐glucose Dulbecco's modified Eagle's medium (DMEM; Gibco) with 10% FBS and 1% penicillin/streptomycin. All cells were cultured in a humidified incubator maintained at 37°C and 5% CO_2_.

### Gene knockdown and overexpression

Gene silencing and overexpression in cells were achieved by transfecting specific siRNAs and plasmids. The custom siRNA synthesized for human IKKβ (5′‐ GGAUUACAUUAGUGGACAATT‐3′) was purchased from RiboBio (Guangzhou, China). Plasmid encoding Flag‐DCLK1 was constructed by Genechem (Shanghai, China). Transfection of 293T cells with siRNA and plasmid was performed using Lipofectamine 2000 (Thermo Fisher Scientific, Carlsbad, CA, USA).

### oxLDL uptake assay

For uptake detection, MPMs were incubated 50 μg/ml DiI‐oxLDL for 3 h at 37°C. Cells were washed with PBS and imaged using a Leica TCS SP8 confocal laser‐ scanning microscope (Buffalo Grove, IL, USA). Cells under identical conditions were dislodged and analyzed using flow cytometry. The results of flow cytometry were expressed as mean fluorescence intensity after subtracting the auto‐fluorescence of cells (absence of DiI‐oxLDL).

### Western blotting and co‐immunoprecipitation

Total protein from cells and aortic tissues was extracted using lysis buffer (AR0103, Boster Biological Technology, Pleasanton, CA, USA). Proteins were separated by 10% sodium dodecyl sulfate‐polyacrylamide gel electrophoresis (SDS–PAGE) and transferred to polyvinylidene fluoride membranes. Before adding specific primary antibodies, the membranes were blocked in Tris‐buffered saline (pH 7.4, containing 0.1% Tween 20 and 5% nonfat milk) for 1.5 h at room temperature. Protein bands were detected by incubation with horseradish peroxidase‐conjugated secondary antibodies and an enhanced chemiluminescence reagent (Bio‐Rad, Hercules, CA, USA). Band densities were quantified using ImageJ software (version 1.38 e, NIH, Bethesda, MD, USA) and normalized to the loading controls.

For co‐immunoprecipitation assays, cell extracts prepared following treatments were incubated with indicated antibodies at 4°C overnight. Then the proteins were immunoprecipitated with Protein A + G Agarose (P2012, Beyotime, Shanghai, China) at 4°C for 2 h. Immunoprecipitation samples were immunoblotted for co‐precipitated protein detection. Total lysates were subjected to western blot analysis as input controls. Protein interactions were quantified using ImageJ software.

### Real‐time quantitative PCR

Total RNA was extracted from cells or aortic tissues using RNAiso Plus (9109, Takara, Shiga, Japan). Reverse transcription was performed using the PrimeScript™ RT Reagent Kit with gDNA Eraser (RR047A, Takara, Shiga, Japan). Quantitative PCR was performed using TB Green® Premix Ex Taq™ II (RR820A; Takara, Shiga, Japan) in a QuantStudio™ 3 Real‐Time PCR System (Thermo Fisher Scientific, Carlsbad, CA, USA). The efficiency of PCR amplification was required to be 90–110%. The CT values were normalized to *Rn18s* or *β‐actin* and the $2^{-\Delta \Delta C_{T}}$ method was used to calculate the relative amount of target mRNA. The primers were obtained from Thermo Fisher Scientific. The primer sequences used in this study are listed in Appendix Table S2.

### Transcriptome sequencing

Total RNA from the cells was collected using RNAiso Plus and subjected to genome‐wide transcriptomic analysis using LC‐Bio (Hangzhou, China). Differentially expressed genes (DEGs) were selected with fold change > 2 or fold change < 0.5 and *P*‐value < 0.05. Gene‐set enrichment analysis (GSEA, https://www.gsea‐msigdb.org/gsea/index.jsp) of the signaling pathways was performed as described by LC‐Bio (https://www.lc‐bio.cn/). Publicly available transcriptome data GSE94044 (Mohanta *et al*, 2022b; Data ref: Mohanta *et al*, 2022a) and GSE137581 (Guo *et al*, 2020b; Data ref: Guo *et al*, 2020a) were acquired from the Gene Expression Omnibus database (https://www.ncbi.nlm.nih.gov/geo/). DEGs were selected with fold change > 2 or fold change < 0.5 and *P*‐value < 0.05, using GEO2R.

### LC–MS/MS analysis

Anti‐Flag antibody was added to the lysate of 293T cells transfected with Flag‐DCLK1 for co‐immunoprecipitation and IgG was used as a negative control. LC–MS/MS analysis was performed by PTM Bio Co., Ltd (Zhejiang, China). We screened the substrate proteins that may bind to DCLK1 according to the score and Flag/IgG ratio of the detected proteins in the mass spectrometry data.

### Statistical analysis

Sample sizes were defined by *a priori* power calculation with G‐Power 3.1.9 software (http://www.gpower.hhu.de/), considering a statistical power of 80% and α = 0.05. We employed a random number table to perform randomization. Briefly, all animal experiments in the present study were performed and analyzed in a blinded manner. Treatment groups were assigned in a randomized fashion. Every mouse was assigned a temporary random number within the weight range. Mice were given their permanent numerical designation in the cages after they were randomly divided into each group. For each group, a cage was selected randomly from the pool of all cages. All data were collected and analyzed by two observers who were not aware of the group assignment or treatment of the mice. Data represented at 10 biological replicates in animal experiments and three biological replicates in cell experiments and were expressed as mean ± standard error of the mean (SEM). Statistical analyses were performed using GraphPad Prism 8.0 software (GraphPad, San Diego, CA, USA). Comparisons between two groups were analyzed using two‐tailed unpaired Student's *t*‐test. One‐way ANOVA followed by Dunnett's *post‐hoc* test was used to compare more than two data groups. Statistical significance was set at *P* < 0.05. Post‐tests were run only if F achieved *P* < 0.05, and there was no significant variance in homogeneity.

## Author contributions

**Zhuqi Huang:** Data curation; investigation; writing – original draft. **Sirui Shen:** Investigation. **Xue Han:** Investigation. **Weixin Li:** Investigation. **Wu Luo:** Data curation; investigation. **Liming Lin:** Investigation. **Mingjiang Xu:** Writing – review and editing. **Yi Wang:** Writing – original draft; writing – review and editing. **Weijian Huang:** Conceptualization. **Gaojun Wu:** Conceptualization; data curation. **Guang Liang:** Conceptualization; writing – original draft; writing – review and editing.

## Disclosure and competing interests statement

The authors declare that they have no conflict of interest.

## For more information

Authors' homepage: cbrc.yxy.wmu.edu.cn.

## Supporting information



Appendix S1

Source Data for Appendix

Source Data for Figure 1

Source Data for Figure 2

Source Data for Figure 3

Source Data for Figure 4

Source Data for Figure 5

Source Data for Figure 6

Source Data for Figure 7

## Data Availability

The datasets produced in this study are available in the online and open‐access databases. RNA sequencing data: Gene Expression Omnibus GSE221647 (https://www.ncbi.nlm.nih.gov/geo/query/acc.cgi?acc=GSE221647). LC–MS/MS data: PRIDE PXD039083 (https://www.ebi.ac.uk/pride/archive/projects/PXD039083).
