# Supplementary material for: Macrophage DCLK1 promotes atherosclerosis via binding to IKKβ and inducing inflammatory responses
Source: EMBO Mol Med. 2023 Mar 10;15(5):e17198. doi: 10.15252/emmm.202217198 (PMC10165355; doi:10.15252/emmm.202217198)
Supplement: Supplementary file 1 — Appendix S1 [file EMMM-15-e17198-s008.pdf]

Appendix data

Macrophage DCLK1 promotes atherosclerosis via binding to IKKβ and inducing inflammatory responses

The appendix file includes 2 Tables and 6 Figures.

Contents

Appendix Table S1. Primer sequences for PCR genotyping analysis…………………………..2

Appendix Table S2. Primer sequences for real-time qPCR assay………………………………3

Appendix Figure S1. Generation and genotyping validation of ApoE$^{-/-}$DCLK1$^{f/f}$ and ApoE$^{-/-}$DCLK1^{MCKO} mice by PCR………………………………………………………………..4

Appendix Figure S2. DCLK1 deletion does not affect serum lipid levels in HFD-fed ApoE$^{-/-}$ mice………………………………………………………………………………………….5

Appendix Figure S3. DCLK1 deletion reduces pro-inflammatory macrophages in aortic roots of HFD-fed ApoE$^{-/-}$ mice……………………………………………………………………6

Appendix Figure S4. mRNA levels of inflammatory cytokines *Il-1β* and *Il-18* in aortas……………………………………………………………………………………...7

Appendix Figure S5. DCLK1-IN-1 does not affect serum lipid levels in HFD-fed ApoE$^{-/-}$ mice……………………………………………………………………………………….…8

Appendix Figure S6. Quantification for Figure 7E-J…………………………………..……….9

Appendix Table S1. Primer sequences for PCR genotyping analysis.

Gene	Species	Sequence
Apoe	Mouse	TGCCTAGTCTCGGCTCTGAACTAC<br>CAACCTGGGCTACACACTAATTGAG
Flox	Mouse	CCATCAAATAAAGACAGCCCAGTA<br>CCACCGCAACCCCCACCAGAC
Lyz2-Cre KI	Mouse	AGTGCTGAAGTCCATAGATCGG<br>CTGATTCTCCTCATCACCAGG
Lyz2-Cre WT	Mouse	AGTGCTGAAGTCCATAGATCGG<br>GTCACTCACTGCTCCCCTGT

Appendix Table S2. Primer sequences for real-time qPCR assay.

Gene	Species	Sequence
Tnf-α	Mouse	TGATCCGCGACGTGGAA<br>ACCGCCTGGAGTTCTGGAA
Il-6	Mouse	GAGGATACCACTCCCAACAGACC<br>AAGTGCATCATCGTTGTTCATACA
Il-1β	Mouse	GCAACTGTTCCTGAACTCAACT<br>ATCTTTTGGGGTCCGTCAACT
Il-18	Mouse	GACTCTTGCGTCAACTTCAAGG<br>CAGGCTGTCTTTTGTCAACGA
Icam1	Mouse	GTGATGCTCAGGTATCCATCCA<br>CACAGTTCTCAAAGCACAGCG
Vcam1	Mouse	AGTTGGGGATTCGGTTGTTCT<br>CCCCTCATTCCTTACCACCC
Cxcl1	Mouse	CTGGGATTCACCTCAAGAACATC<br>CAGGGTCAAGGCAAGCCTC
Ccl2	Mouse	TTAAAAACCTGGATCGGAACCAA<br>GCATTAGCTTCAGATTTACGGGT
β-actin	Mouse	GGCTGTATTCCCCTCCATCG<br>CCAGTTGGTAACAATGCCATGT
Rn18s	Mouse	AGTCCCTGCCCTTTGTACACA<br>CGATCCGAGGGCCTCACT
Dclk1	Mouse	TCCACCGGAATTGAACTCGG<br>GGGAGCGAACAGTCTCAGA

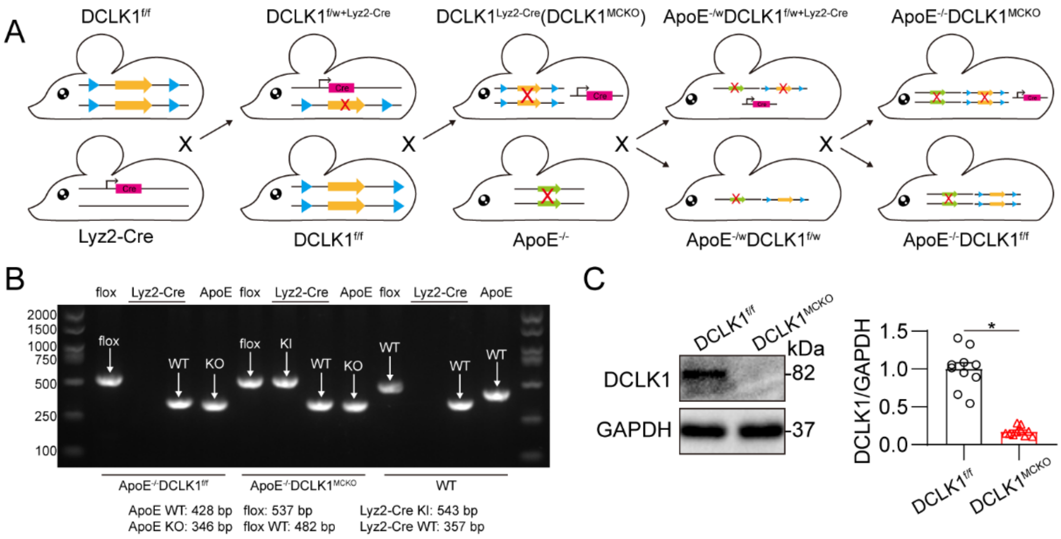

Appendix Figure S1. Generation and genotyping validation of ApoE$^{-/-}$DCLK1$^{f/f}$ and ApoE$^{-/-}$DCLK1^{MCKO} mice by PCR. (A) Diagram of the construction of ApoE$^{-/-}$DCLK1$^{f/f}$ and ApoE$^{-/-}$DCLK1^{MCKO} mice. (B) To identify the genotypes of ApoE$^{-/-}$DCLK1$^{f/f}$ and ApoE$^{-/-}$DCLK1^{MCKO} mice. cDNA was extracted from mouse tail and the primers for identification of ApoE$^{-/-}$DCLK1$^{f/f}$ and ApoE$^{-/-}$DCLK1^{MCKO} mice were used for PCR assay. (C) Western blot analysis of DCLK1 in primary macrophages isolated from DCLK1$^{f/f}$ and DCLK1^{MCKO} mice. GAPDH was used as the loading control. Densitometric quantification is shown in right (n=10 biological replicates). Data were shown as mean ± SEM; *, P < 0.05, two-tailed unpaired Student's t-test.

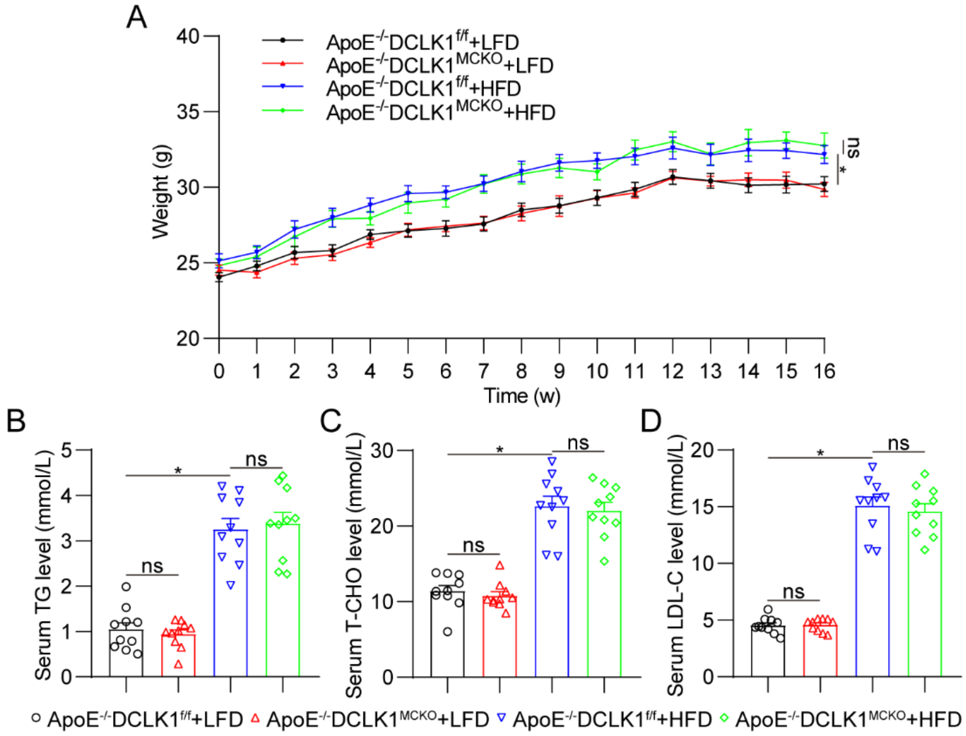

Appendix Figure S2. DCLK1 deletion does not affect serum lipid levels in HFD-fed ApoE$^{-/-}$ mice. (A) Average body weights of mice recoded each week for the duration of the animal experiment (n=10 biological replicates). (B-D) Serum levels of triglycerides (TG) (B), total cholesterol (T-CHO) (C), low-density lipoproteins (LDL-C) (D) were examined using commercial kits (n=10 biological replicates). Data were expressed as mean ± SEM; *$P < 0.05$; ns, not significant, two-tailed unpaired Student's t-test.

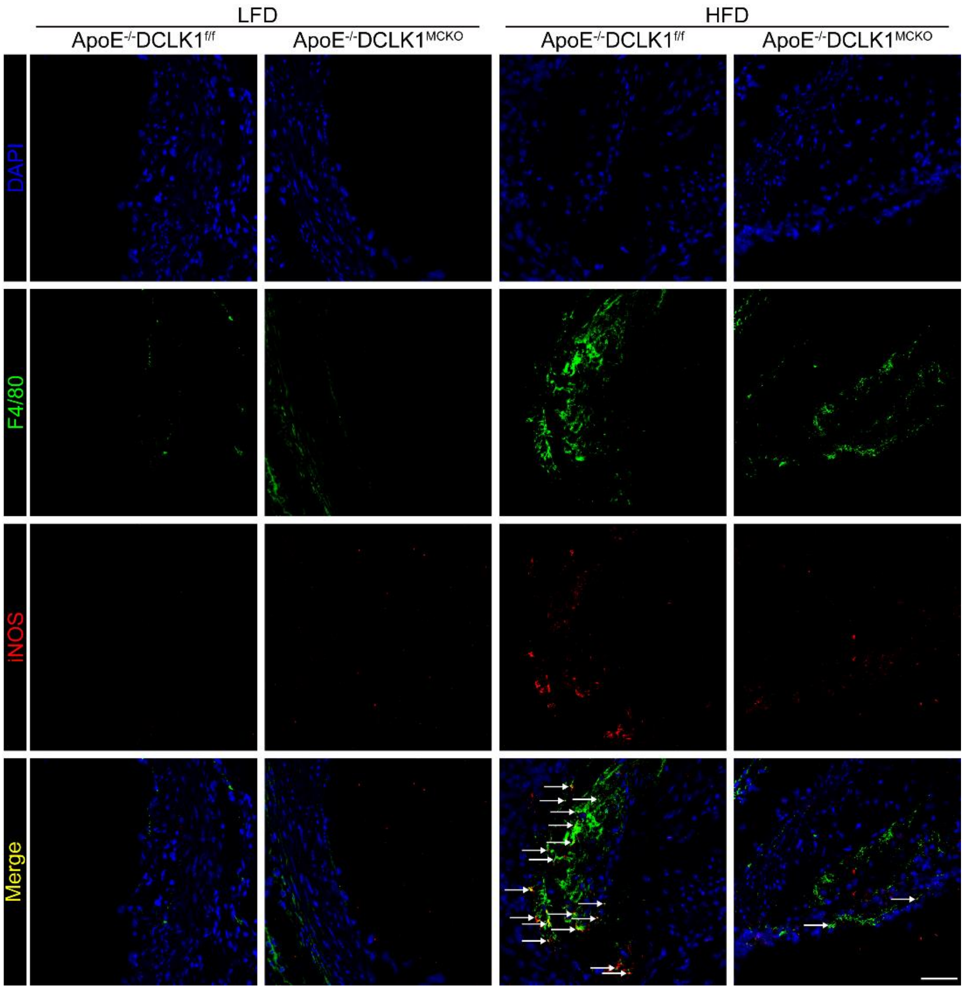

Appendix Figure S3. DCLK1 deletion reduces pro-inflammatory macrophages in aortic roots of HFD-fed ApoE$^{-/-}$ mice. Representative immunofluorescence staining of F4/80 (green) and iNOS (red) in aortic roots (scale bar=25 μm). Tissues were counterstained with DAPI (blue).

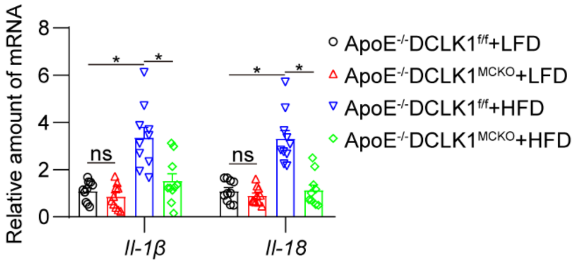

Appendix Figure S4. mRNA levels of inflammatory cytokines *Il-1β* and *Il-18* in aortas. The values of mRNA levels were normalized to *Rn18s* (n=10 biological replicates). Data were expressed as mean ± SEM; *$P < 0.05$; ns, not significant, two-tailed unpaired Student's t-test.

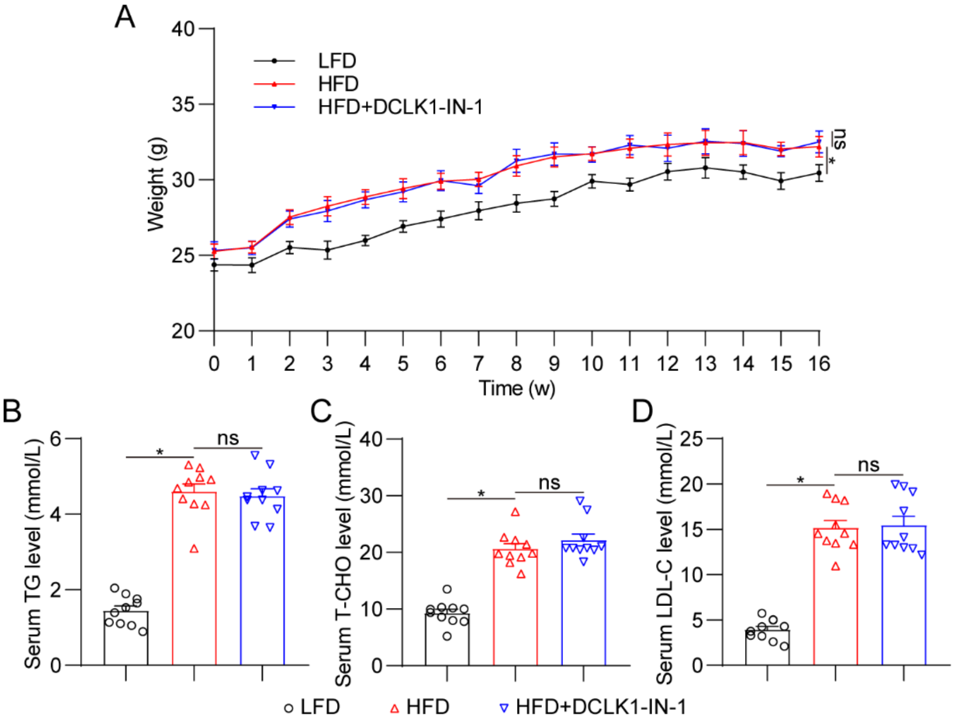

Appendix Figure S5. DCLK1-IN-1 does not affect serum lipid levels in HFD-fed ApoE$^{-/-}$ mice. (A) Average body weights of mice recoded each week for the duration of the animal experiment (n=10 biological replicates). (B-D) Serum levels of triglycerides (TG) (B), total cholesterol (T-CHO) (C), low-density lipoproteins (LDL-C) (D) were examined using commercial kits (n=10 biological replicates). Data were expressed as mean ± SEM; *$P < 0.05$; ns, not significant, two-tailed unpaired Student’s t-test.

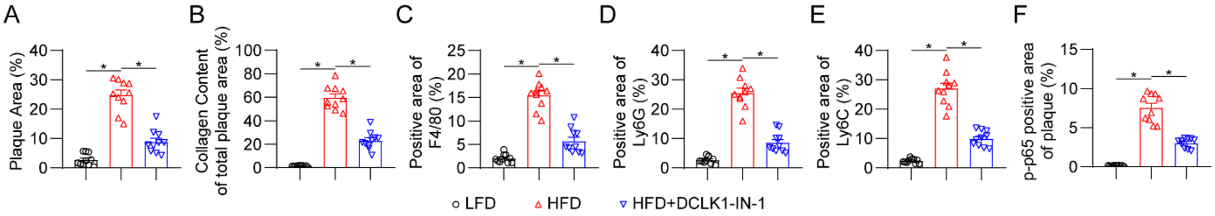

Appendix Figure S6. Quantification for Figure 7E-J. (A-F) Quantification for Figure 7E-J, respectively (n=10 biological replicates). Data were expressed as mean ± SEM; *P < 0.05, two-tailed unpaired Student's t-test.
