# Supplementary figures and images for: Macrophage DCLK1 promotes atherosclerosis via binding to IKKβ and inducing inflammatory responses
Source: EMBO Mol Med. 2023 Mar 10;15(5):e17198. doi: 10.15252/emmm.202217198 (PMC10165355; doi:10.15252/emmm.202217198)

Appendix Figure 1C

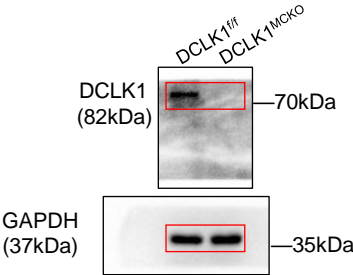

Supplement: Supplementary file 2 — Source Data for Appendix [file EMMM-15-e17198-s004.zip › Source Data for Appendix figures/Figure S1/1C/western blot.pdf]

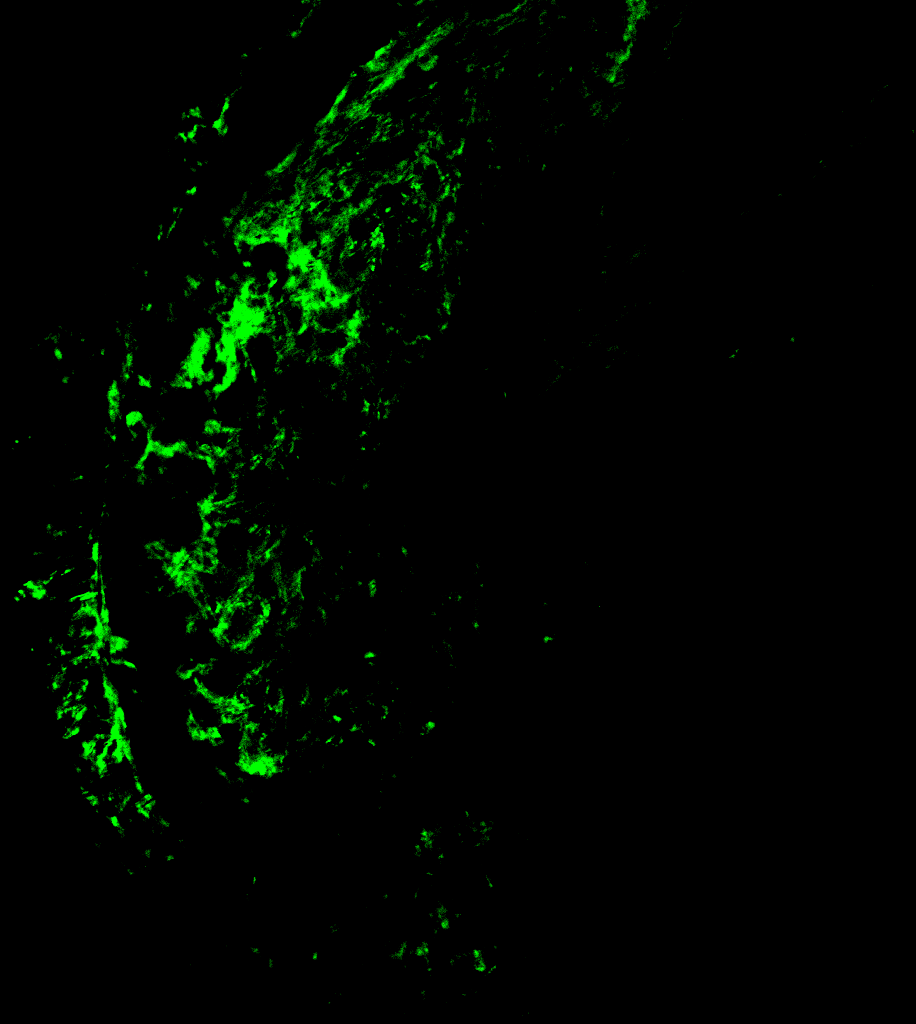



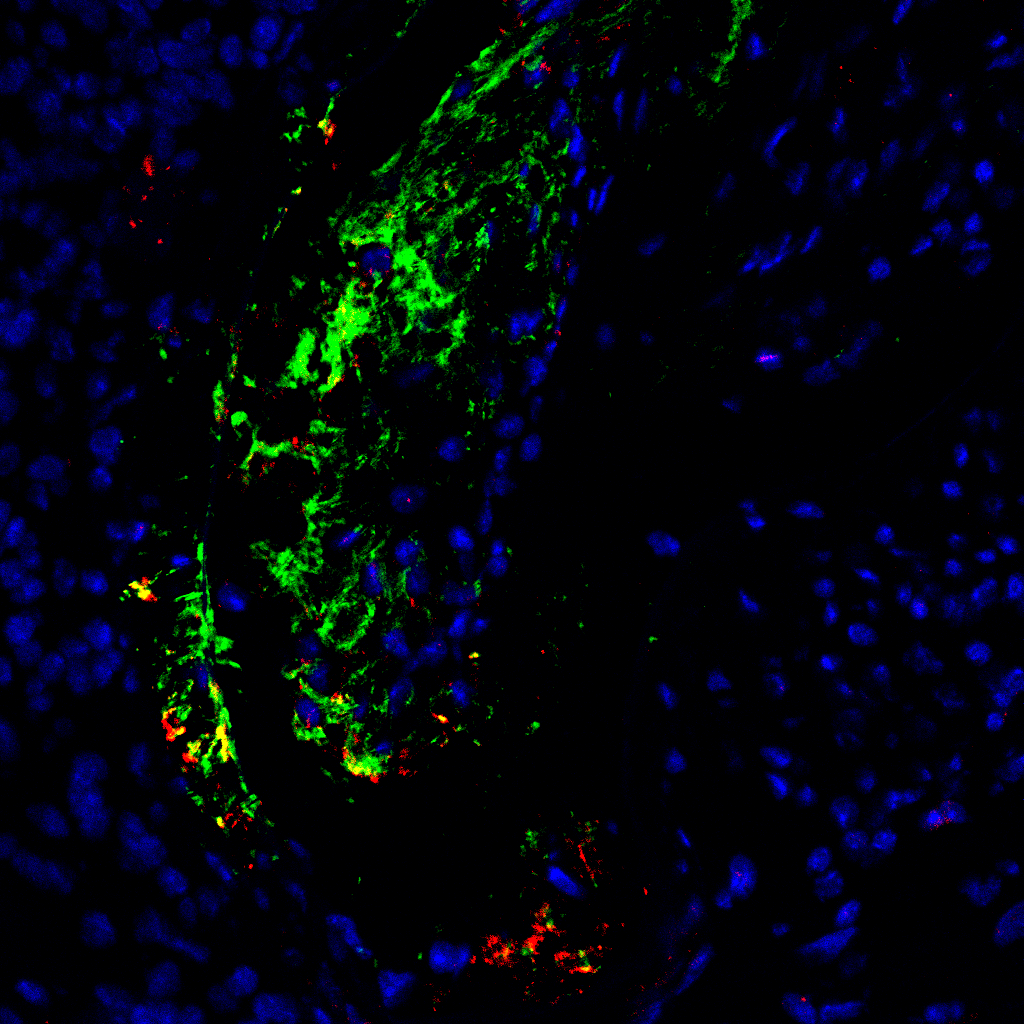

Supplement: Supplementary file 2 — Source Data for Appendix [file EMMM-15-e17198-s004.zip › Source Data for Appendix figures/Figure S3/HFD-DCLK1ff.pdf]

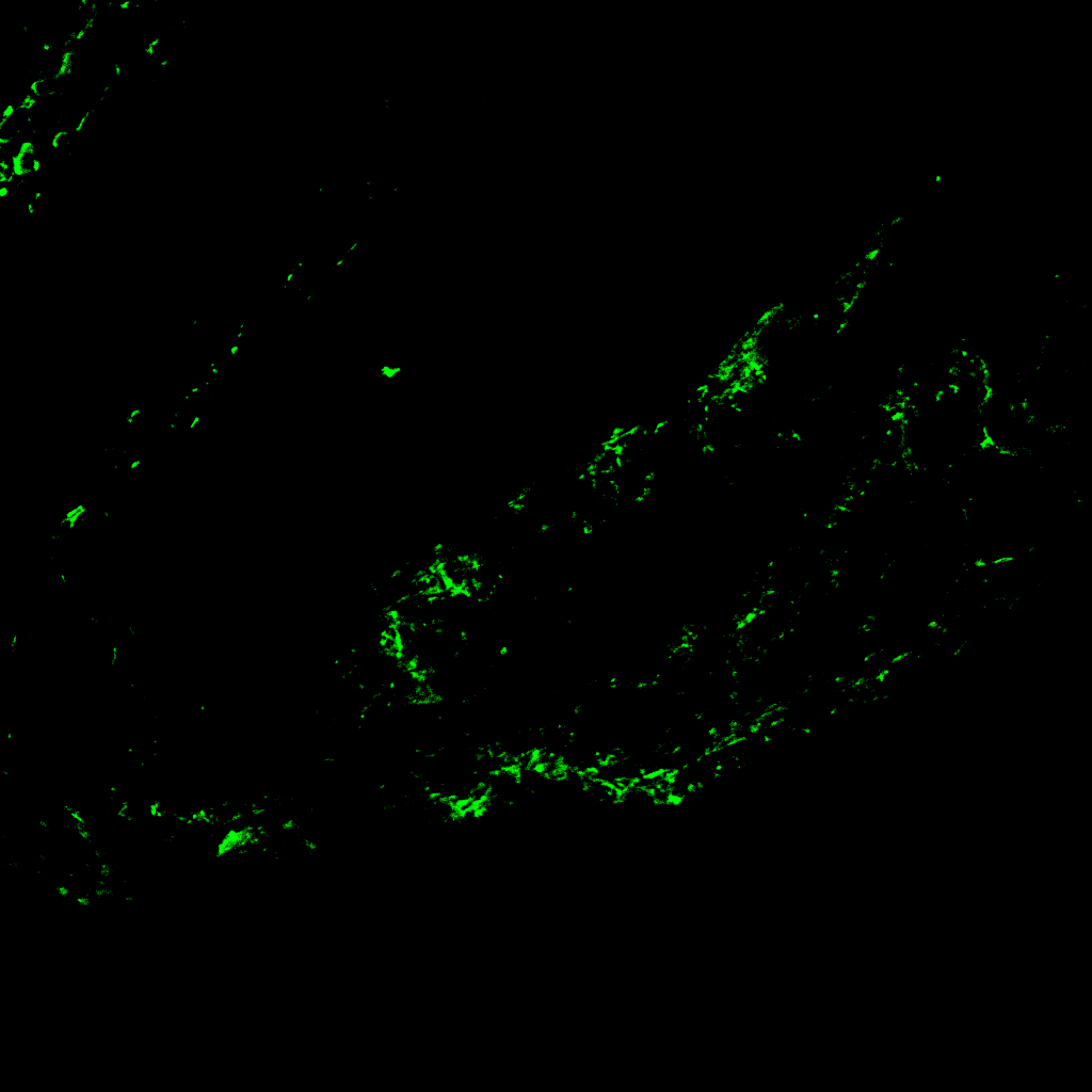



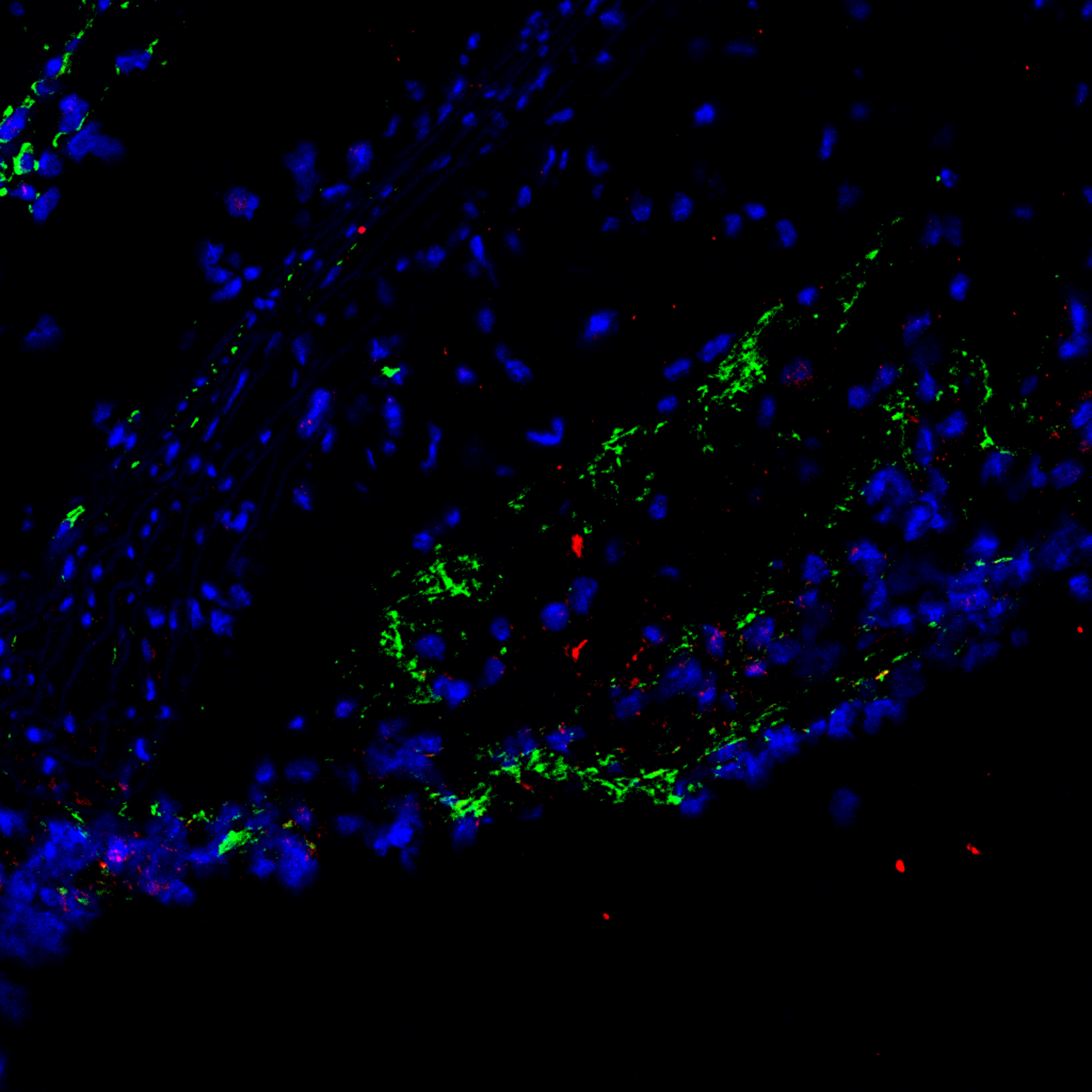

Supplement: Supplementary file 2 — Source Data for Appendix [file EMMM-15-e17198-s004.zip › Source Data for Appendix figures/Figure S3/HFD-DCLK1MCKO.pdf]

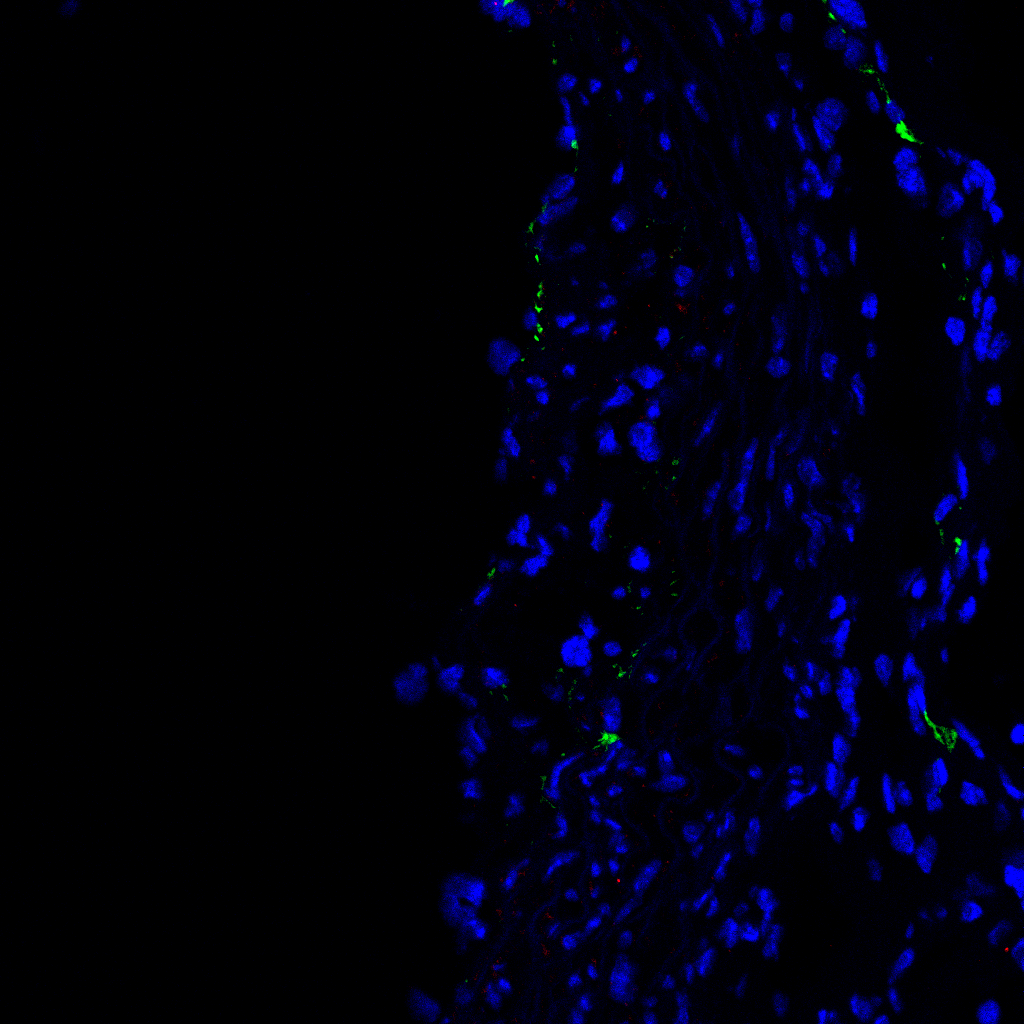

Supplement: Supplementary file 2 — Source Data for Appendix [file EMMM-15-e17198-s004.zip › Source Data for Appendix figures/Figure S3/LFD-DCLK1ff.pdf]

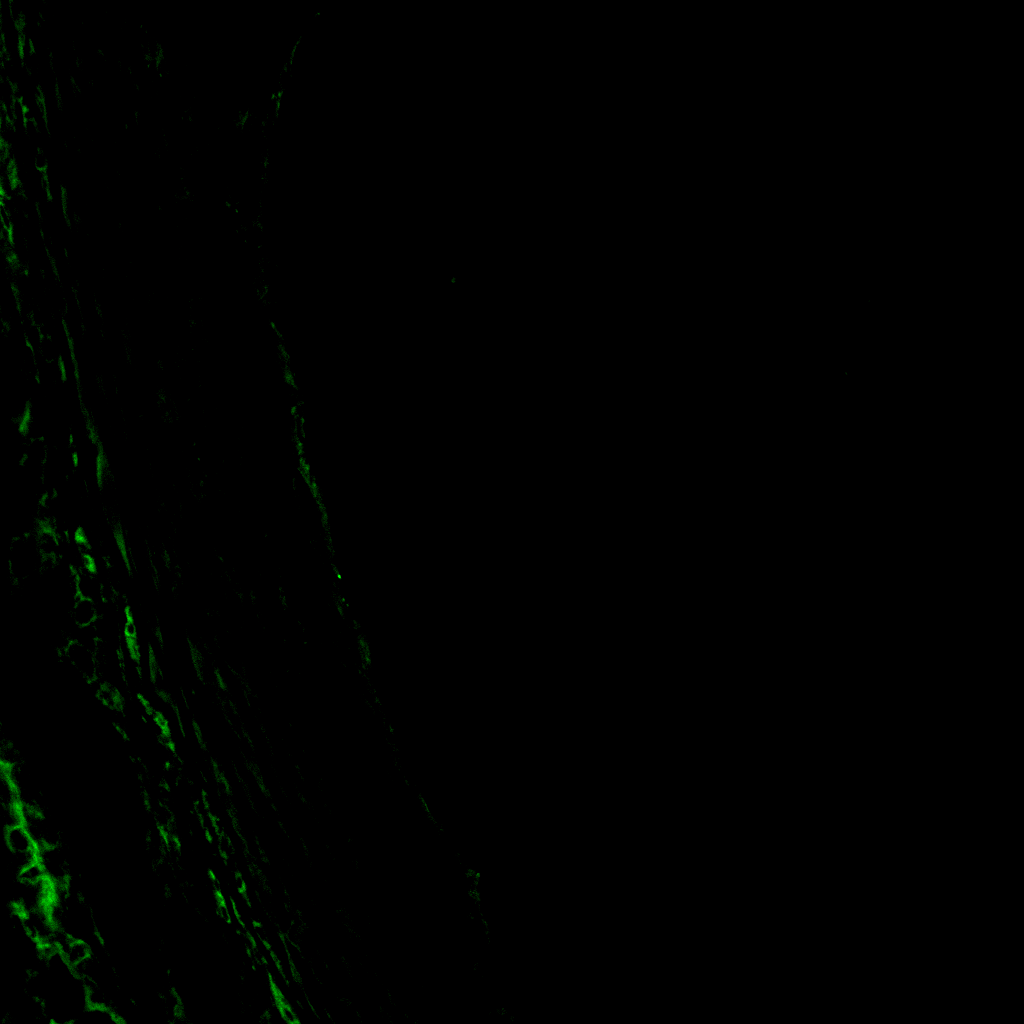



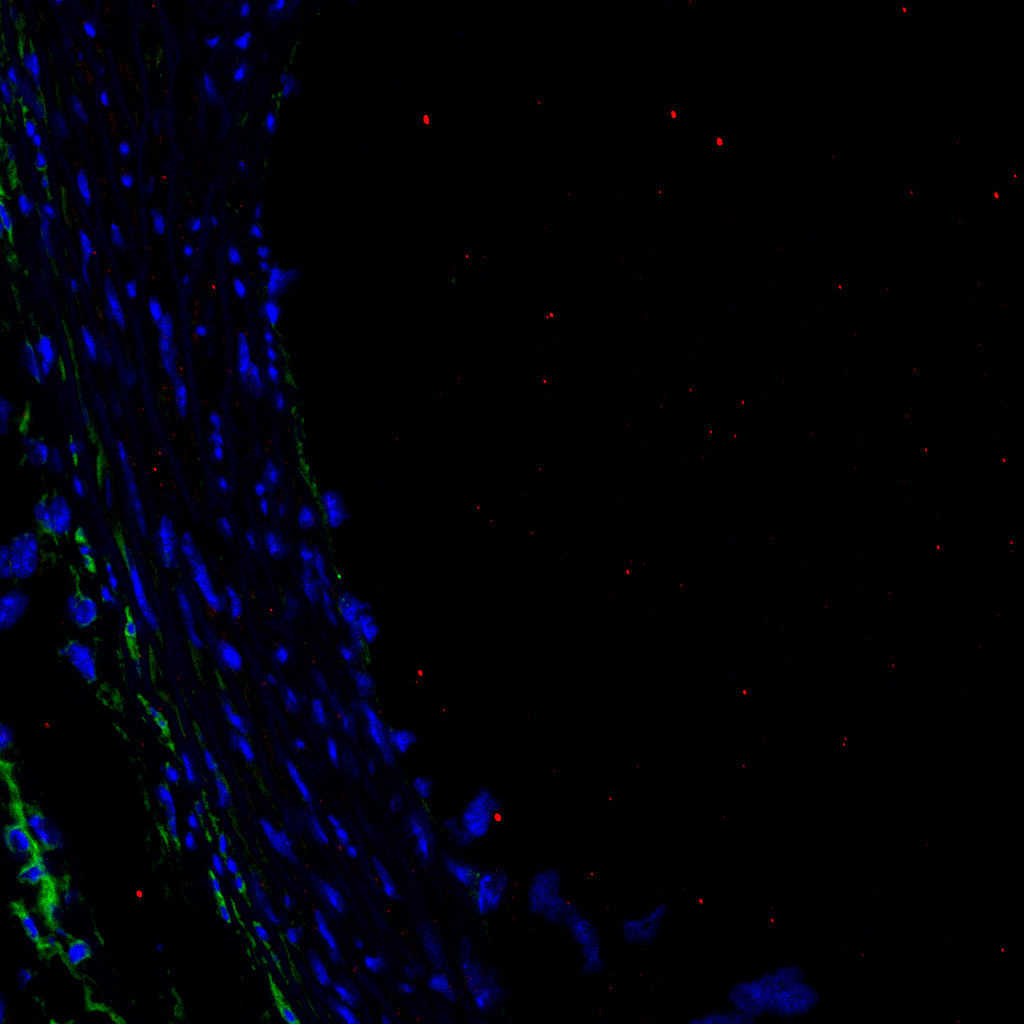

Supplement: Supplementary file 2 — Source Data for Appendix [file EMMM-15-e17198-s004.zip › Source Data for Appendix figures/Figure S3/LFD-DCLK1MCKO.pdf]

Figure 1B

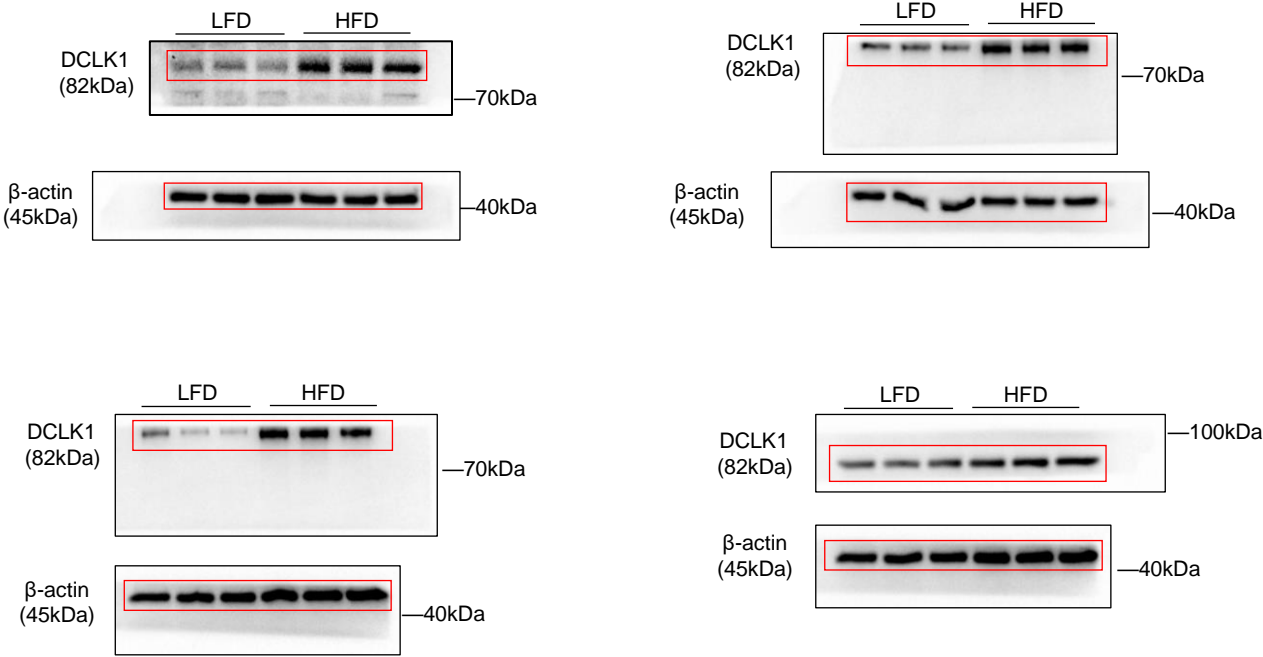

Supplement: Supplementary file 3 — Source Data for Figure 1 [file EMMM-15-e17198-s006.zip › EMM-2022-17198-V2-Figure_1_Source_Data-sd/1B-C/1B-western blot (including quantification).pdf]

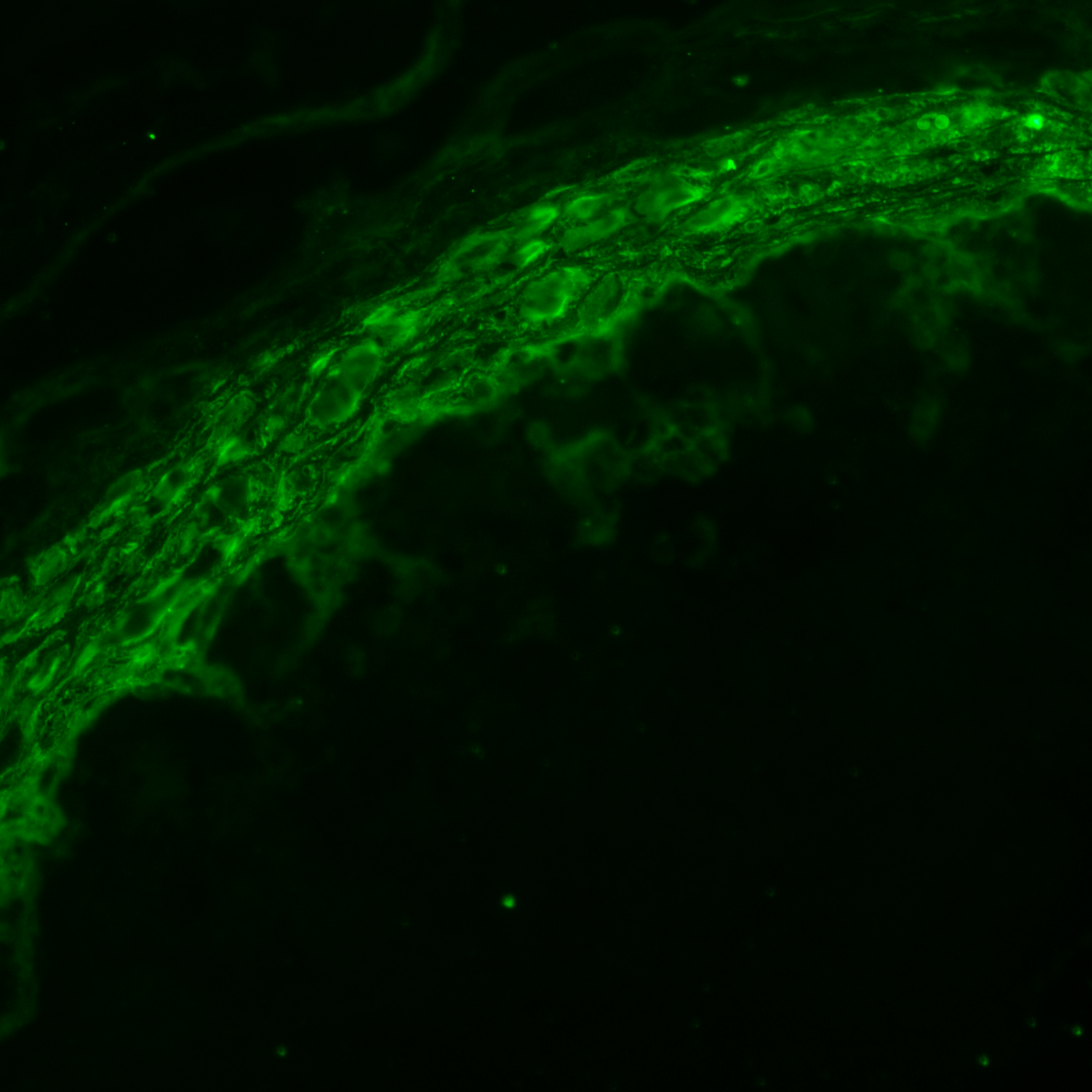



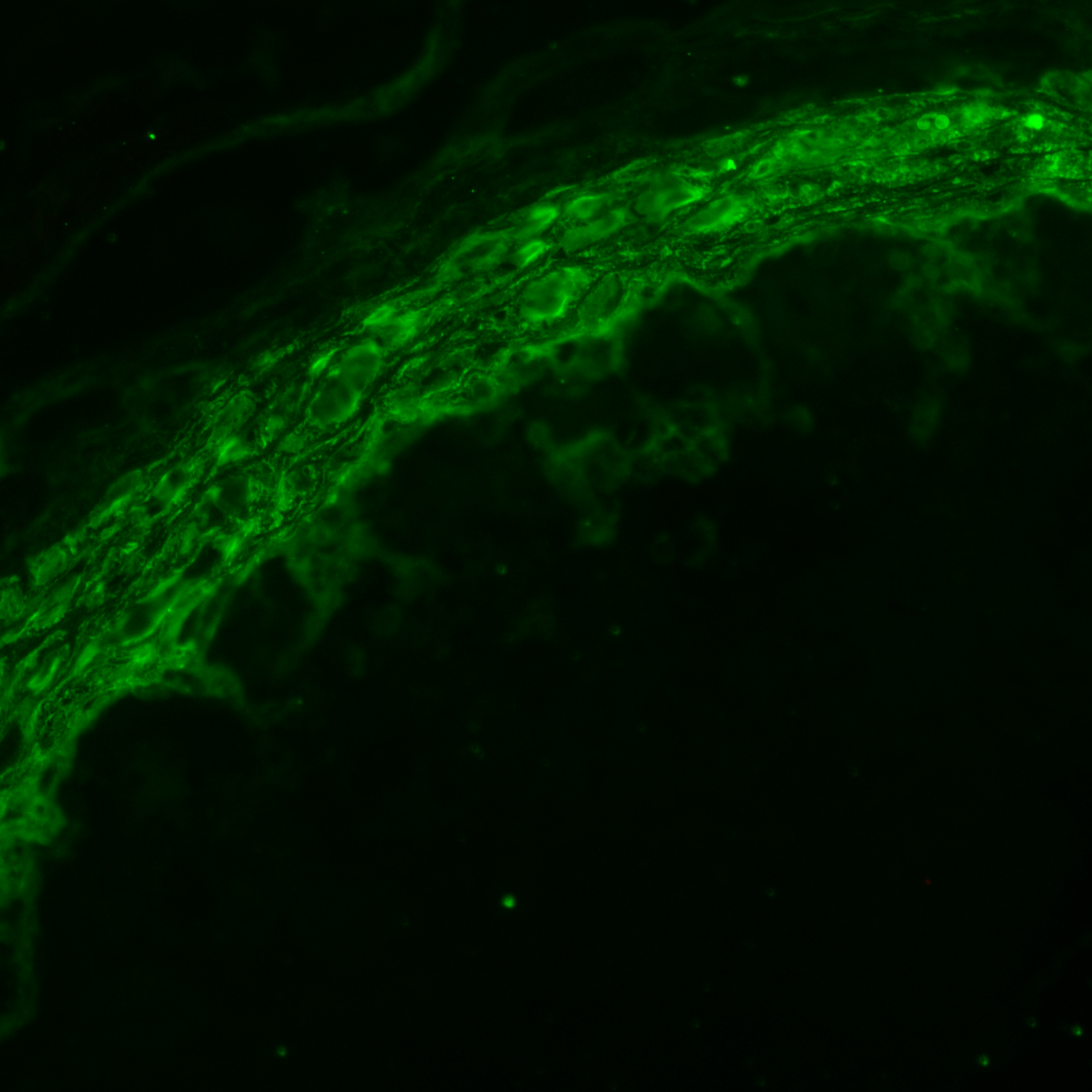

Supplement: Supplementary file 3 — Source Data for Figure 1 [file EMMM-15-e17198-s006.zip › EMM-2022-17198-V2-Figure_1_Source_Data-sd/1F/LFD-╬▒-SMA; DCLK1; Merge.pdf]

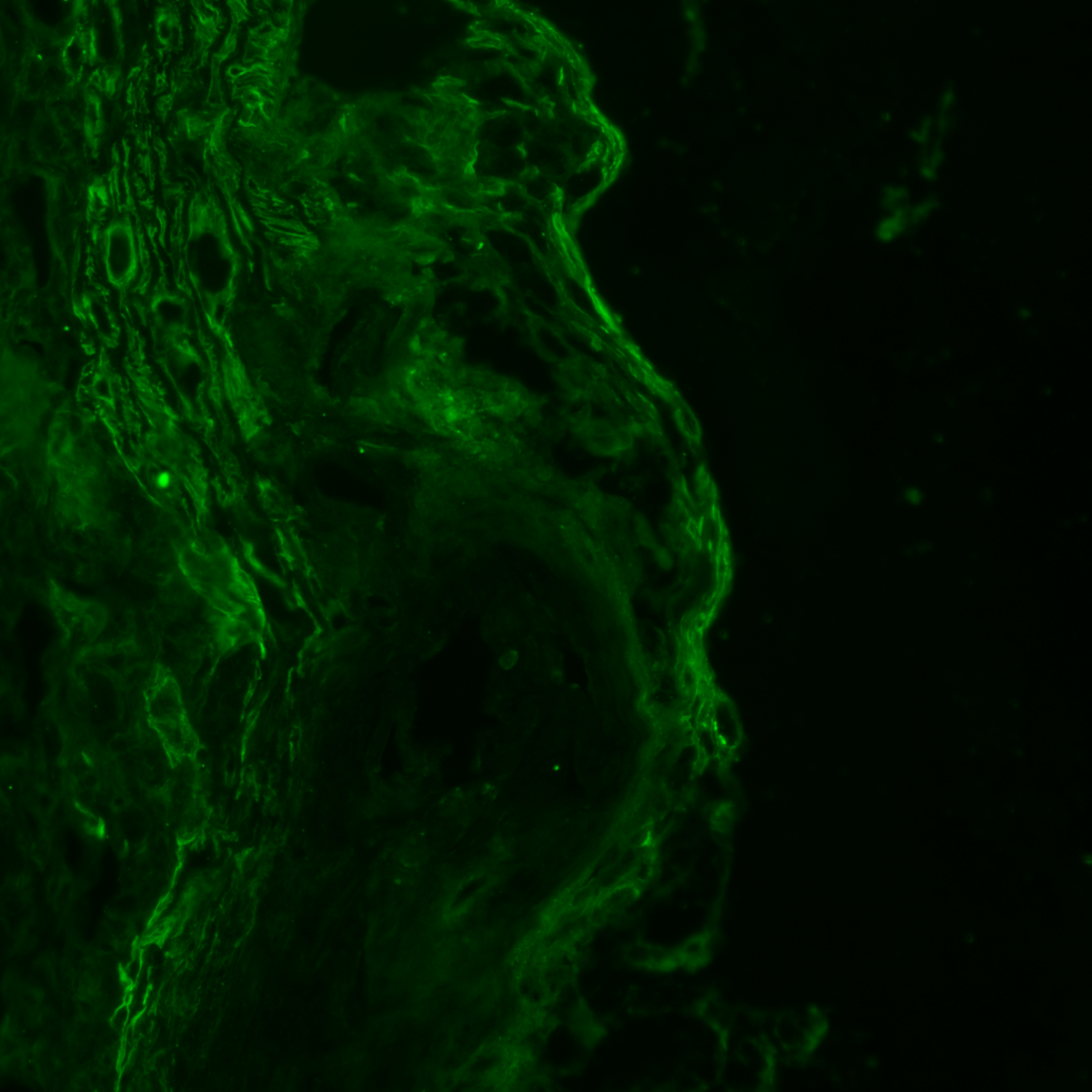



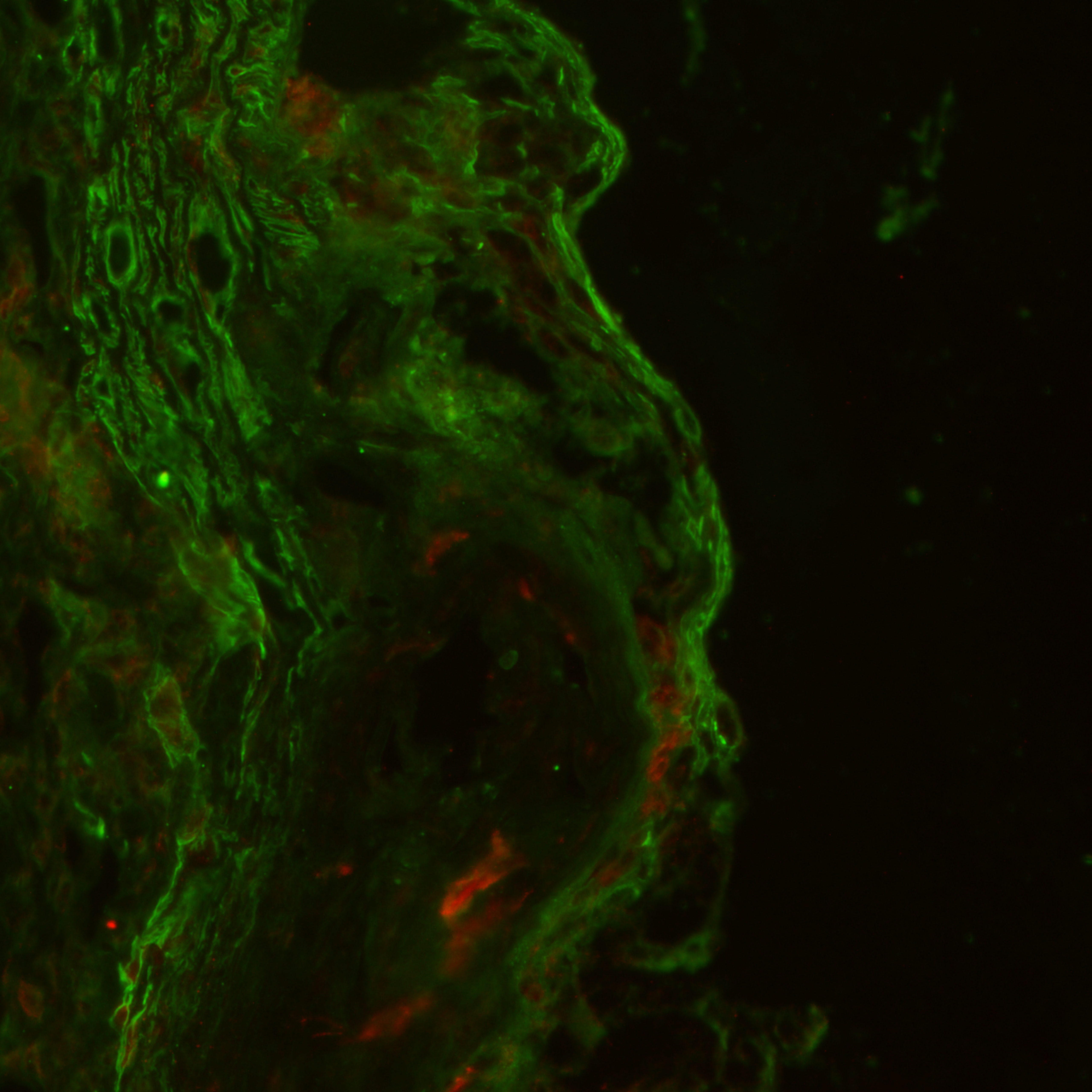

Supplement: Supplementary file 3 — Source Data for Figure 1 [file EMMM-15-e17198-s006.zip › EMM-2022-17198-V2-Figure_1_Source_Data-sd/1F/HFD-╬▒-SMA; DCLK1; Merge.pdf]

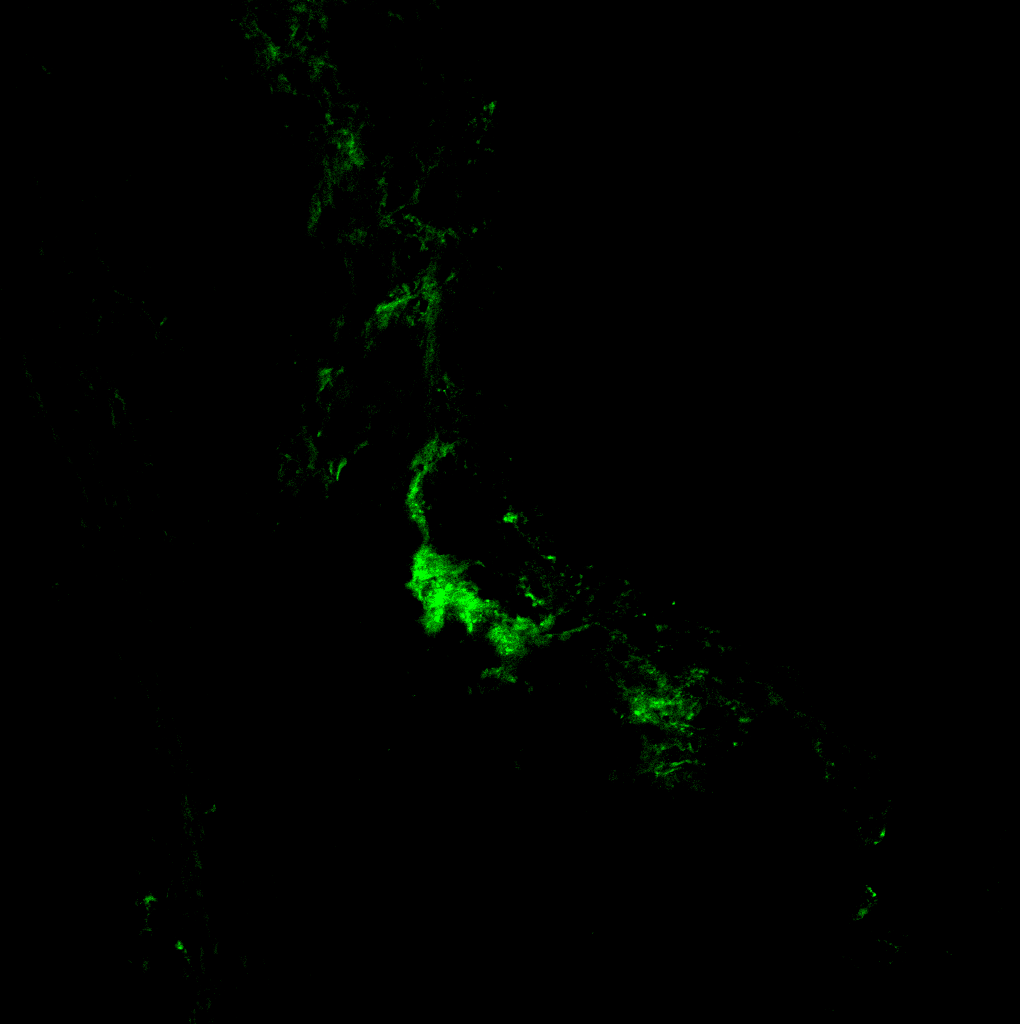



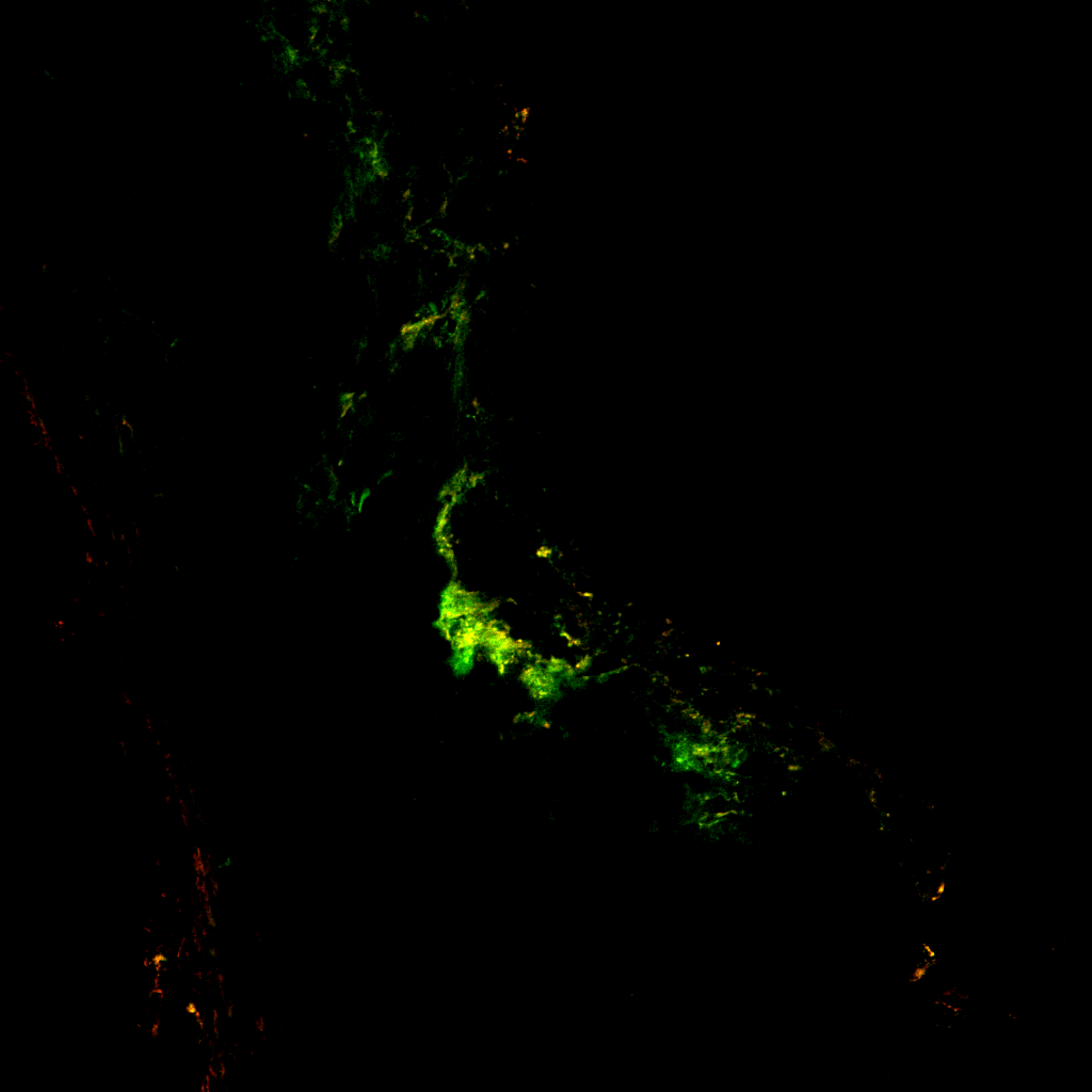

Supplement: Supplementary file 3 — Source Data for Figure 1 [file EMMM-15-e17198-s006.zip › EMM-2022-17198-V2-Figure_1_Source_Data-sd/1G/HFD-F480; DCLK1; Merge.pdf]

Figure 1H

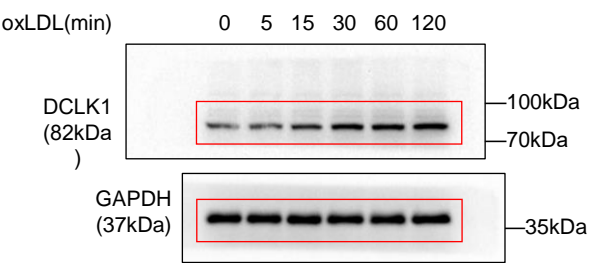

Supplement: Supplementary file 3 — Source Data for Figure 1 [file EMMM-15-e17198-s006.zip › EMM-2022-17198-V2-Figure_1_Source_Data-sd/1H-I/1H-western blot.pdf]

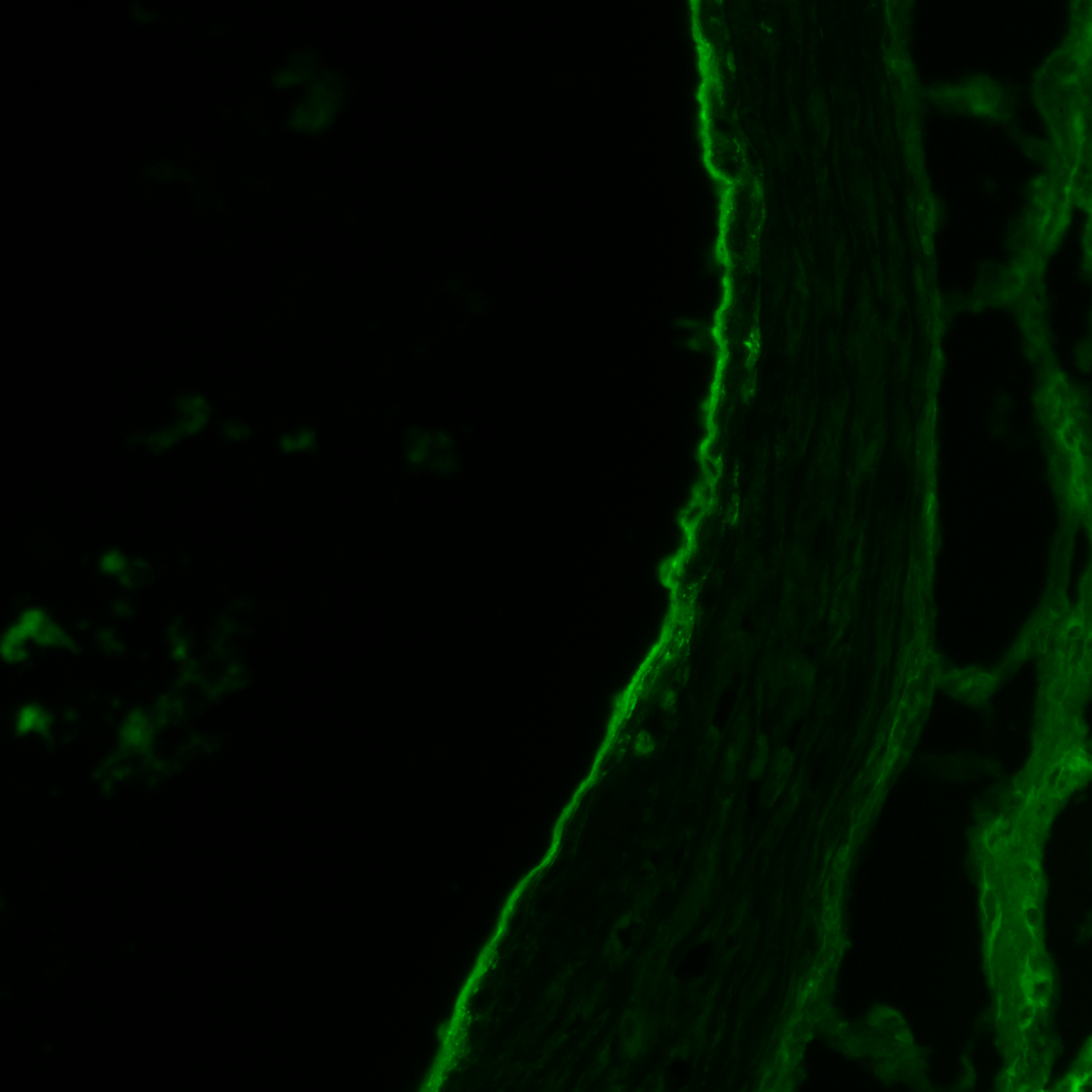



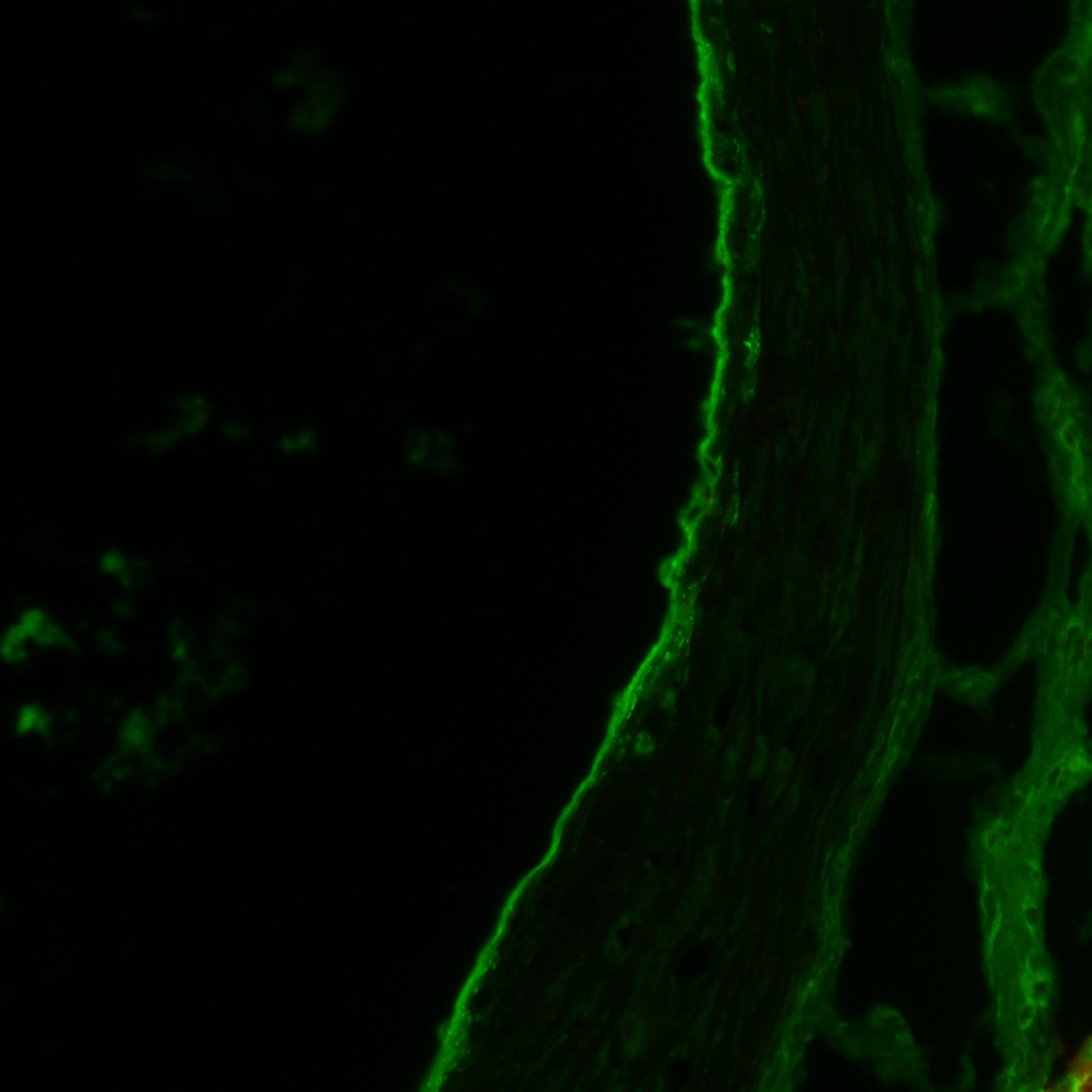

Supplement: Supplementary file 3 — Source Data for Figure 1 [file EMMM-15-e17198-s006.zip › EMM-2022-17198-V2-Figure_1_Source_Data-sd/1E/LFD-CD31; DCLK1; Merge.pdf]

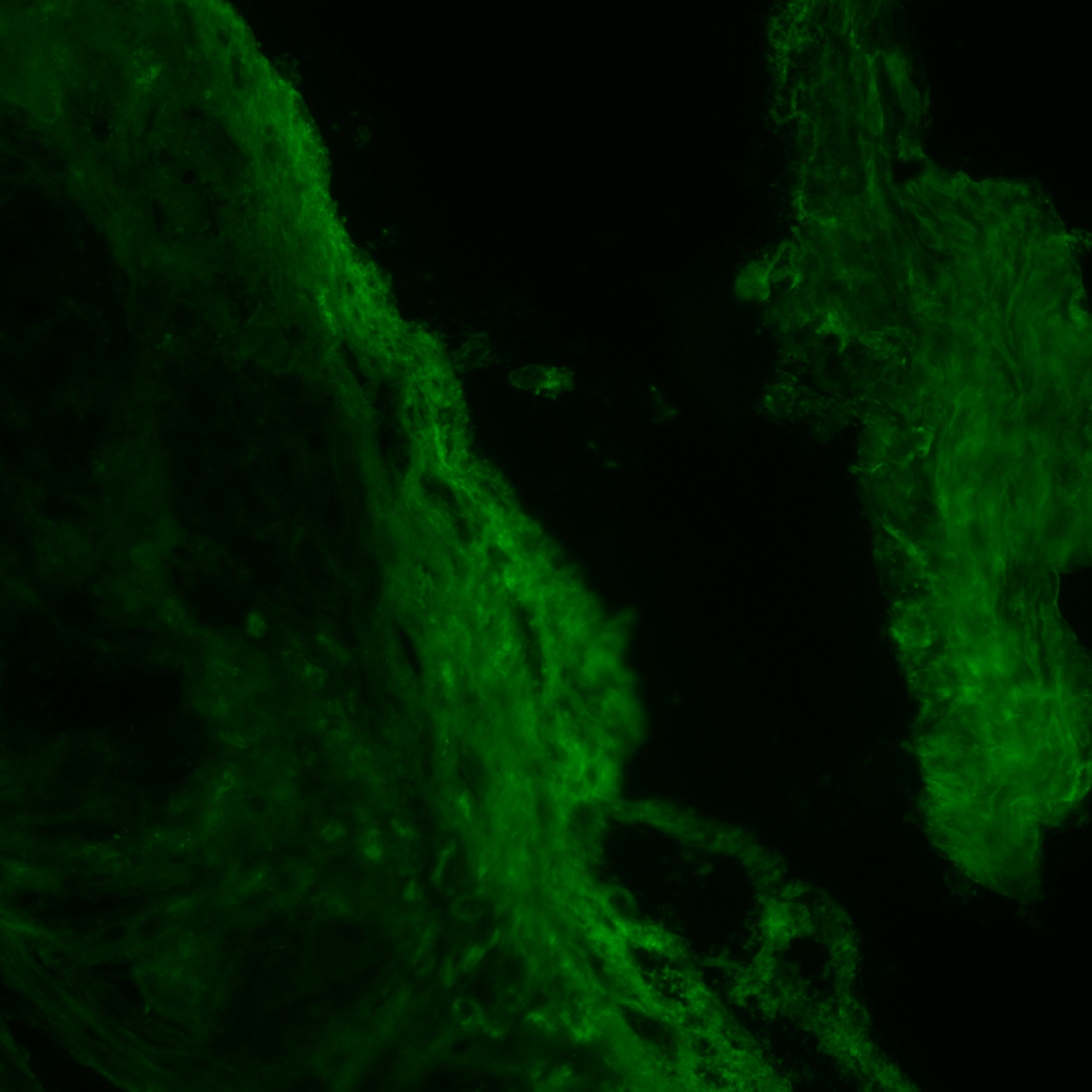



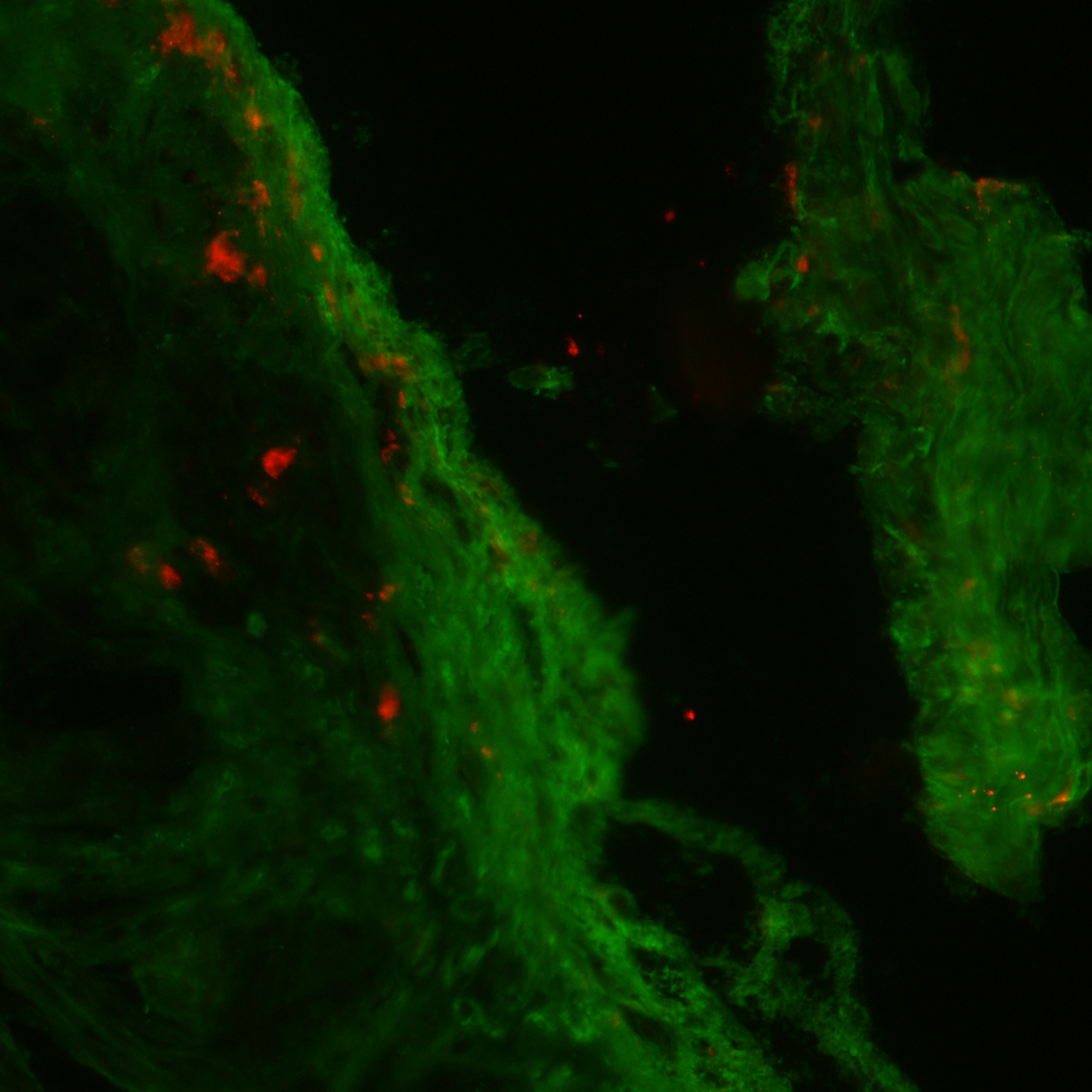

Supplement: Supplementary file 3 — Source Data for Figure 1 [file EMMM-15-e17198-s006.zip › EMM-2022-17198-V2-Figure_1_Source_Data-sd/1E/HFD-CD31; DCLK1; Merge.pdf]

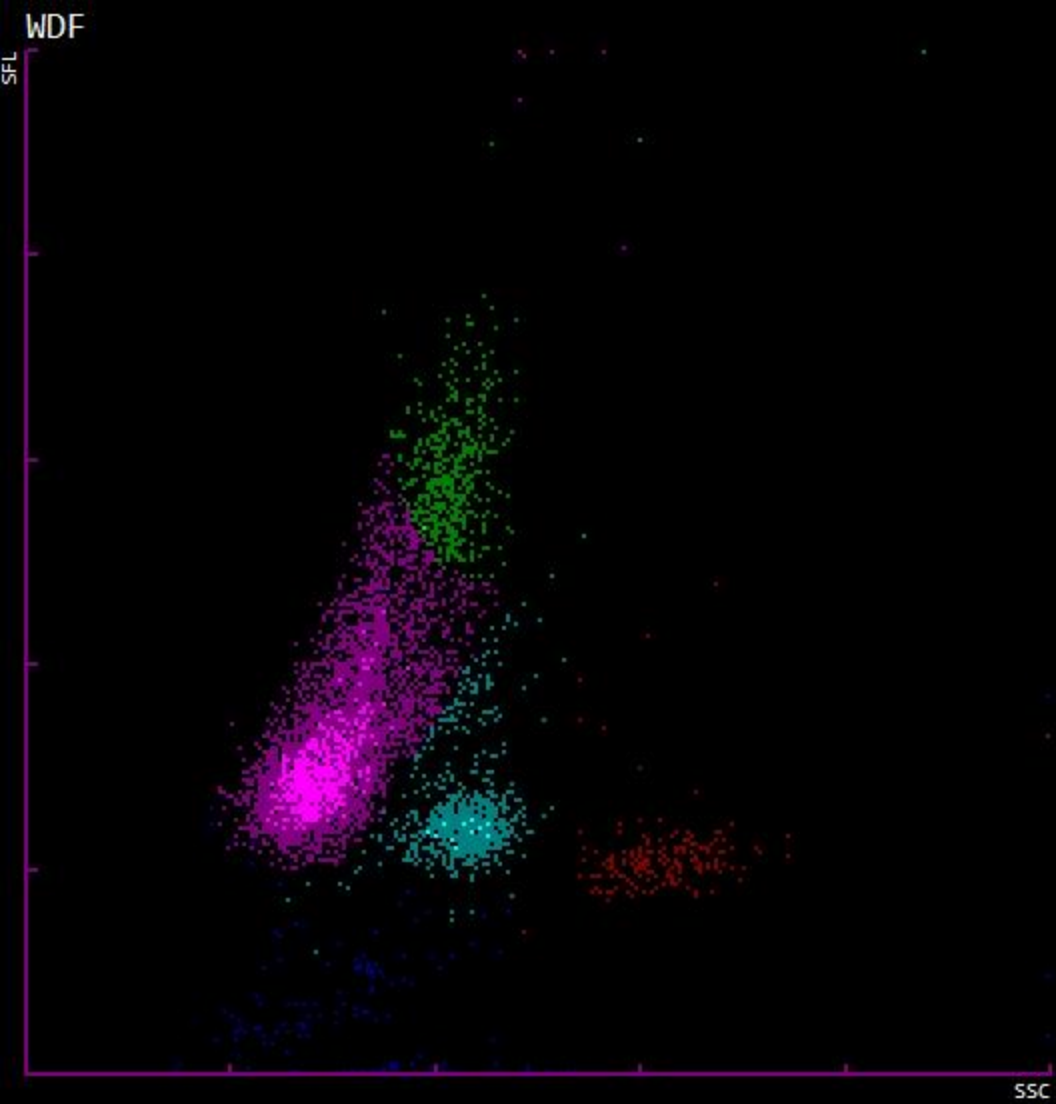

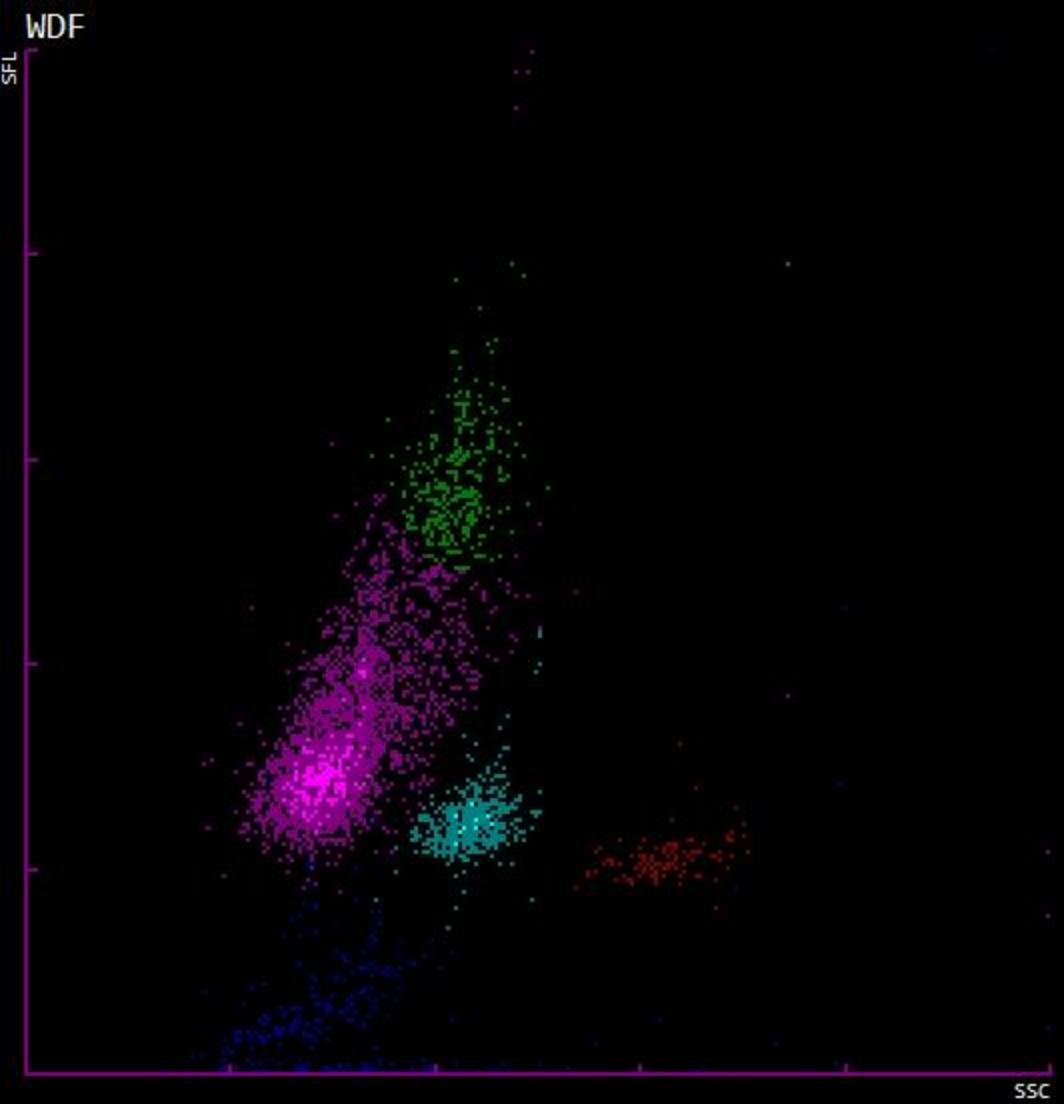

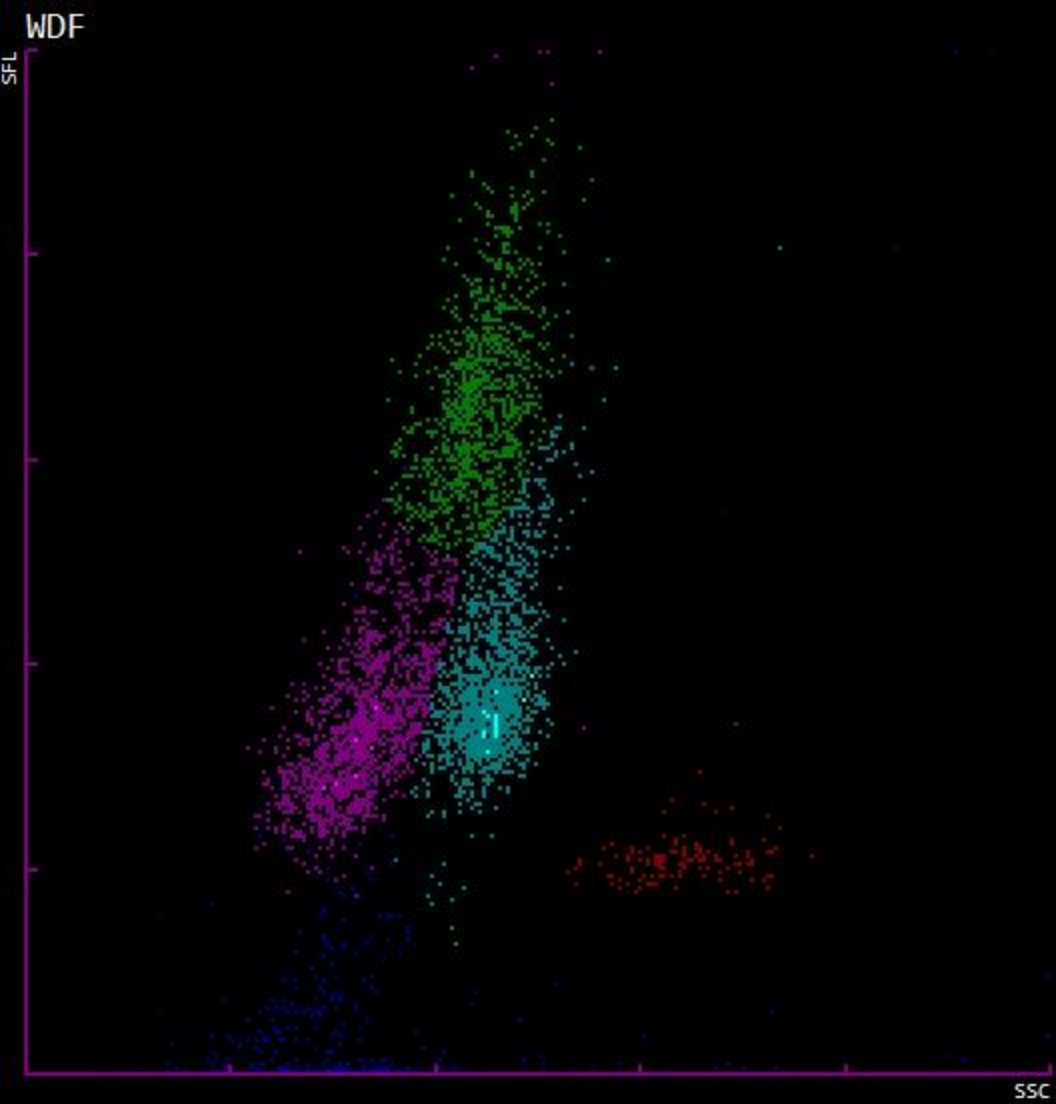

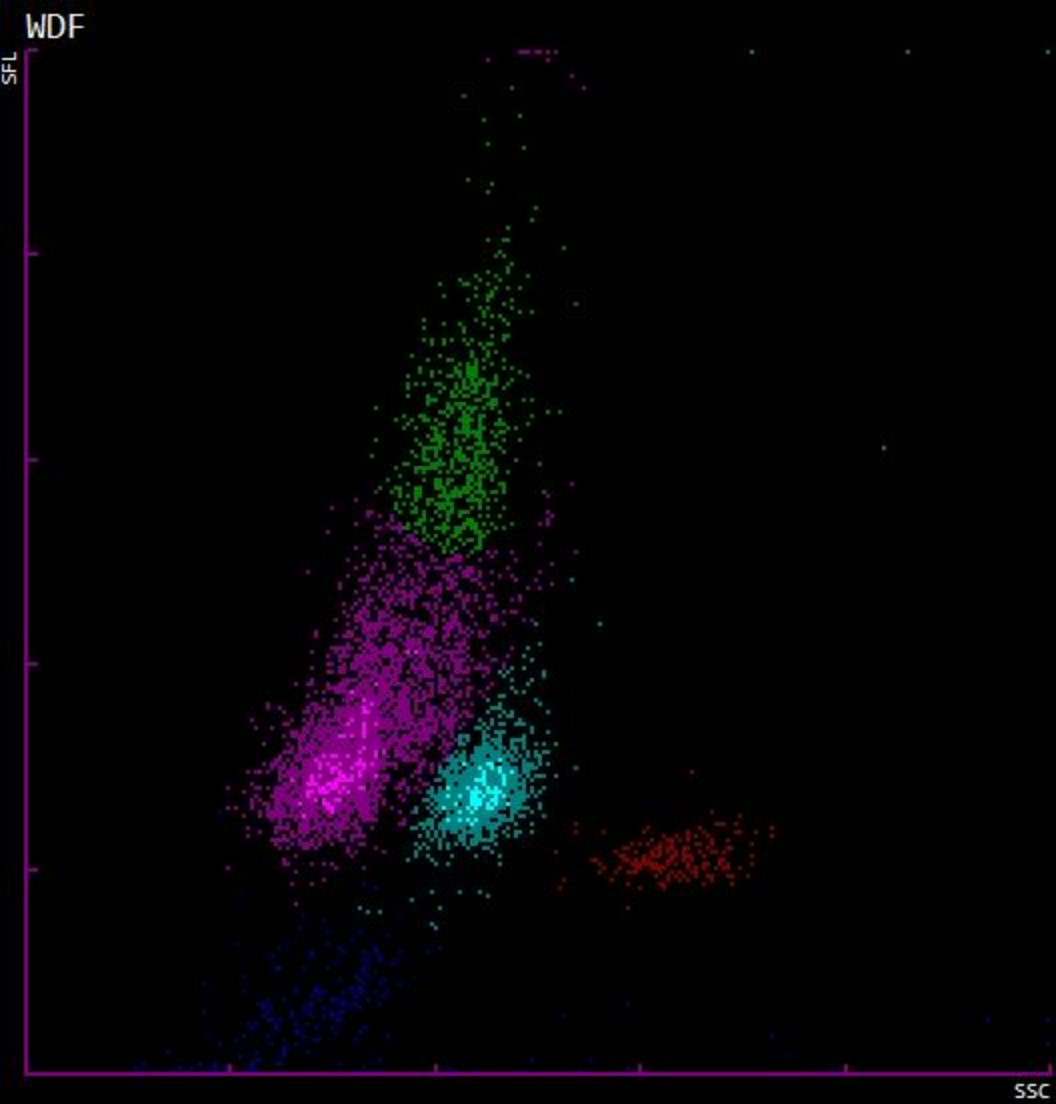

Supplement: Supplementary file 5 — Source Data for Figure 3 [file EMMM-15-e17198-s009.zip › EMM-2022-17198-V2-Figure_3_Source_Data-sd/3G-I/3G-Scatter diagram.pdf]

Figure 4L

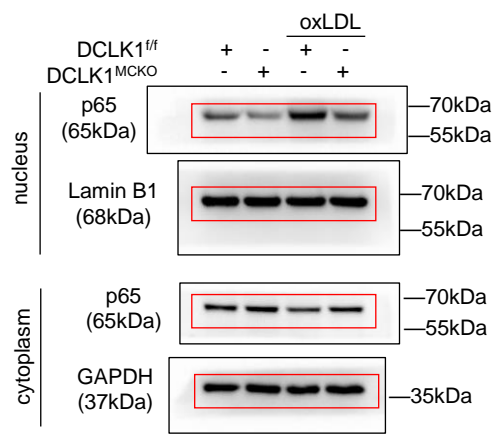

Supplement: Supplementary file 6 — Source Data for Figure 4 [file EMMM-15-e17198-s010.zip › EMM-2022-17198-V2-Figure_4_Source_Data-sd/4L-M/4L-western blot.pdf]

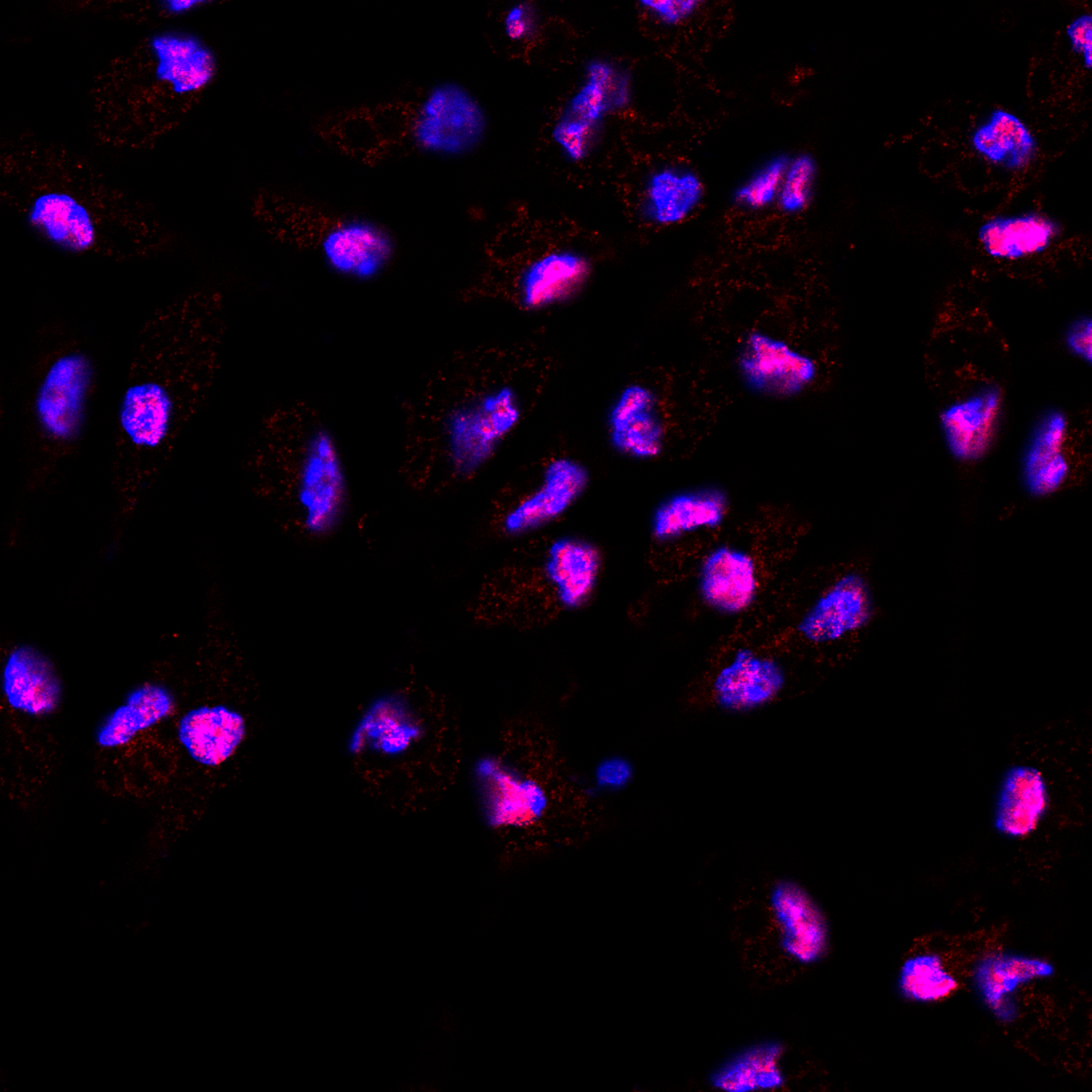

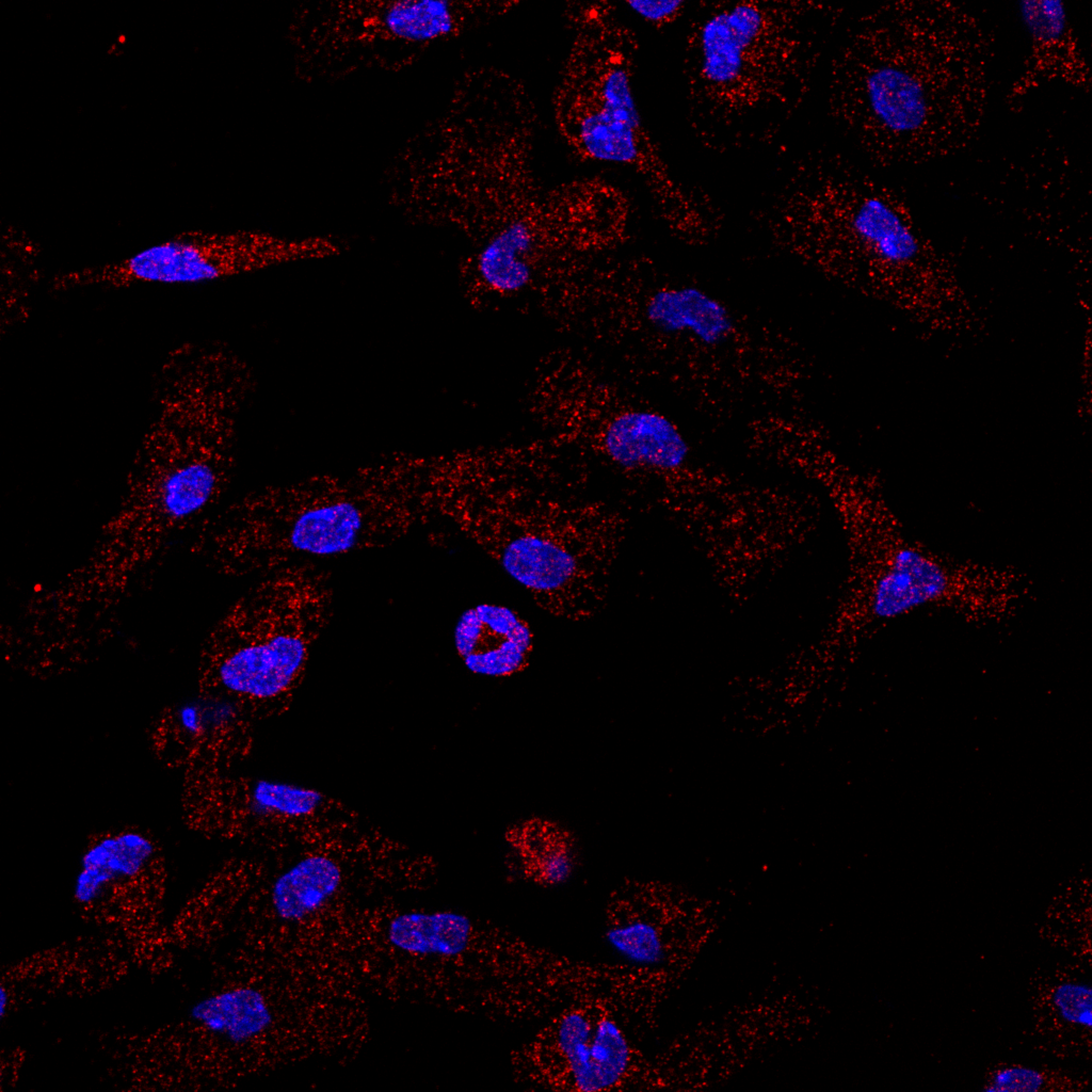

Supplement: Supplementary file 6 — Source Data for Figure 4 [file EMMM-15-e17198-s010.zip › EMM-2022-17198-V2-Figure_4_Source_Data-sd/4N-O/4N-p65 staining.pdf]

Figure 4J

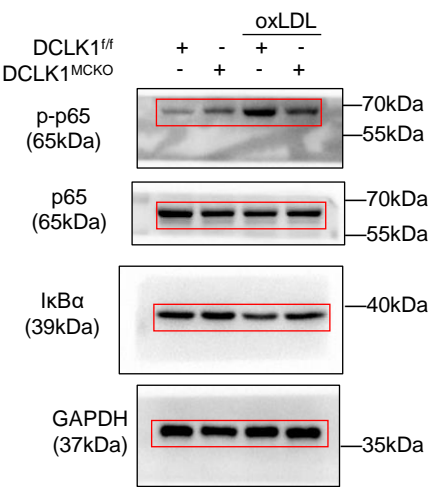

Supplement: Supplementary file 6 — Source Data for Figure 4 [file EMMM-15-e17198-s010.zip › EMM-2022-17198-V2-Figure_4_Source_Data-sd/4J-K/4J-western blot.pdf]

Figure 5B

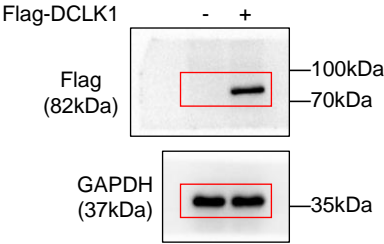

Supplement: Supplementary file 7 — Source Data for Figure 5 [file EMMM-15-e17198-s001.zip › Figure 5/5B/western blot.pdf]

Figure 5D

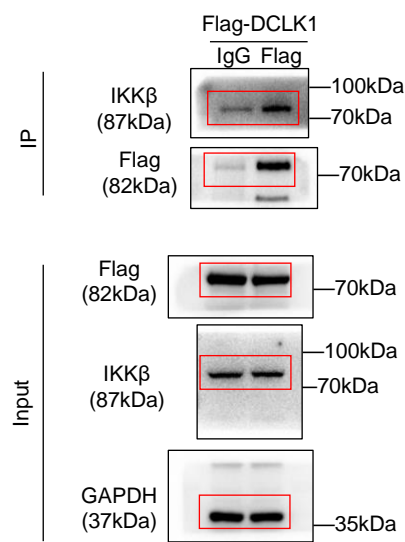

Supplement: Supplementary file 7 — Source Data for Figure 5 [file EMMM-15-e17198-s001.zip › Figure 5/5D/western blot.pdf]

Figure 5E

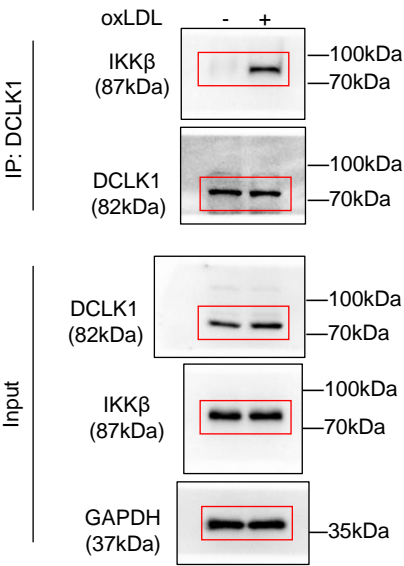

Supplement: Supplementary file 7 — Source Data for Figure 5 [file EMMM-15-e17198-s001.zip › Figure 5/5E/western blot.pdf]

Figure 5F

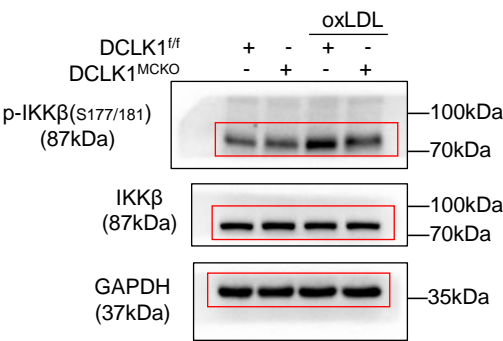

Supplement: Supplementary file 7 — Source Data for Figure 5 [file EMMM-15-e17198-s001.zip › Figure 5/5F/western blot.pdf]

Figure 5G

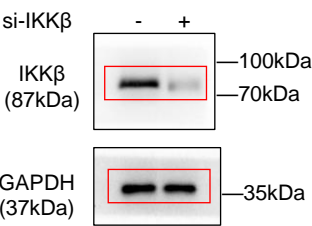

Supplement: Supplementary file 7 — Source Data for Figure 5 [file EMMM-15-e17198-s001.zip › Figure 5/5G/western blot.pdf]

Figure 5H

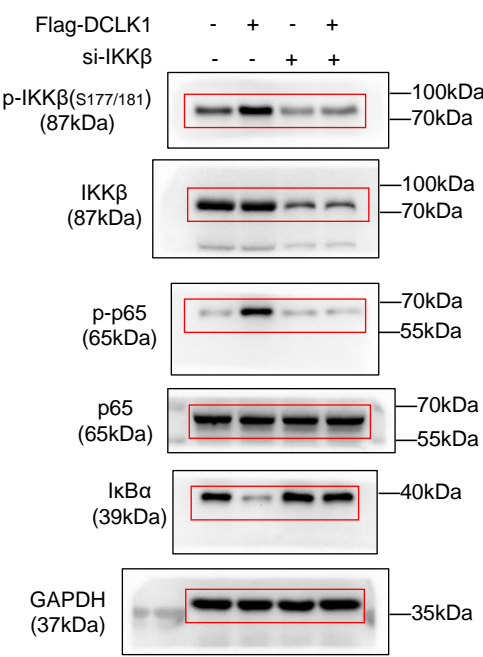

Supplement: Supplementary file 7 — Source Data for Figure 5 [file EMMM-15-e17198-s001.zip › Figure 5/5H-I/5H-western blot.pdf]

Figure 6D

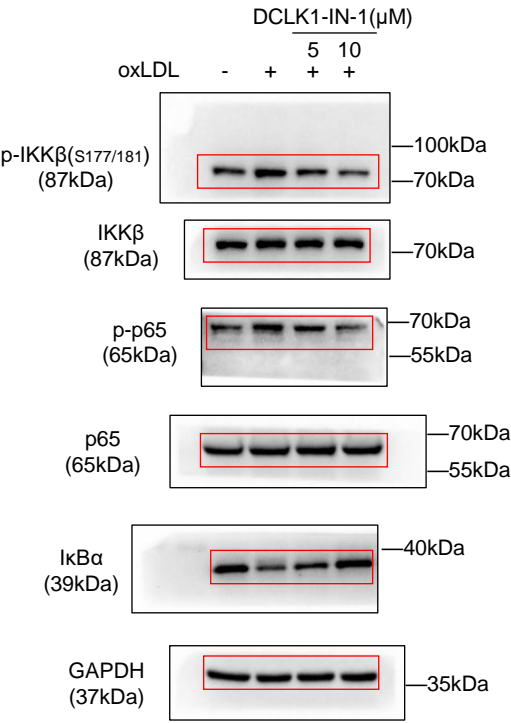

Supplement: Supplementary file 8 — Source Data for Figure 6 [file EMMM-15-e17198-s005.zip › Figure 6/6D-E/6D-western blot.pdf]

Figure 6F

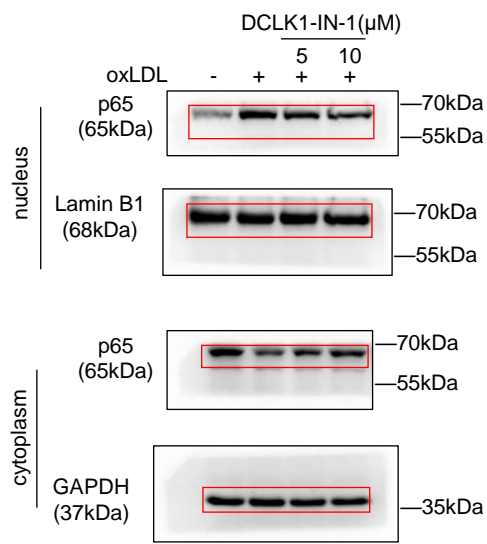

Supplement: Supplementary file 8 — Source Data for Figure 6 [file EMMM-15-e17198-s005.zip › Figure 6/6F-G/6F-western blot.pdf]

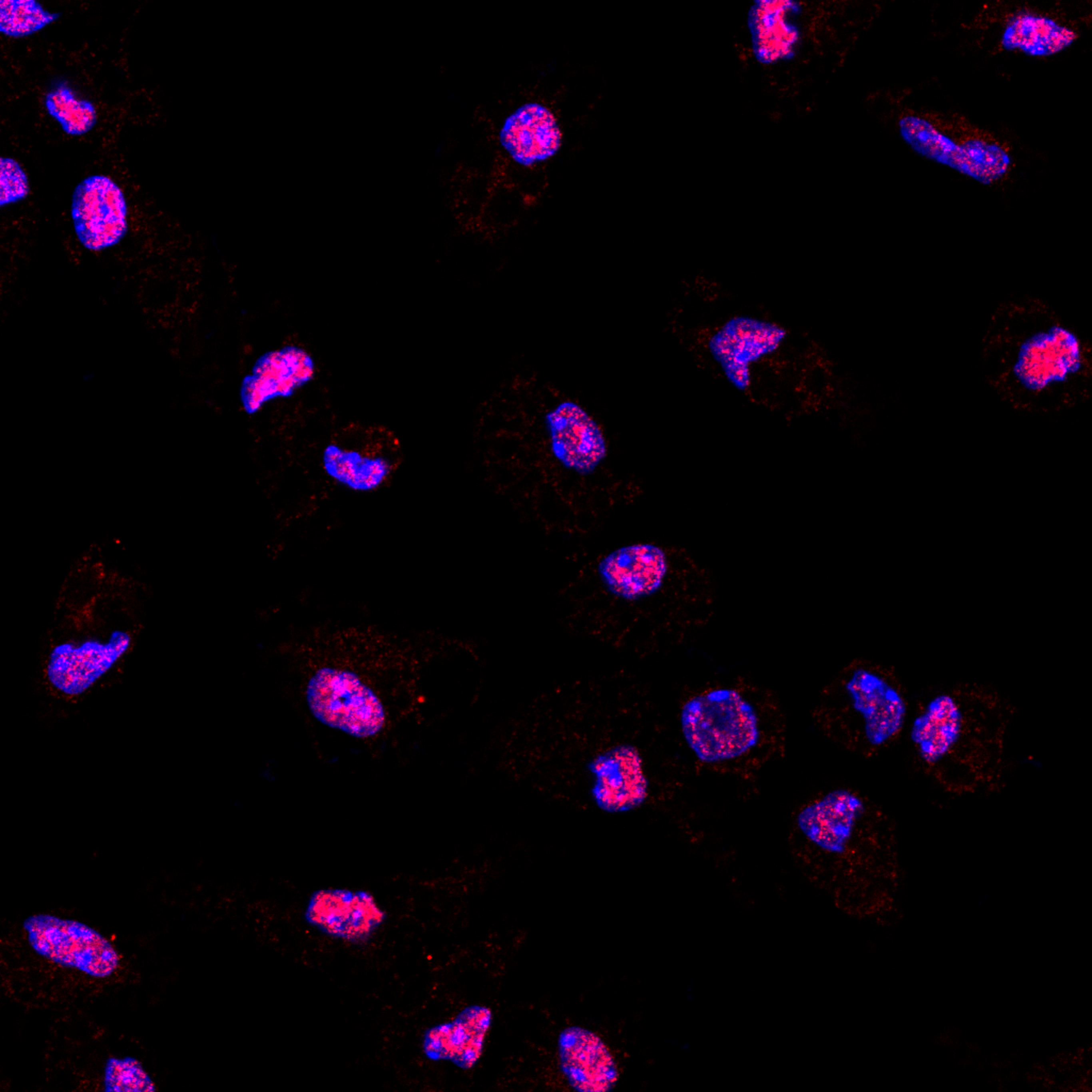

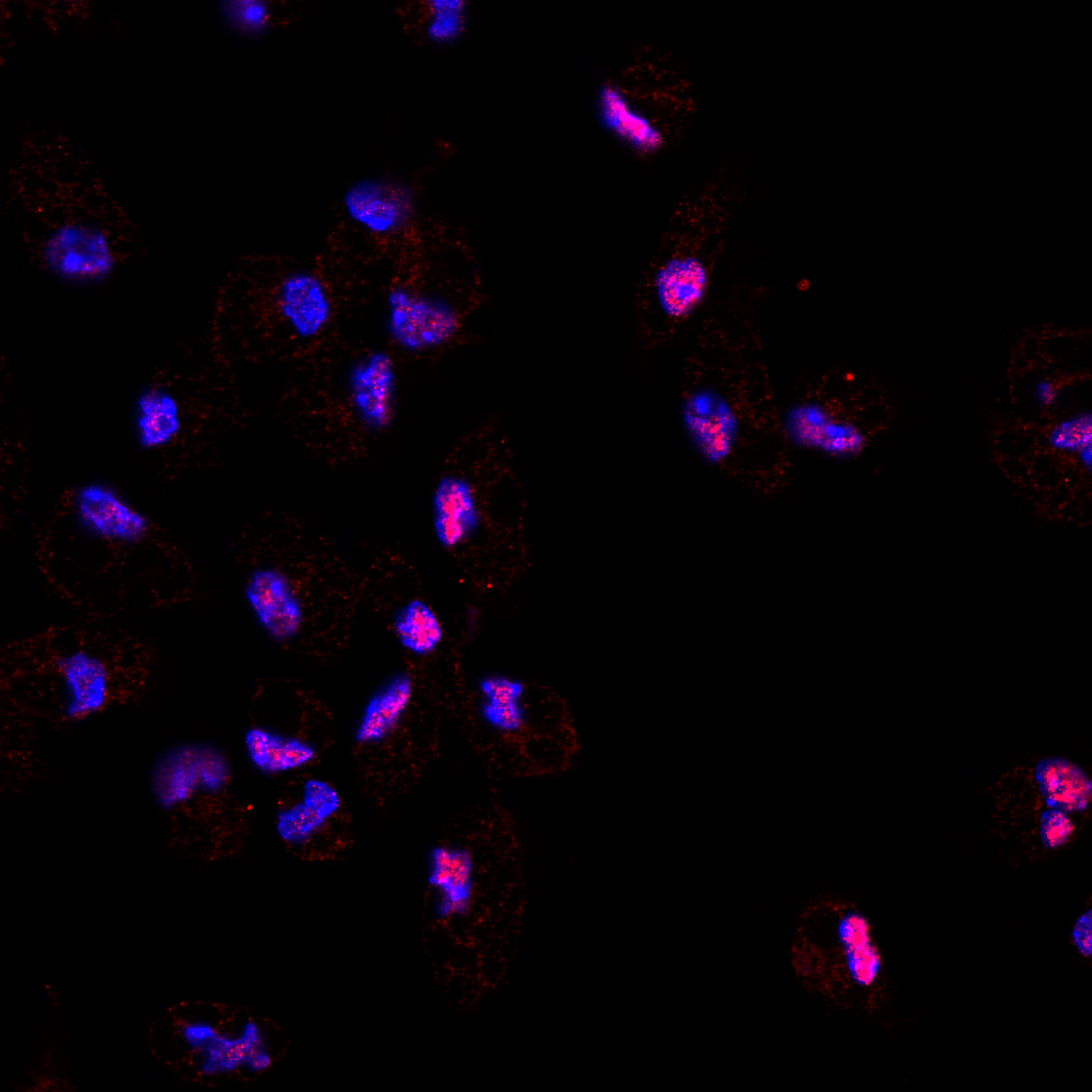

Supplement: Supplementary file 8 — Source Data for Figure 6 [file EMMM-15-e17198-s005.zip › Figure 6/6H-I/6H-p65 staining.pdf]

Figure 7G-LFD

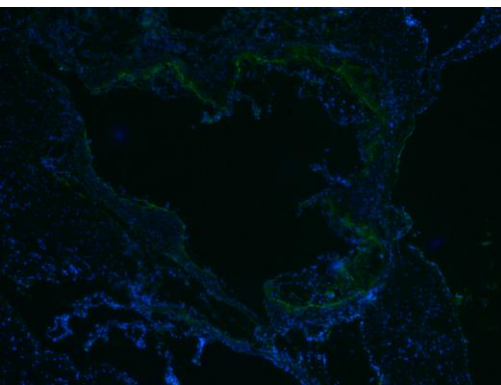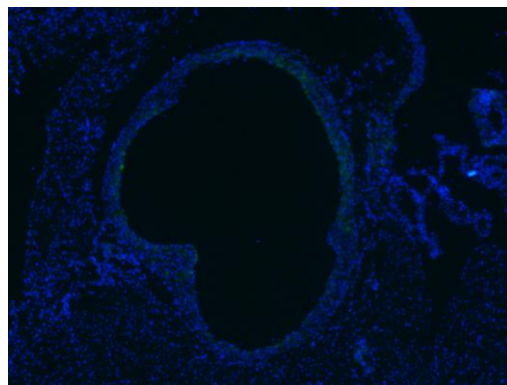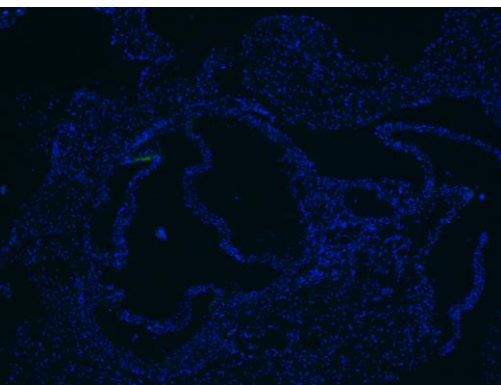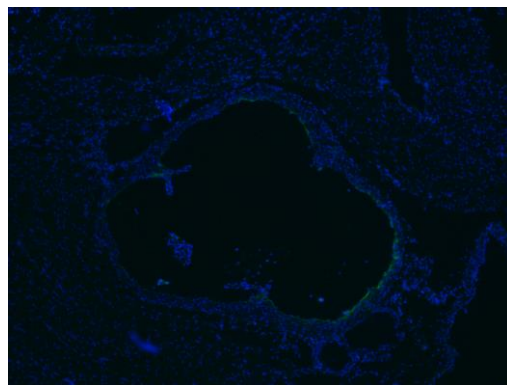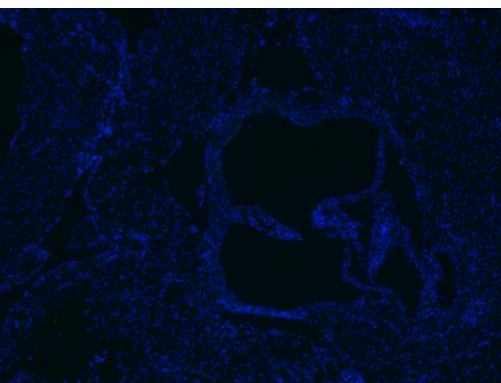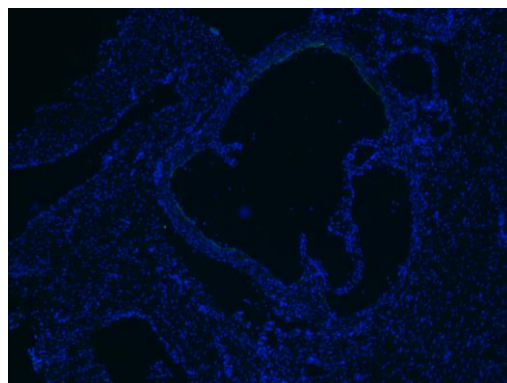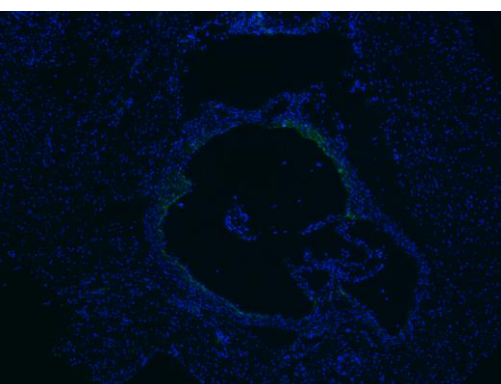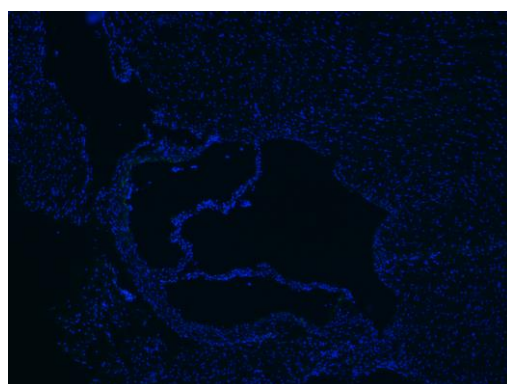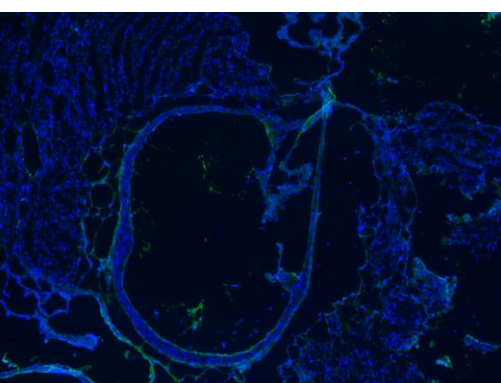

Figure 7G-HFD

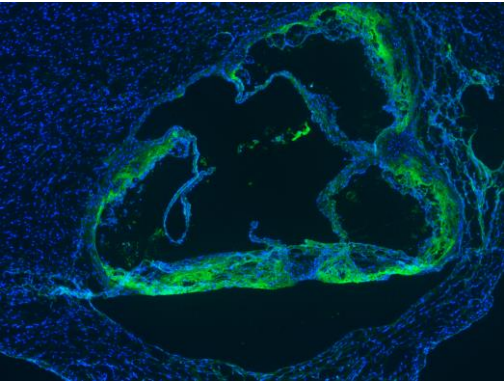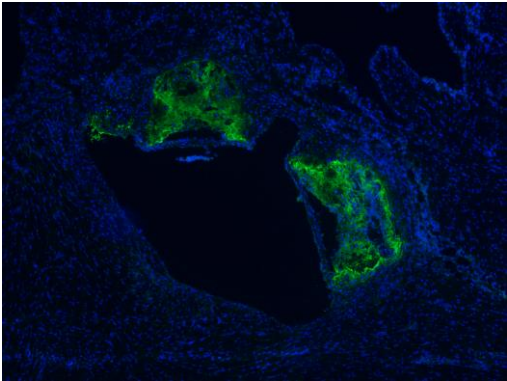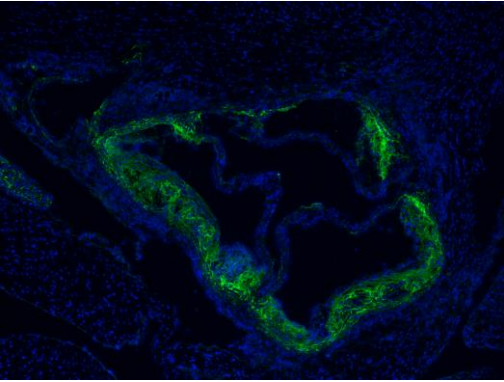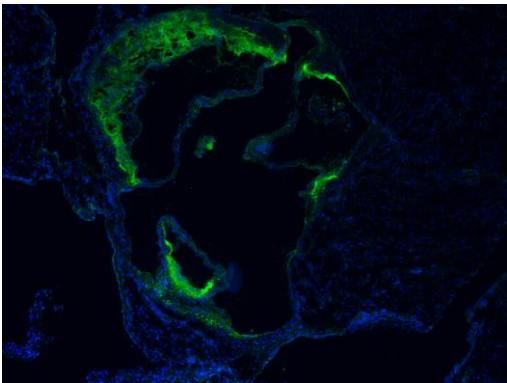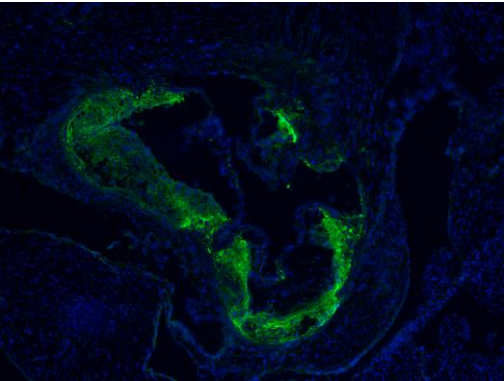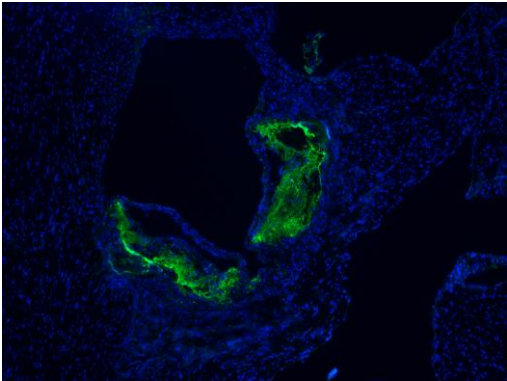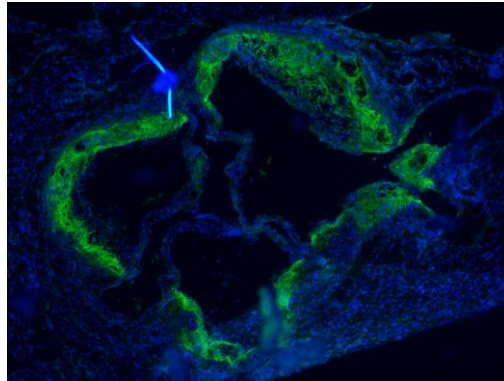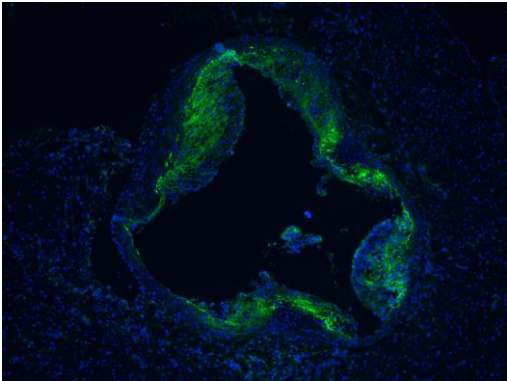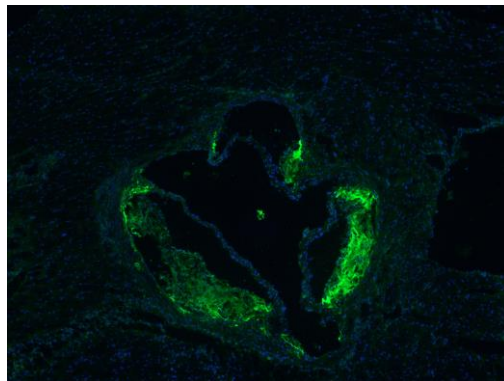

Figure 7G-HFD+DCLK1-IN-1

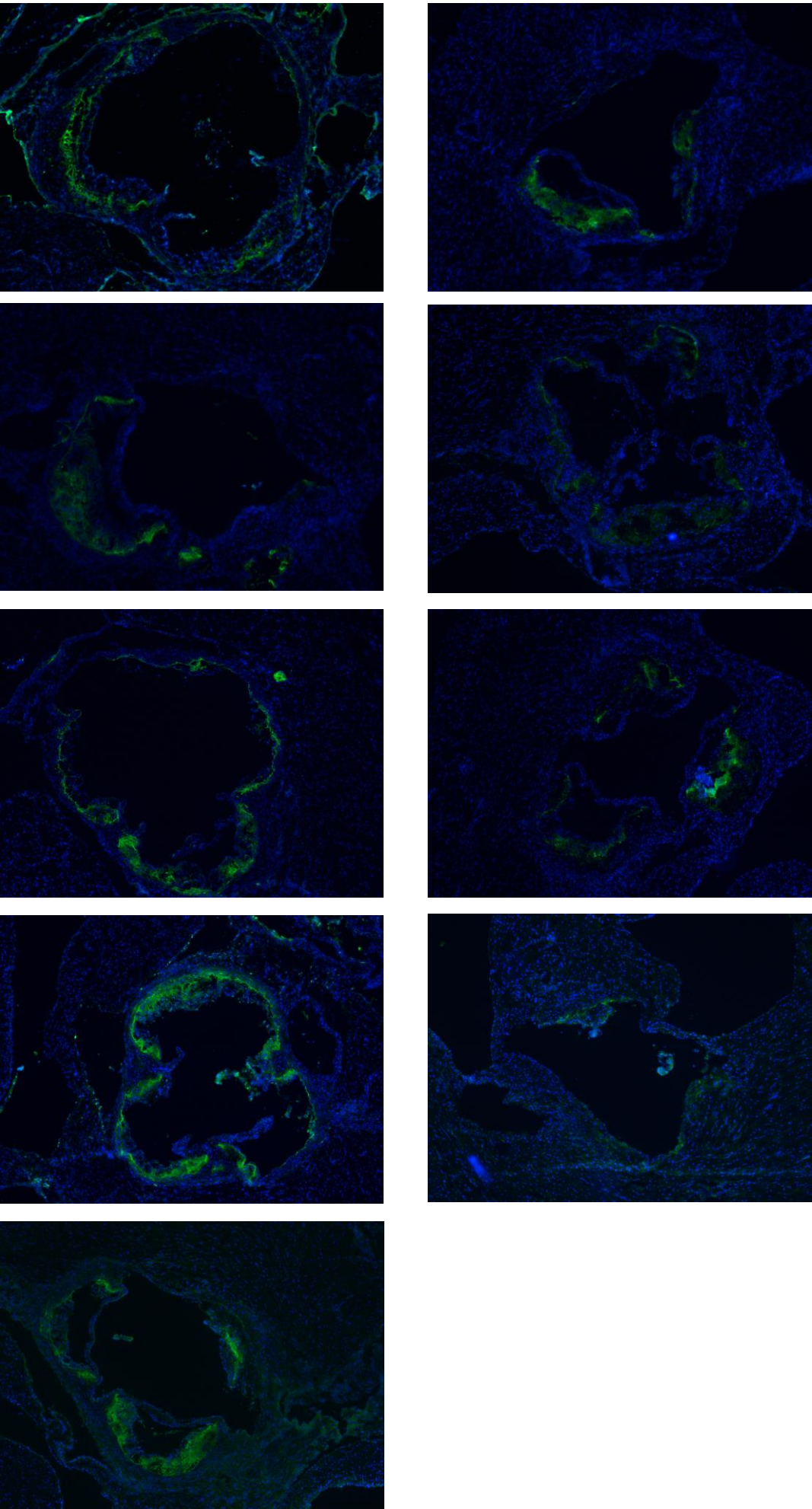

Supplement: Supplementary file 9 — Source Data for Figure 7 [file EMMM-15-e17198-s003.zip › EMM-2022-17198-V2-Figure_7_Source_Data-sd/7G-F480 staining.pdf]

Figure 7C-LFD

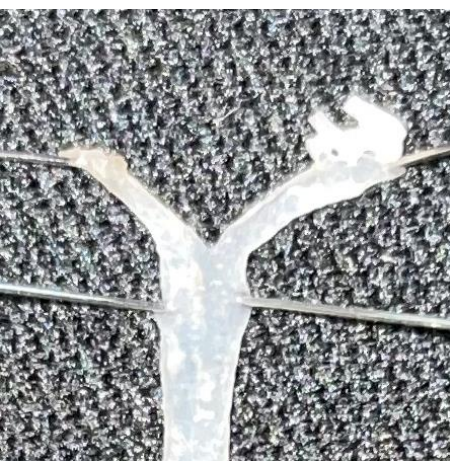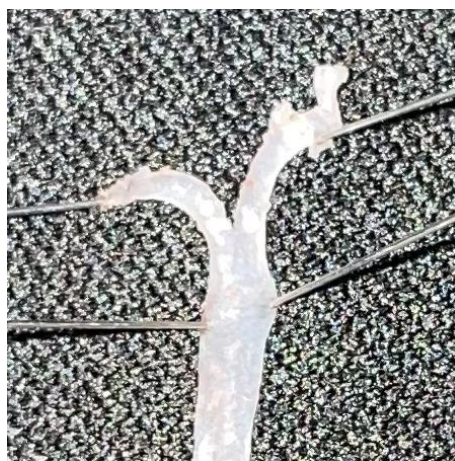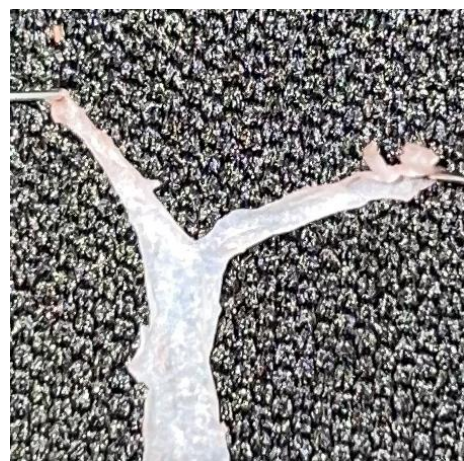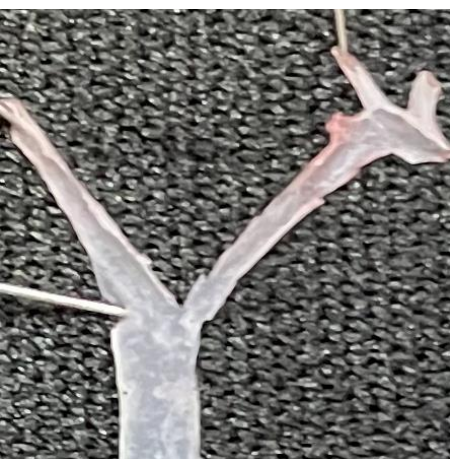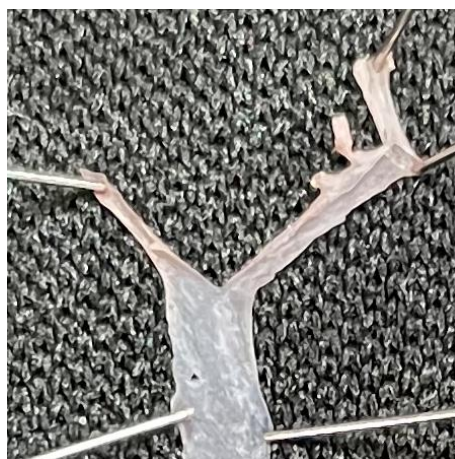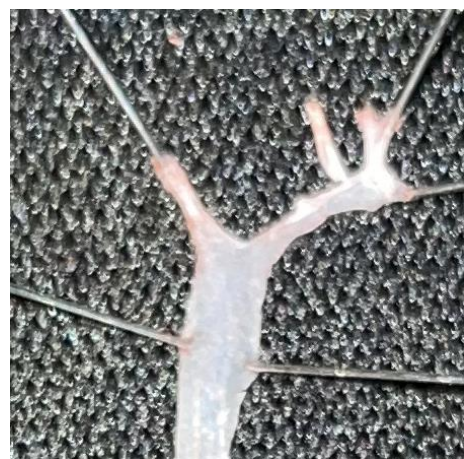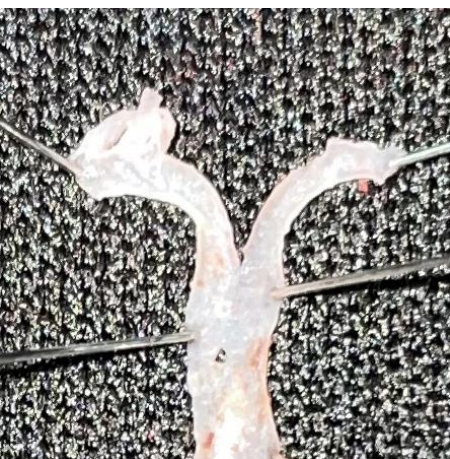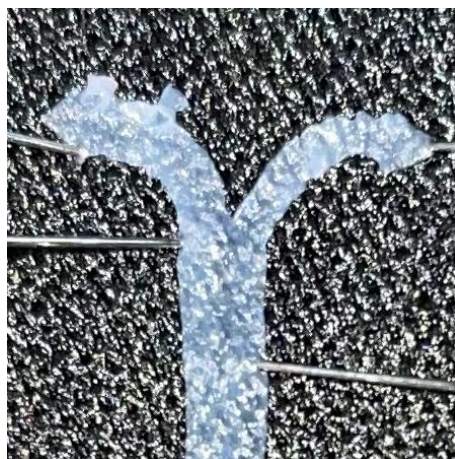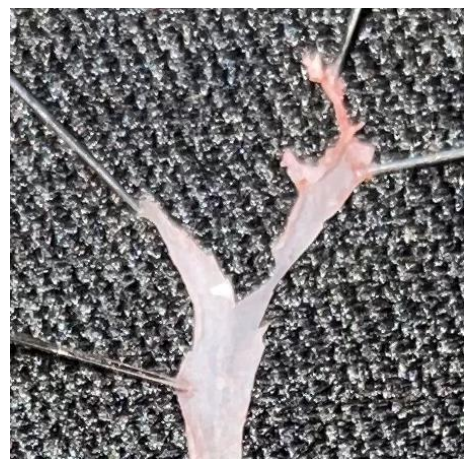

Figure 7C-HFD

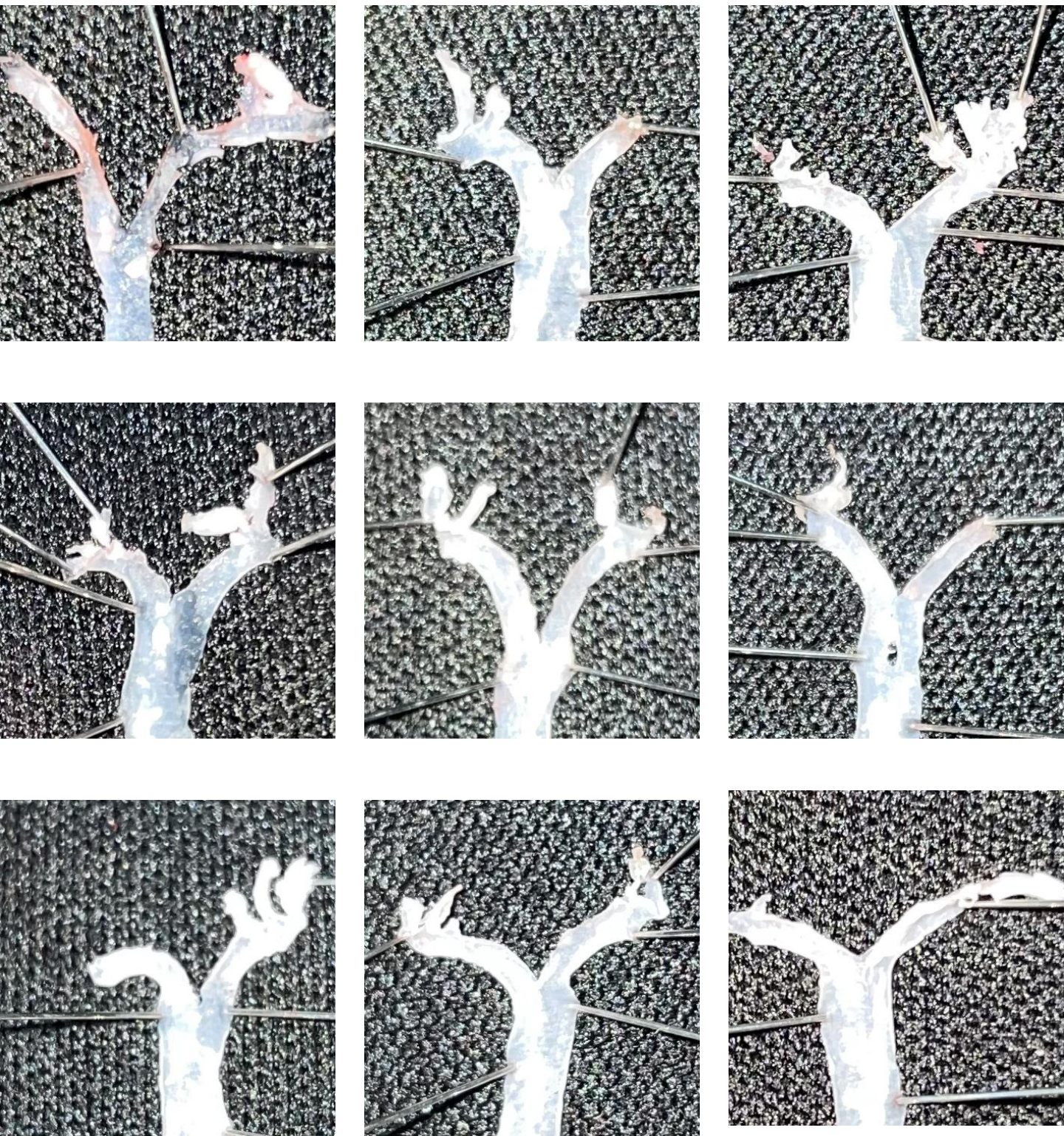

Figure 7C-HFD+DCLK1-IN-1

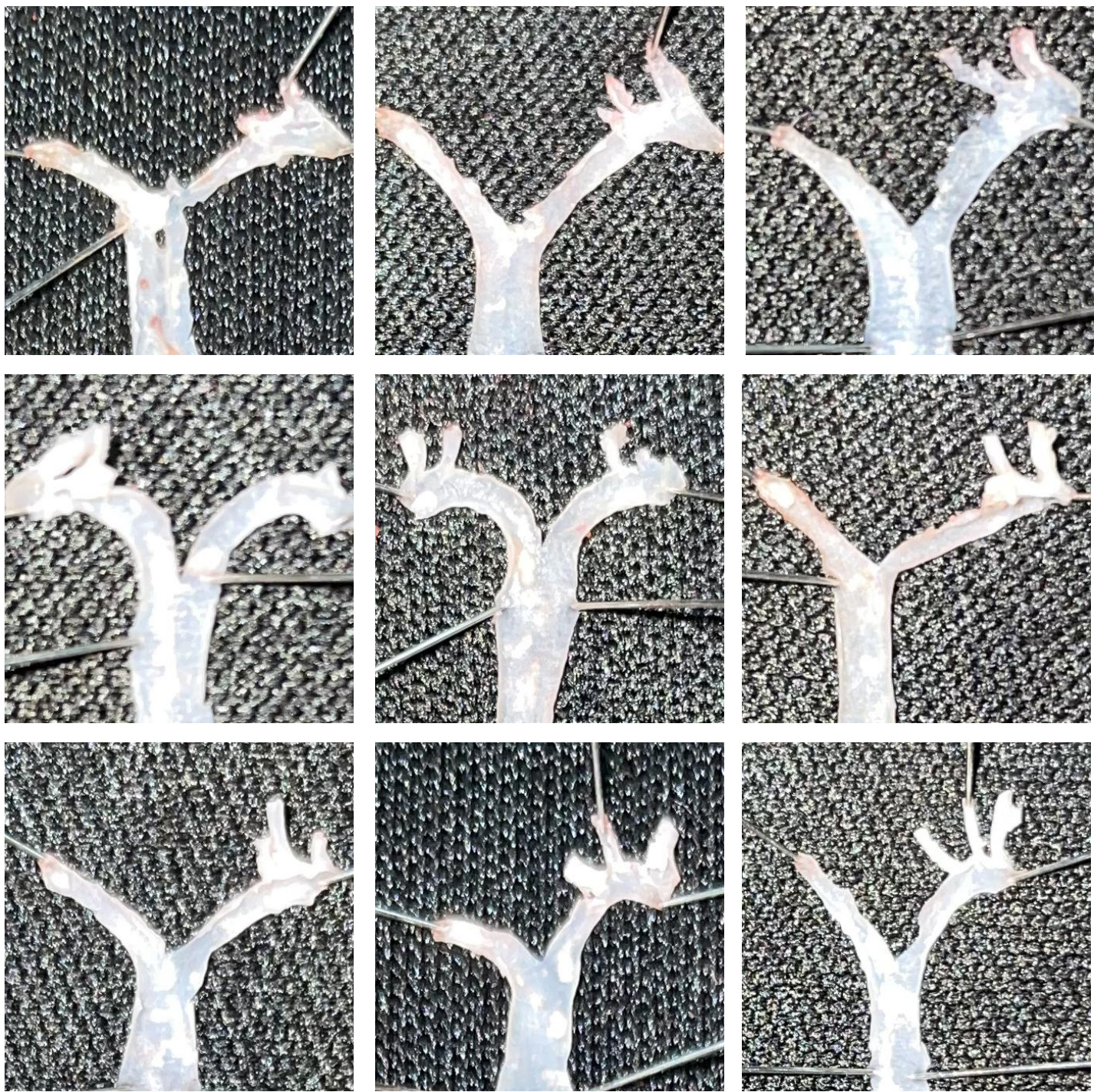

Supplement: Supplementary file 9 — Source Data for Figure 7 [file EMMM-15-e17198-s003.zip › EMM-2022-17198-V2-Figure_7_Source_Data-sd/7C-D/7C-aotic arches.pdf]

Figure 7J-LFD

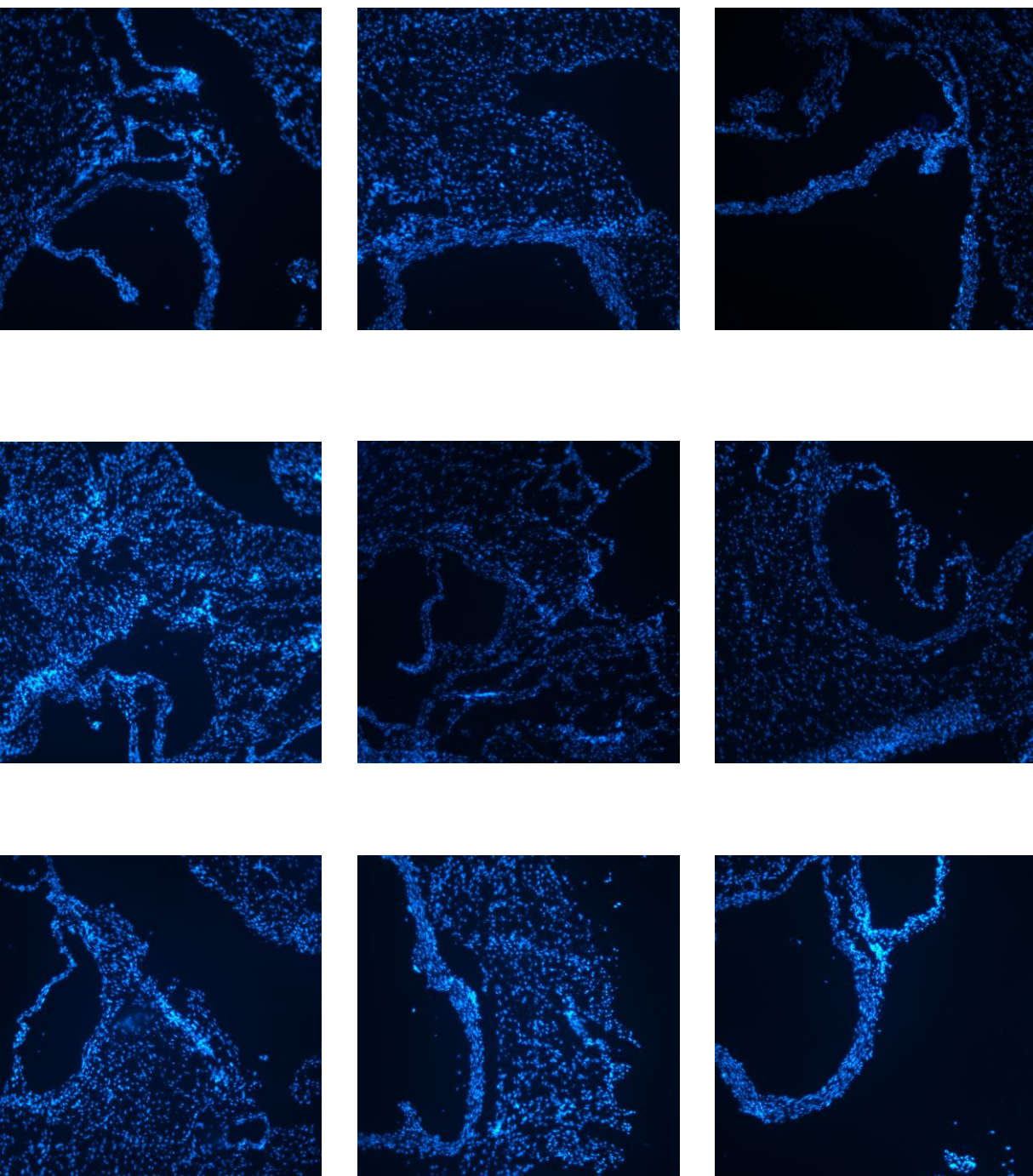

Figure 7J-HFD

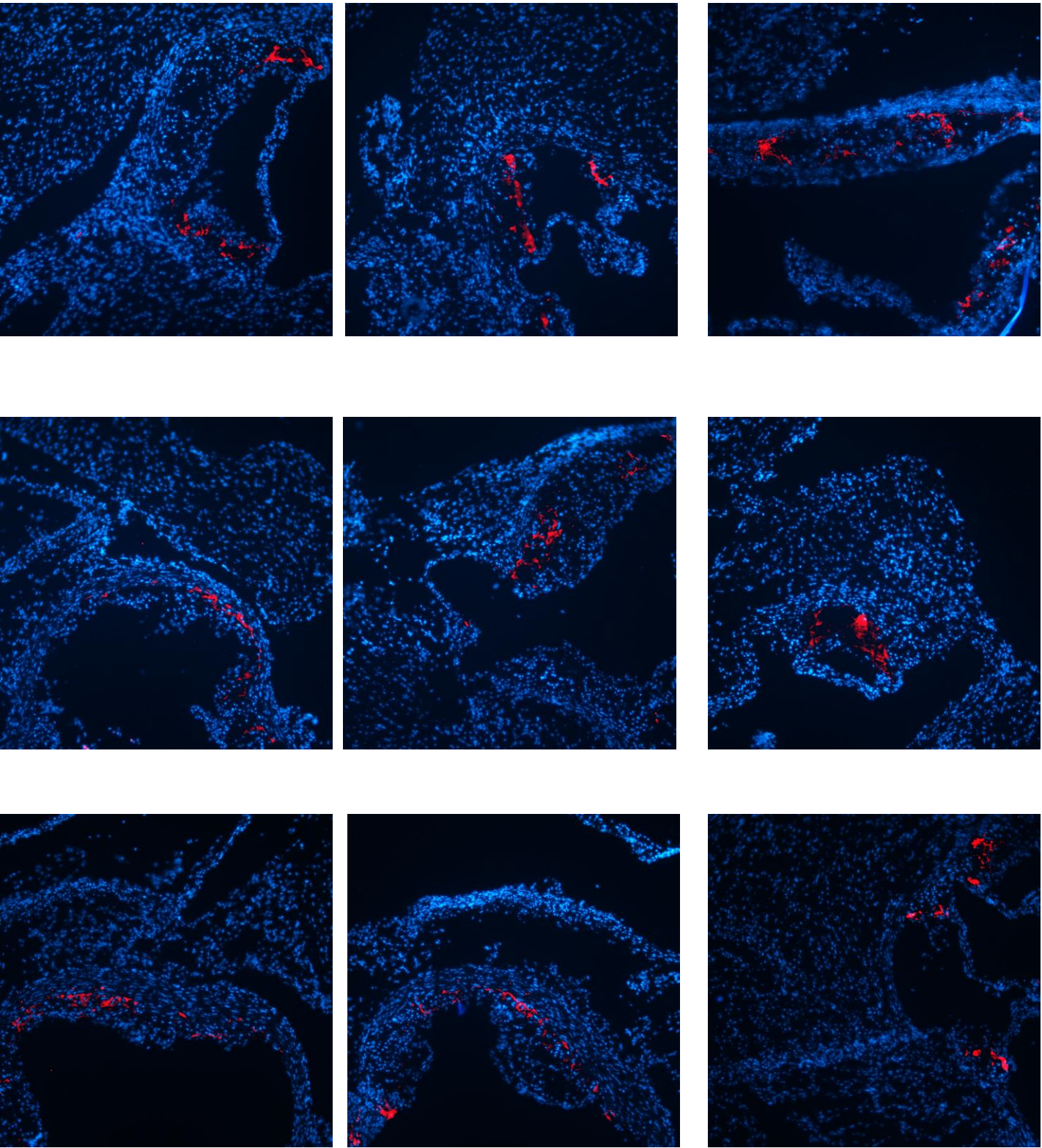

Figure 7J-HFD+DCLK1-IN-1

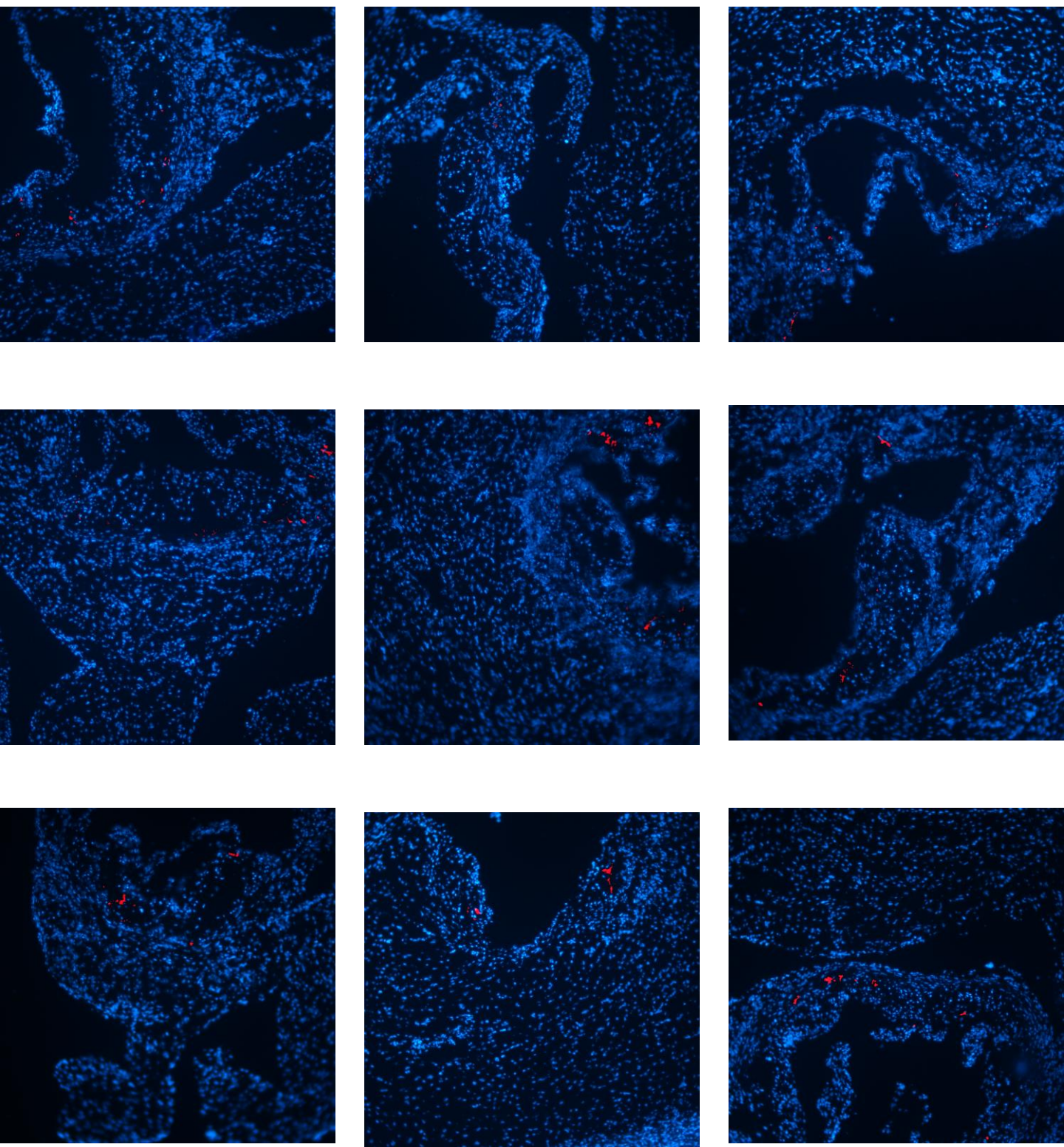

Supplement: Supplementary file 9 — Source Data for Figure 7 [file EMMM-15-e17198-s003.zip › EMM-2022-17198-V2-Figure_7_Source_Data-sd/7J/7J-p65 staining.pdf]

Figure 7E-LFD

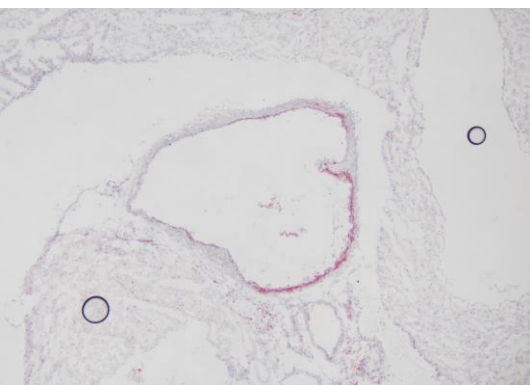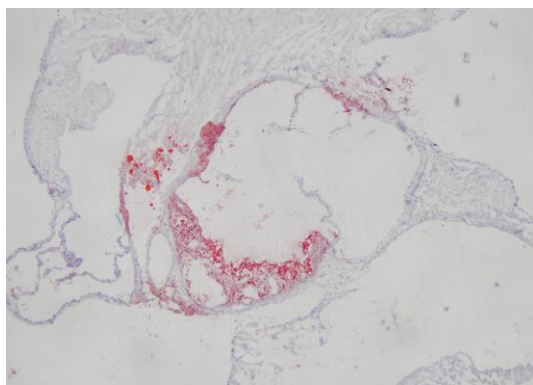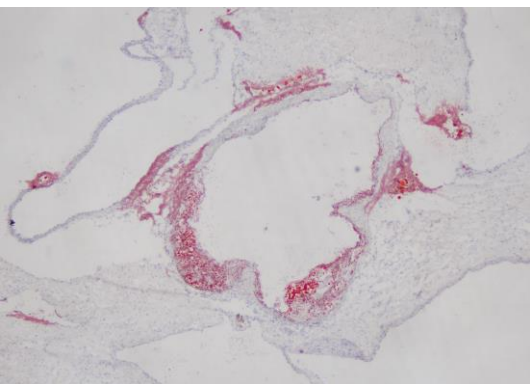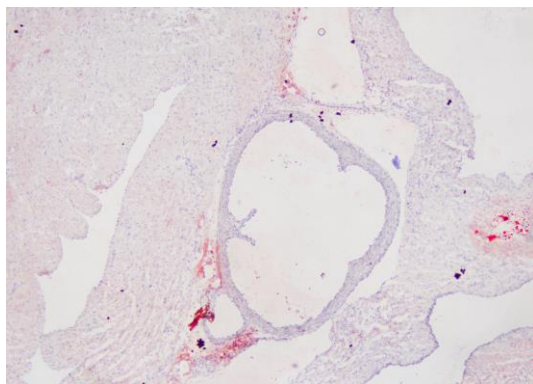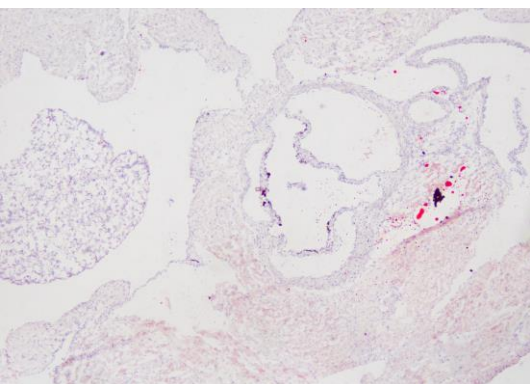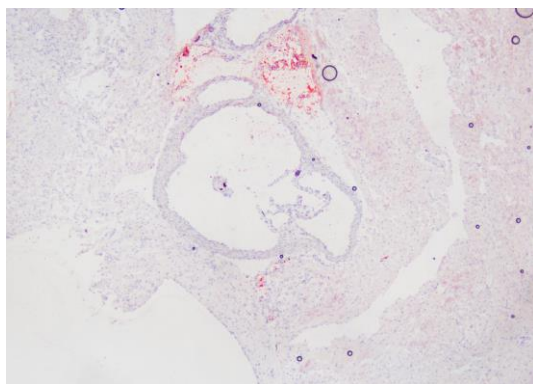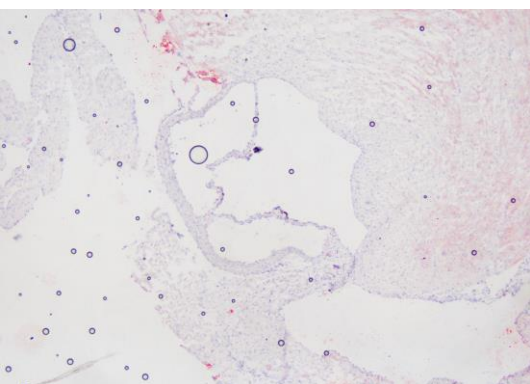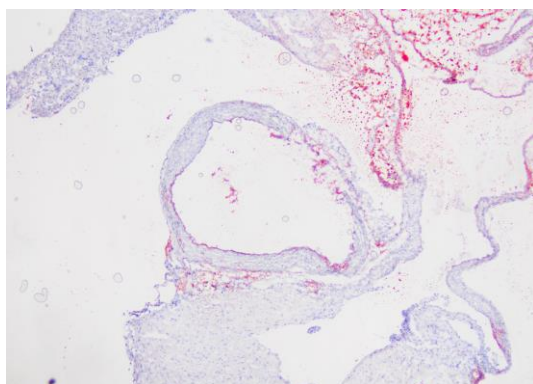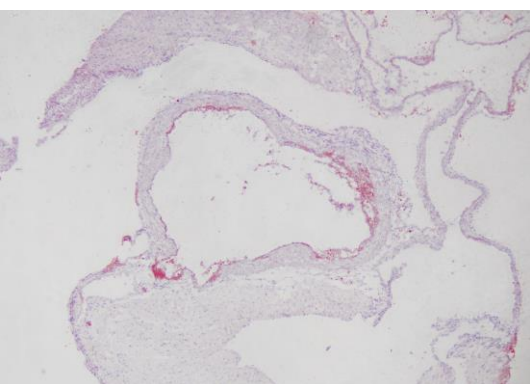

Figure 7E-HFD

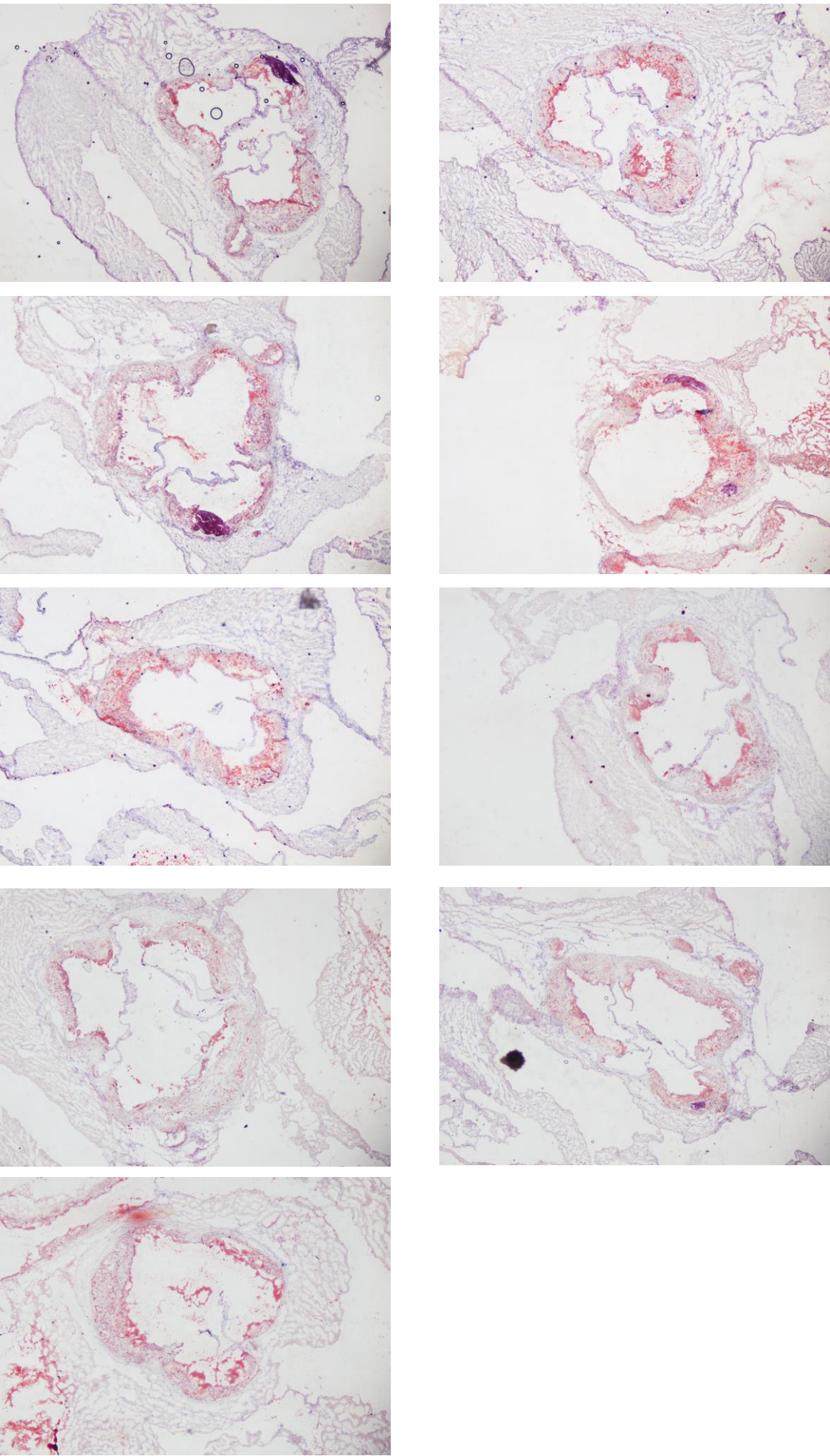

Figure 7E-HFD+DCLK1-IN-1

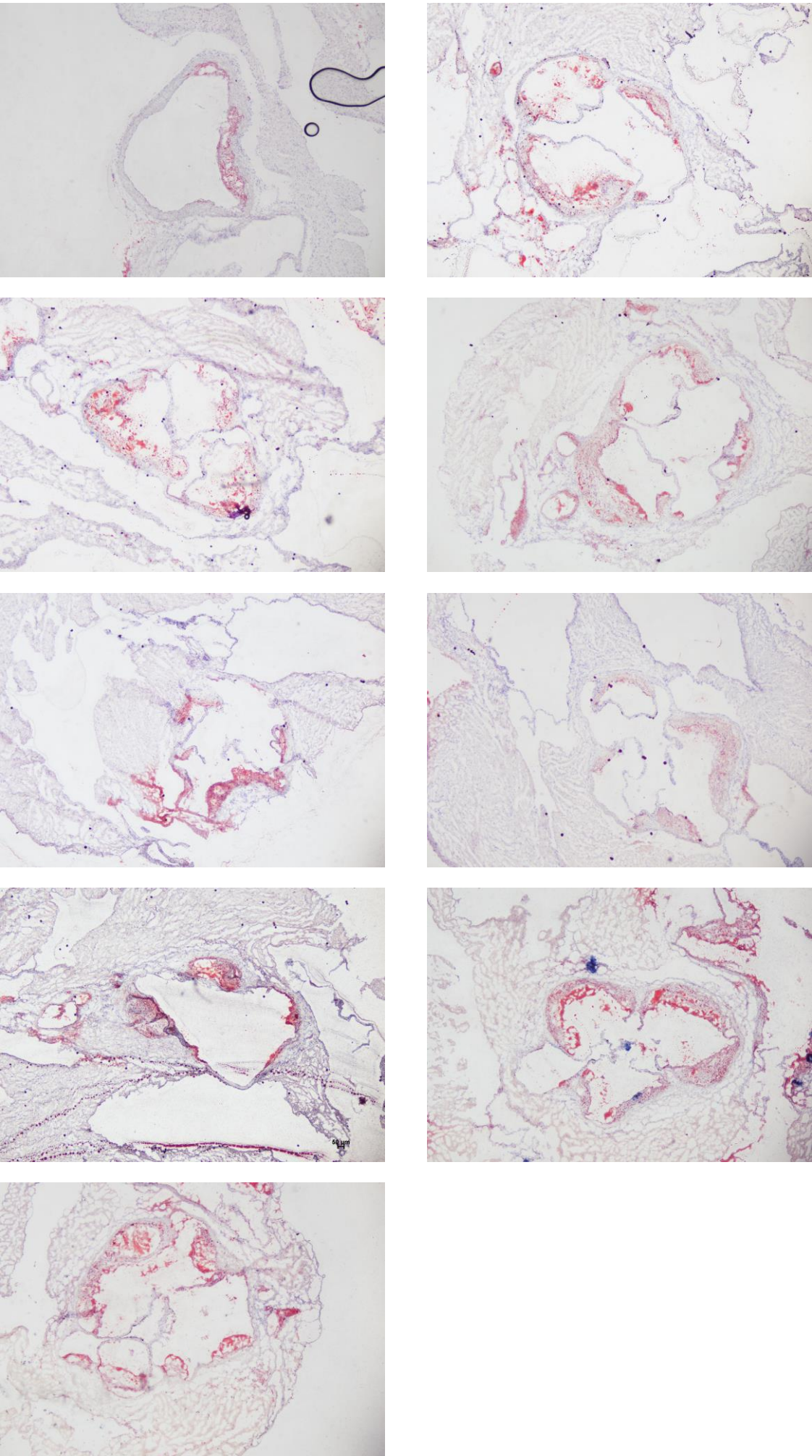

Supplement: Supplementary file 9 — Source Data for Figure 7 [file EMMM-15-e17198-s003.zip › EMM-2022-17198-V2-Figure_7_Source_Data-sd/7E/7E-Oil Red O.pdf]

Figure 7A-LFD

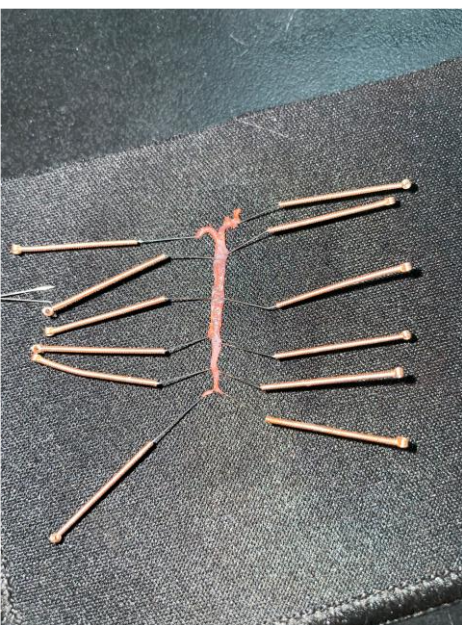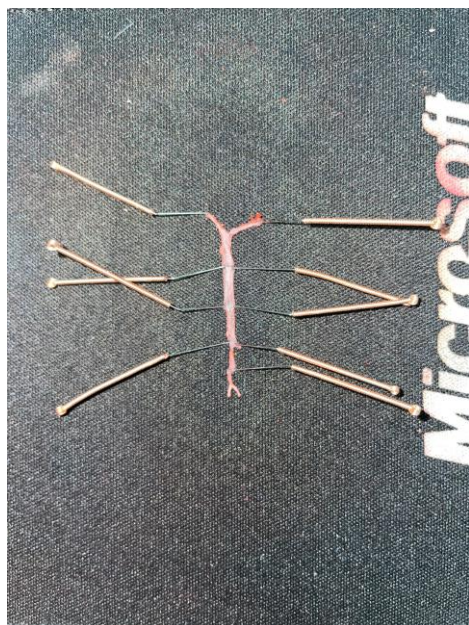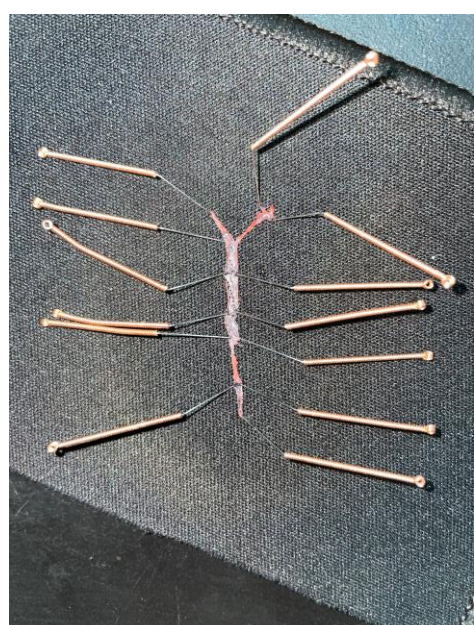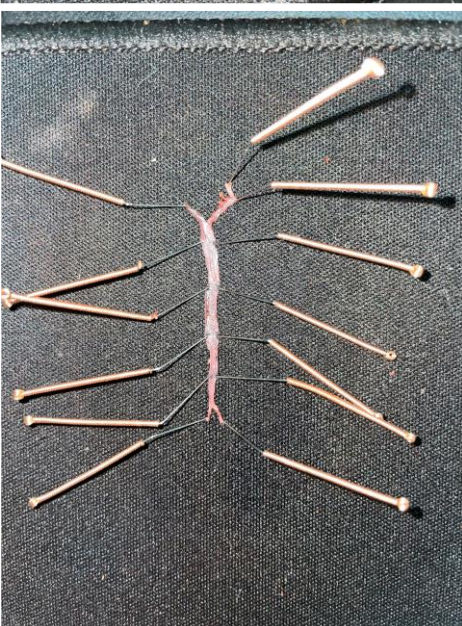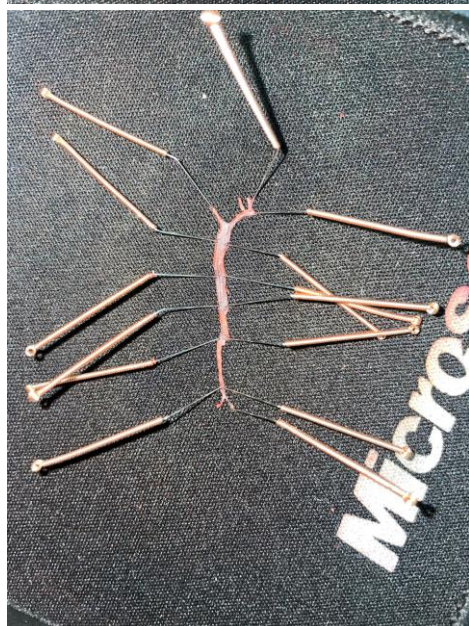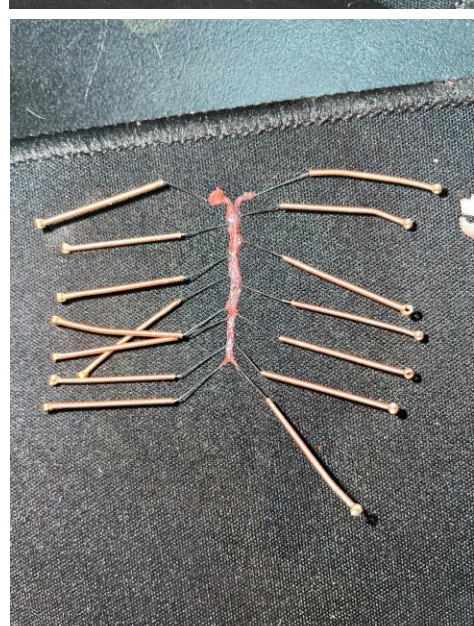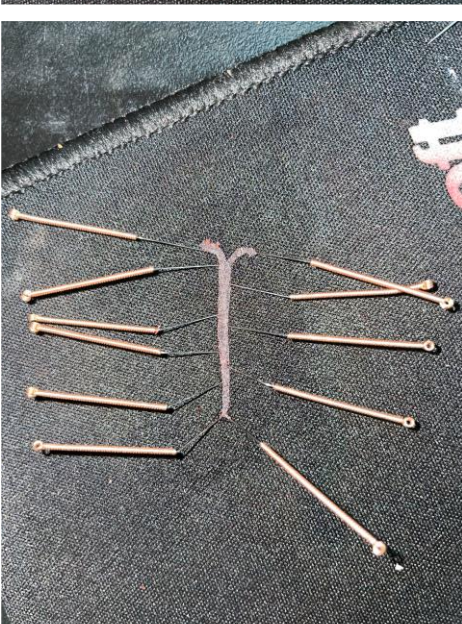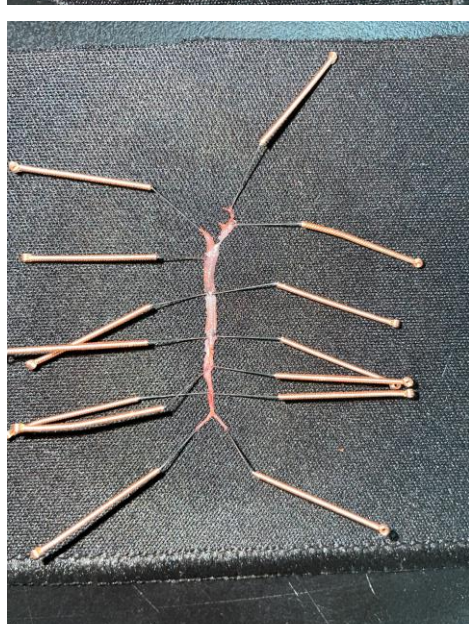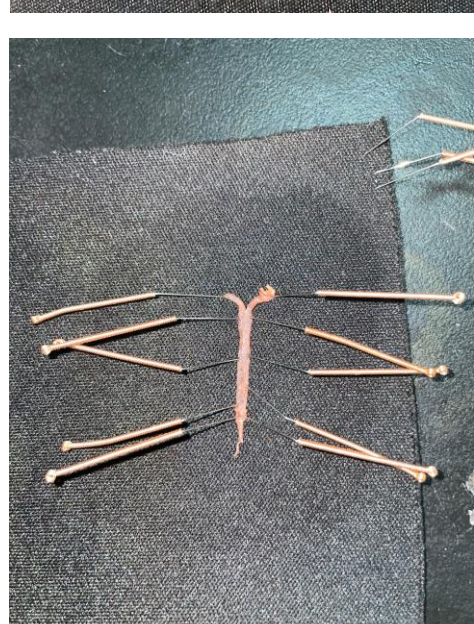

Figure 7A-HFD

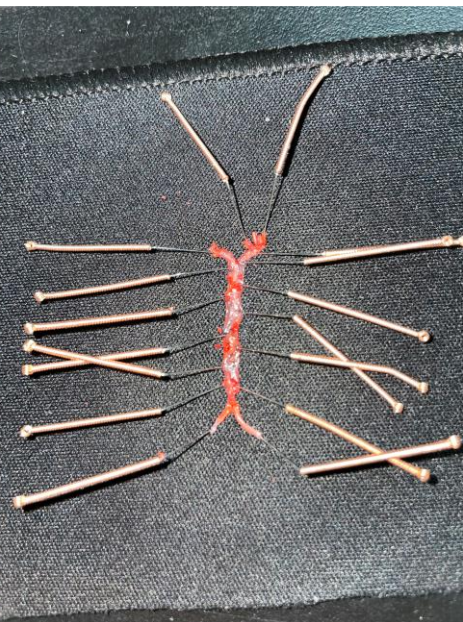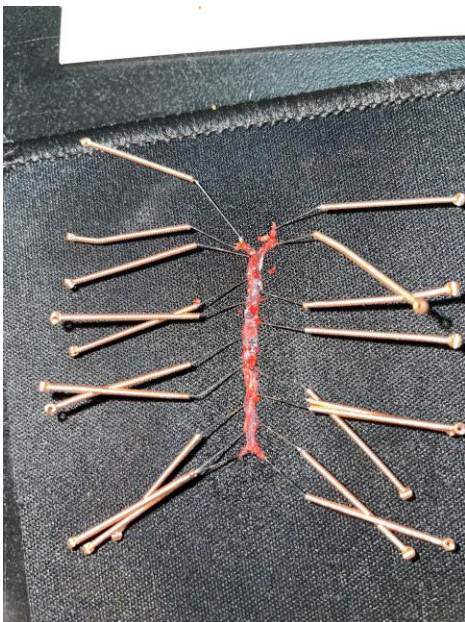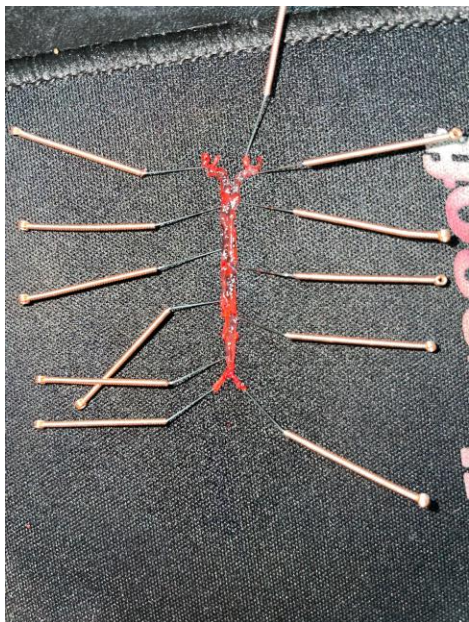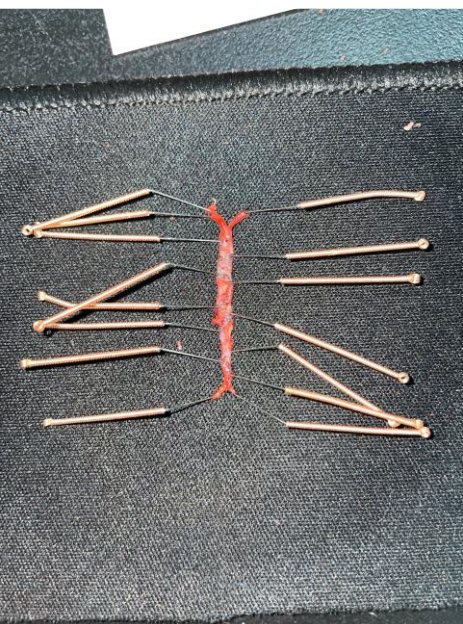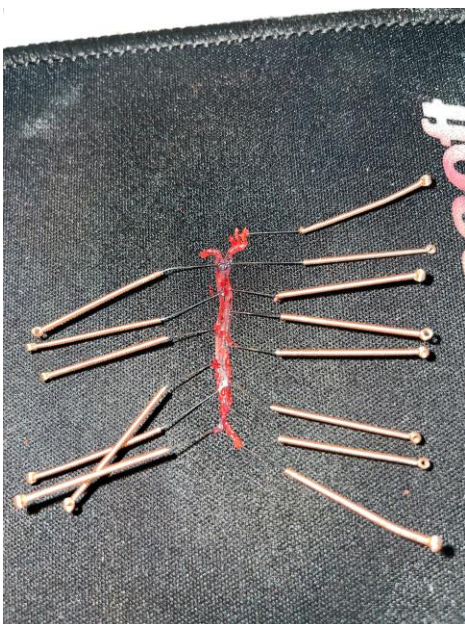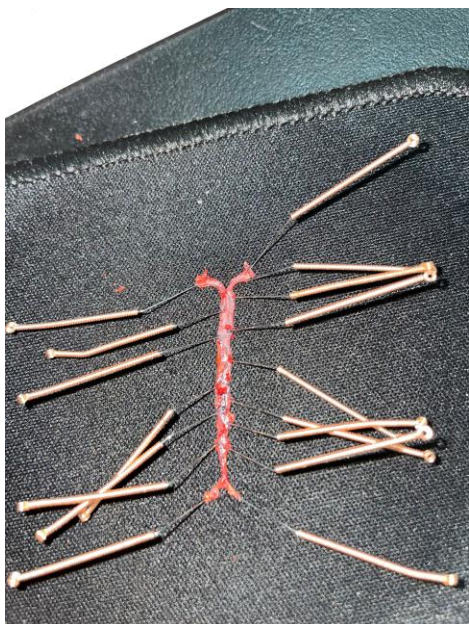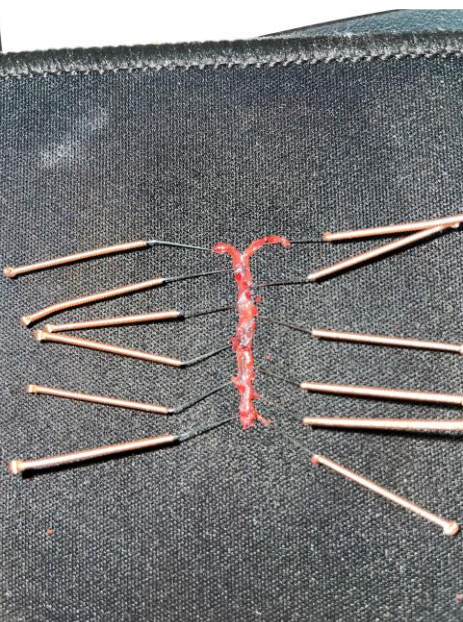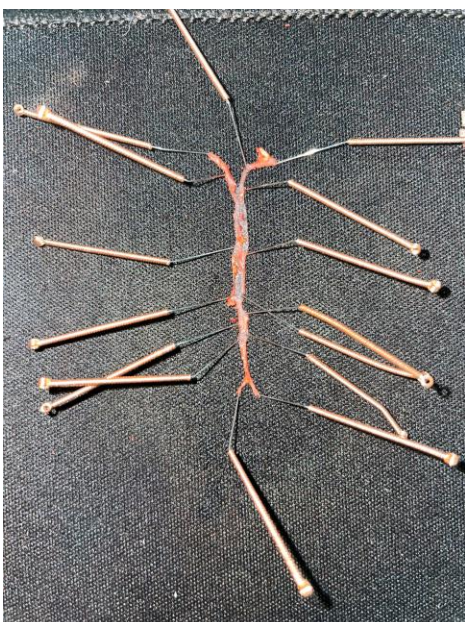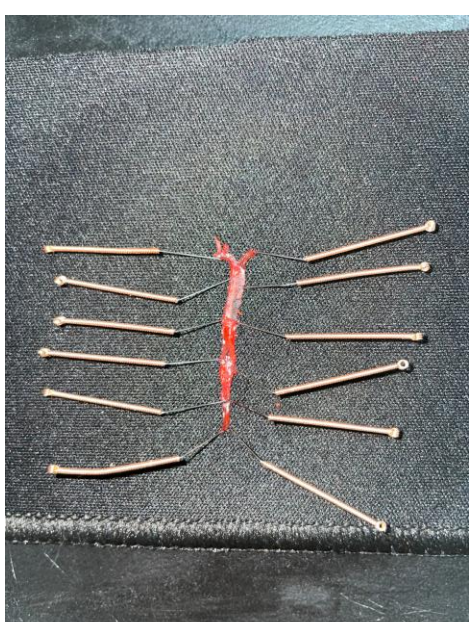

Figure 7A-HFD+DCLK1-IN-1

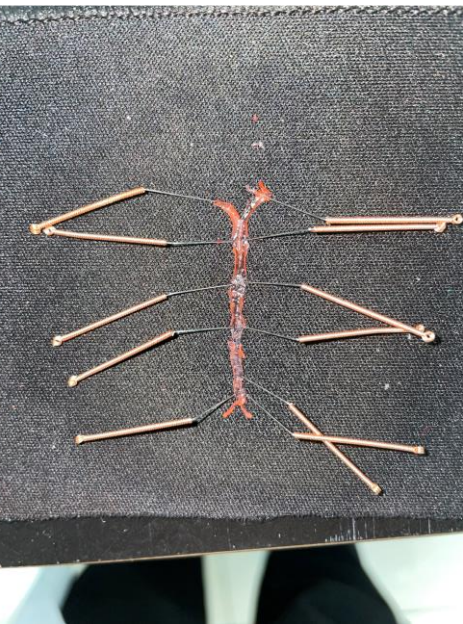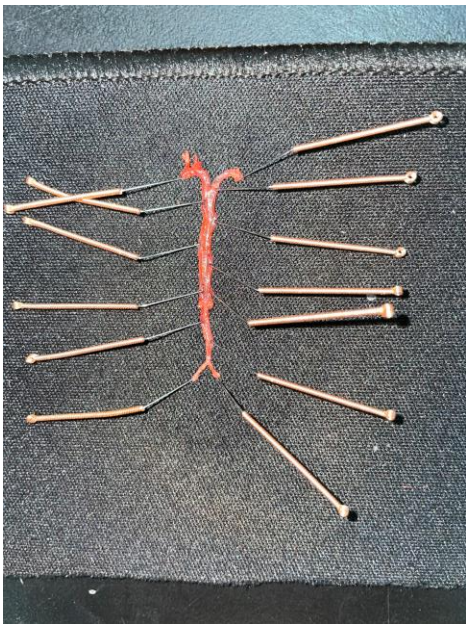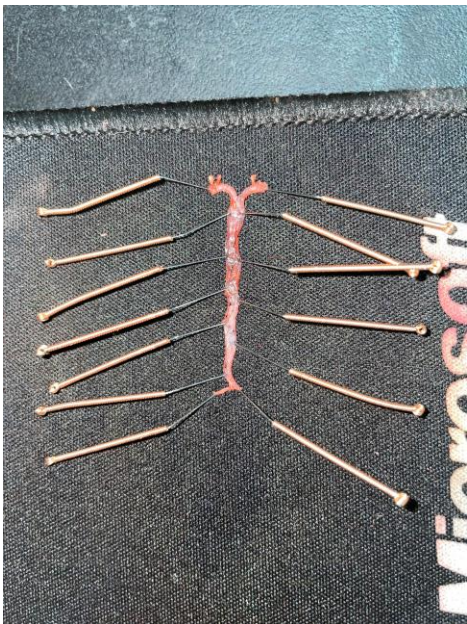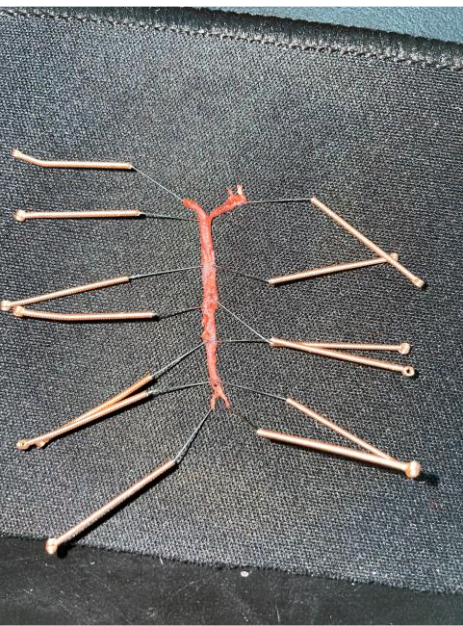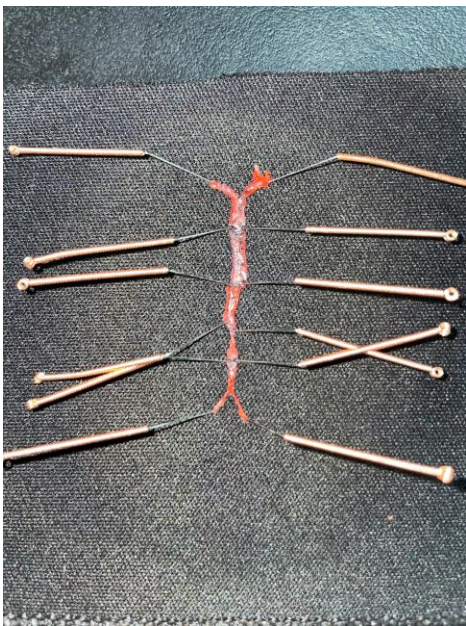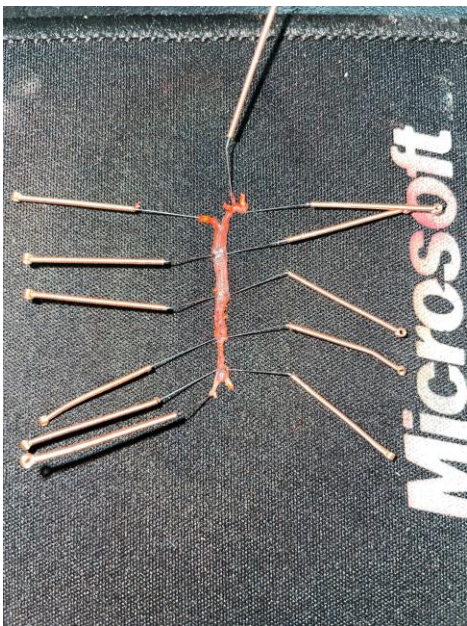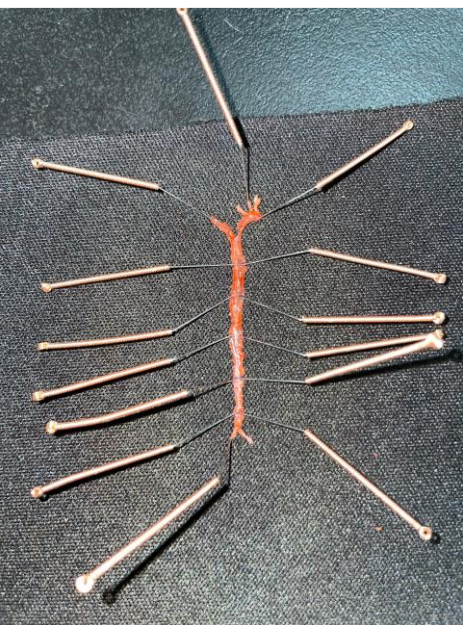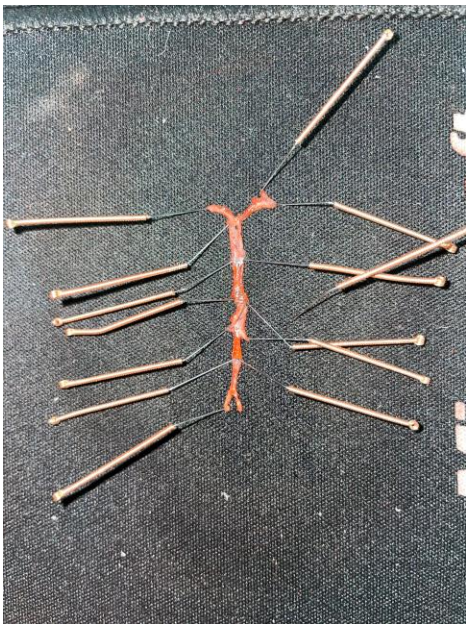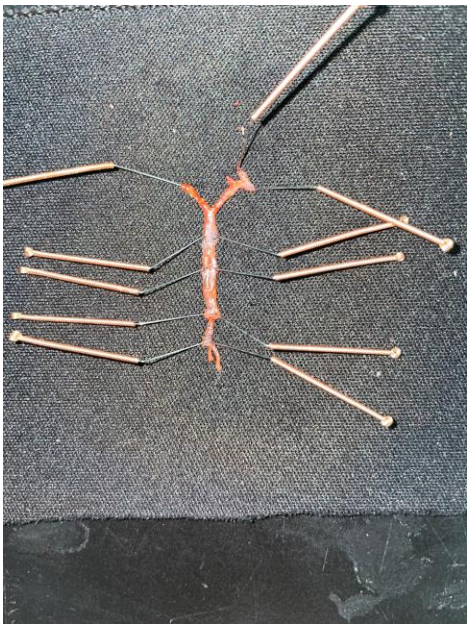

Supplement: Supplementary file 9 — Source Data for Figure 7 [file EMMM-15-e17198-s003.zip › EMM-2022-17198-V2-Figure_7_Source_Data-sd/7A-B/7A-Oil Red O.pdf]

Figure 7I-LFD

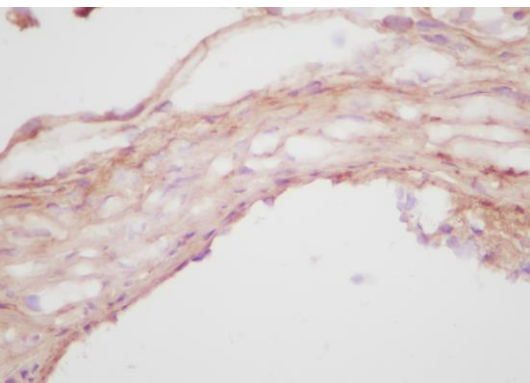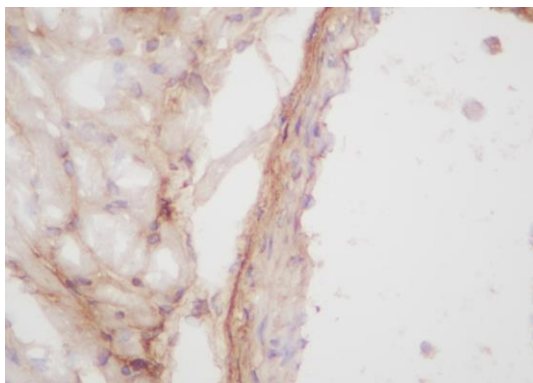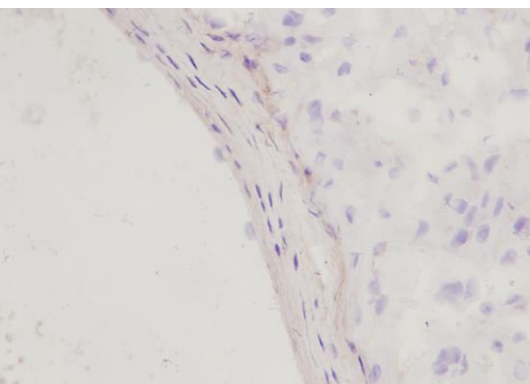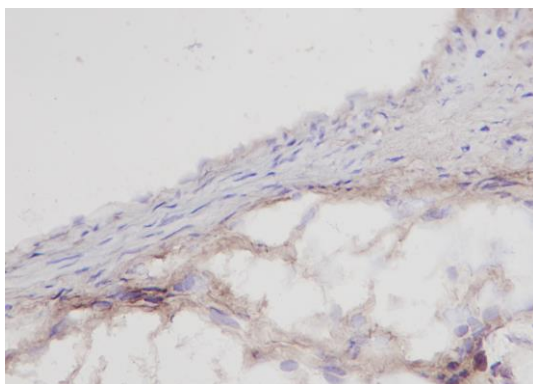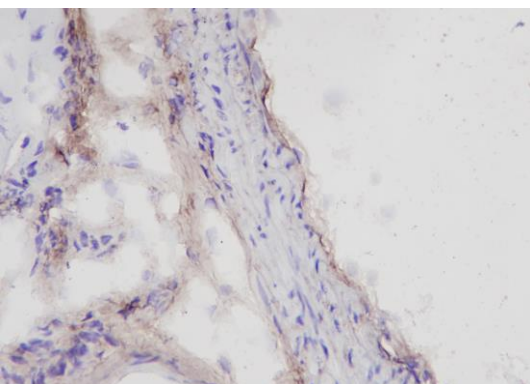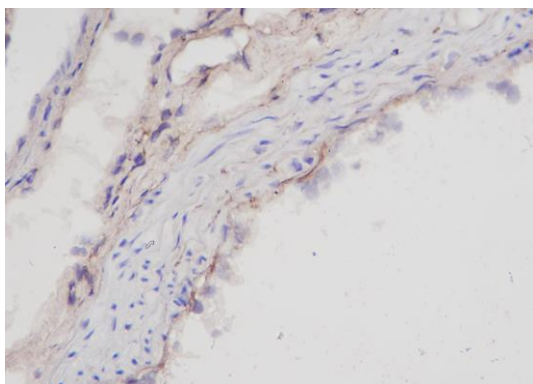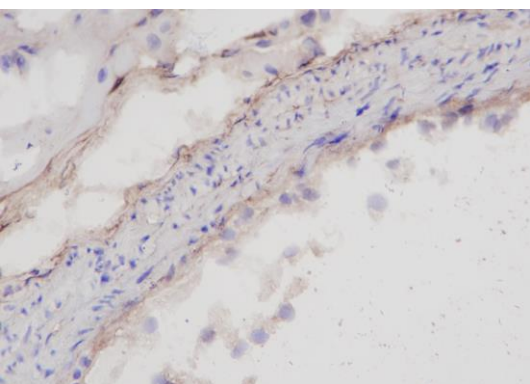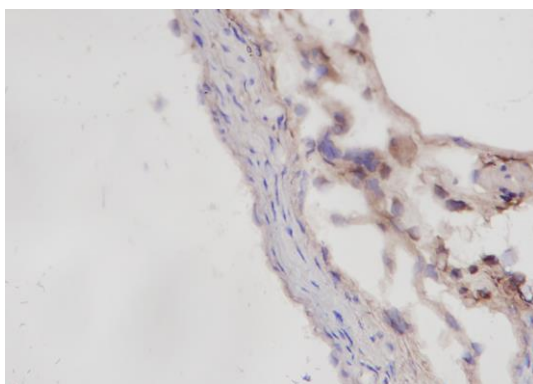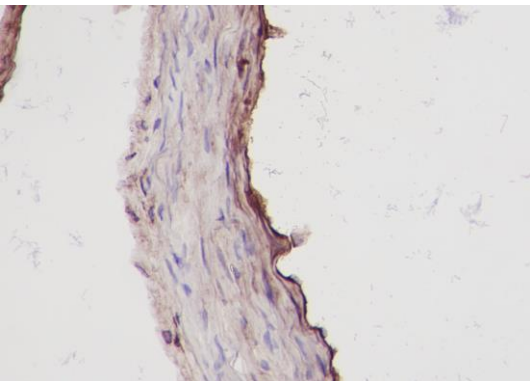

Figure 7I-HFD

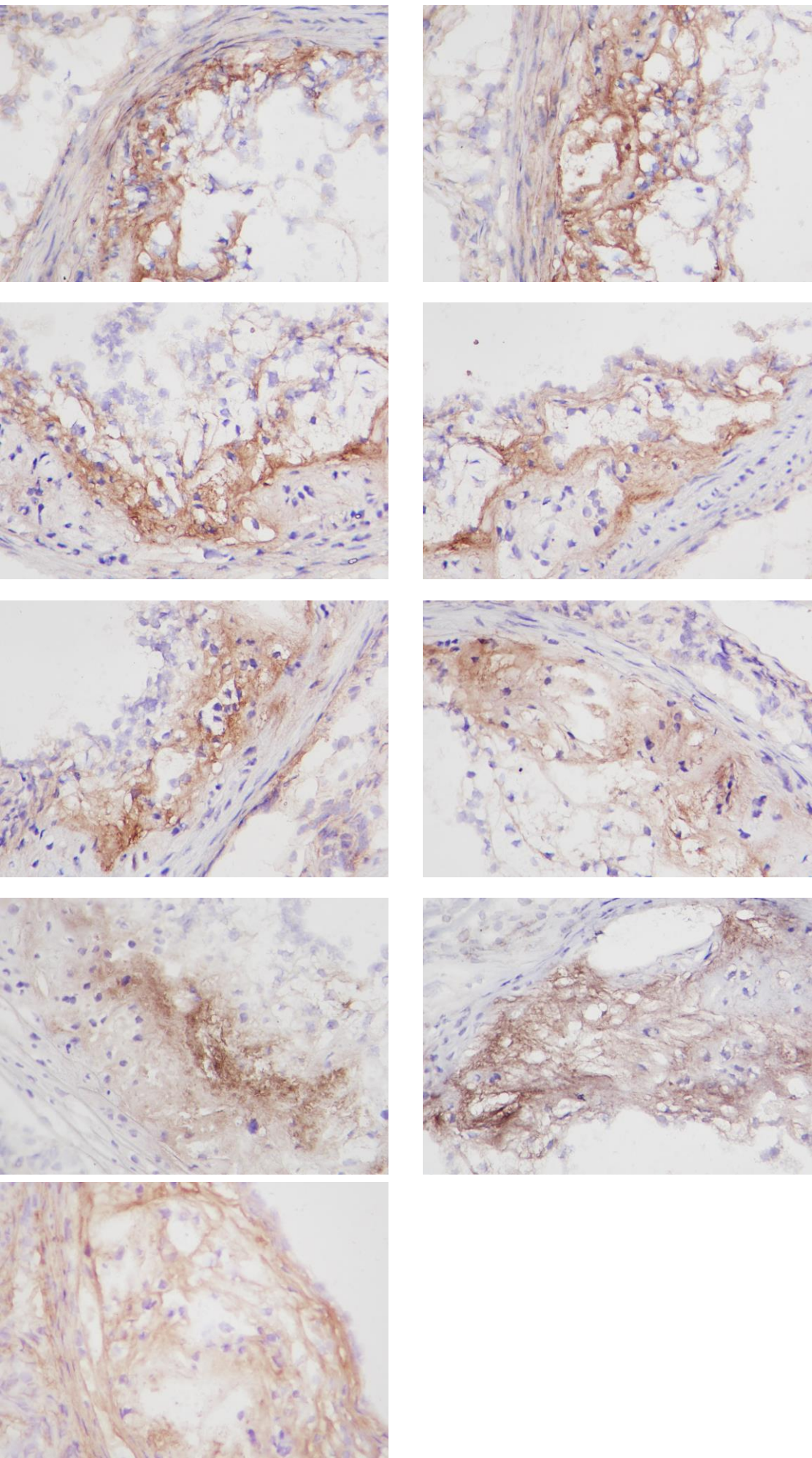

Figure 7I-HFD+DCLK1-IN-1

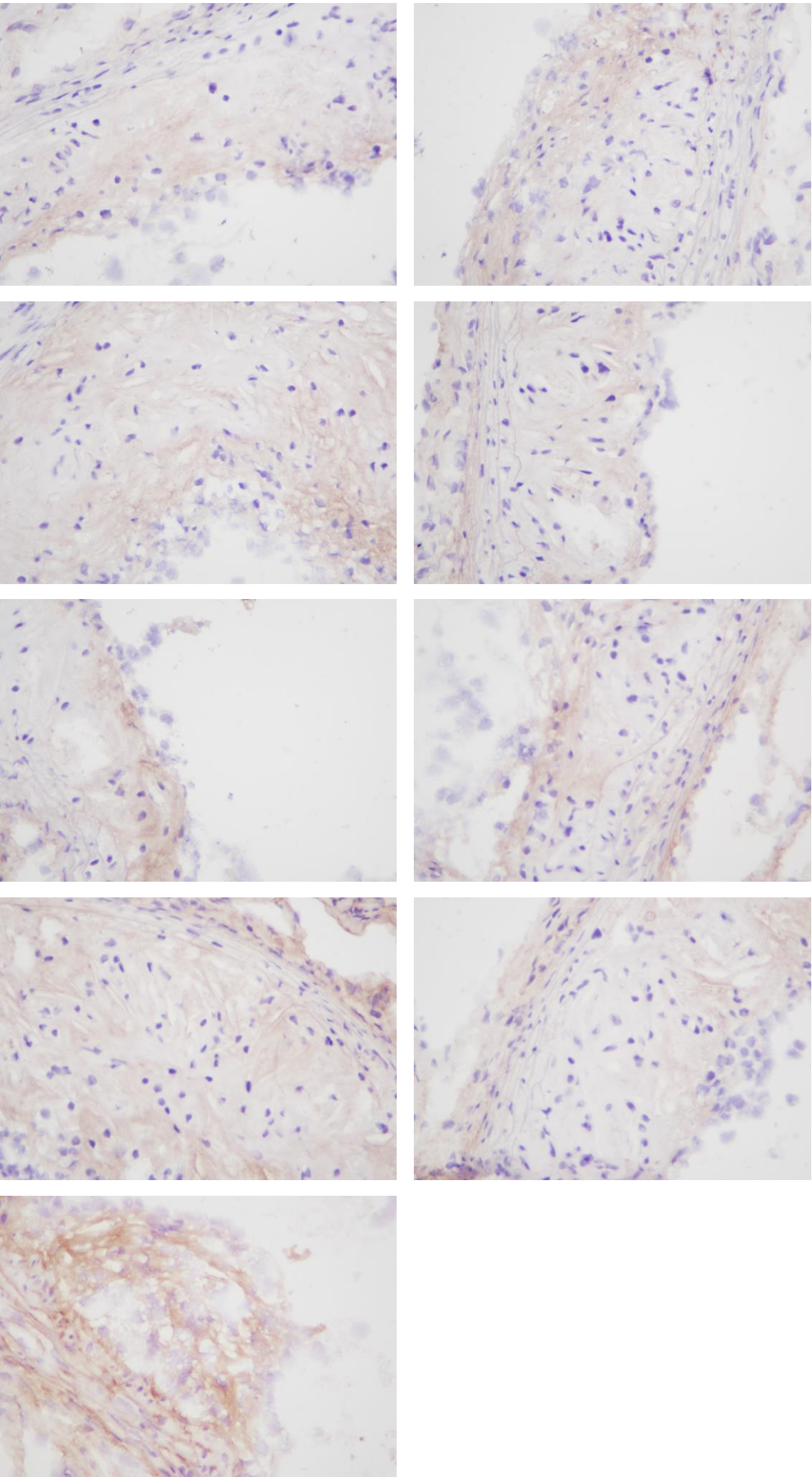

Supplement: Supplementary file 9 — Source Data for Figure 7 [file EMMM-15-e17198-s003.zip › EMM-2022-17198-V2-Figure_7_Source_Data-sd/7I/7I-Ly6C.pdf]

Figure 7F-LFD

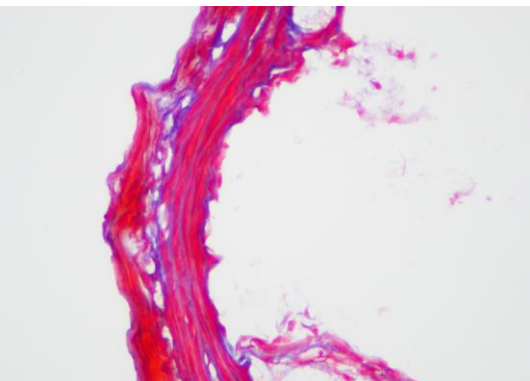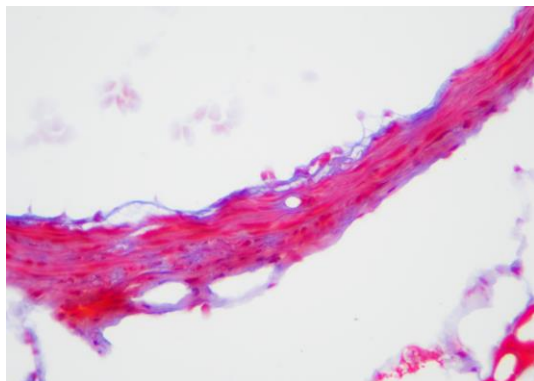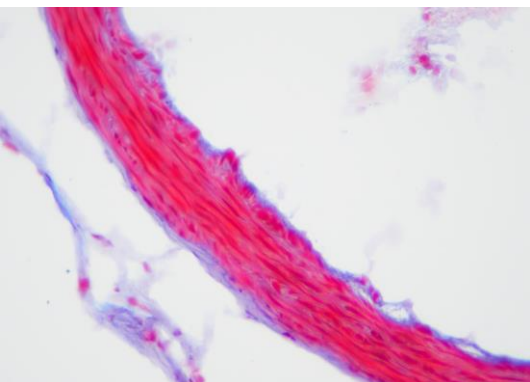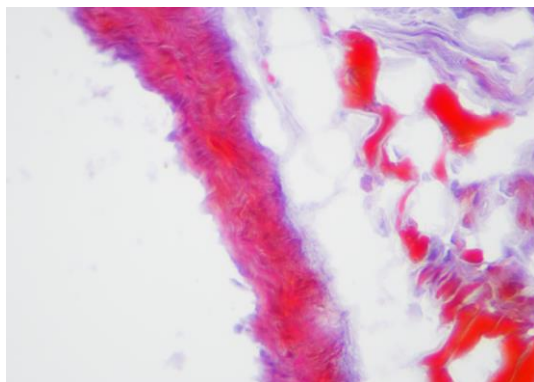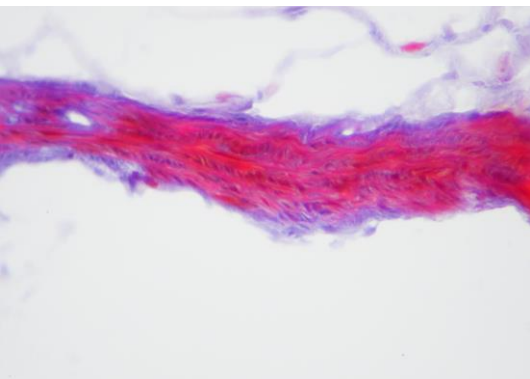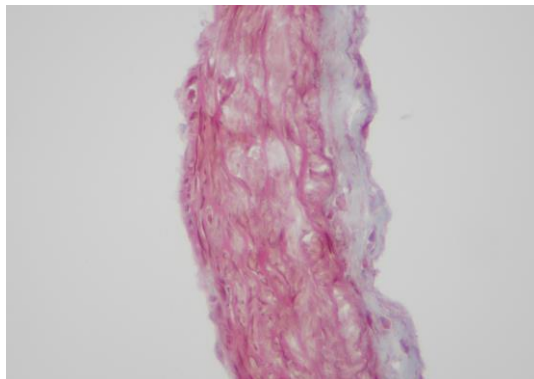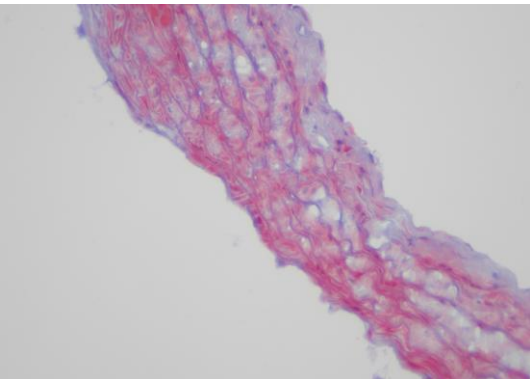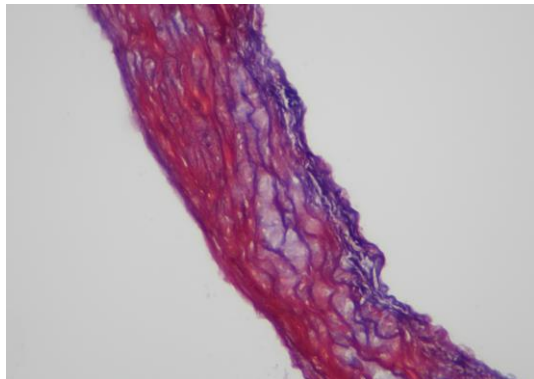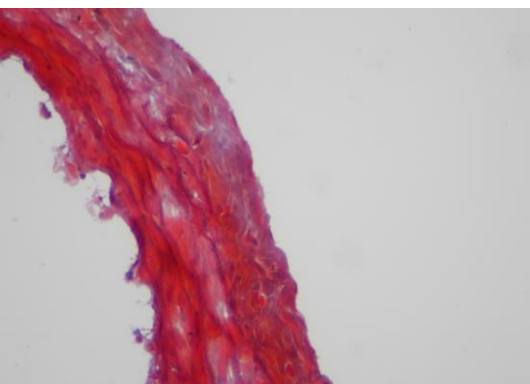

Figure 7F-HFD

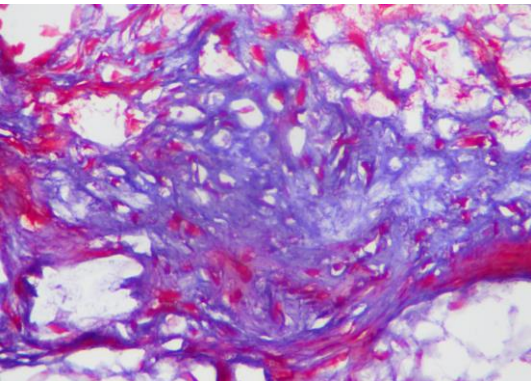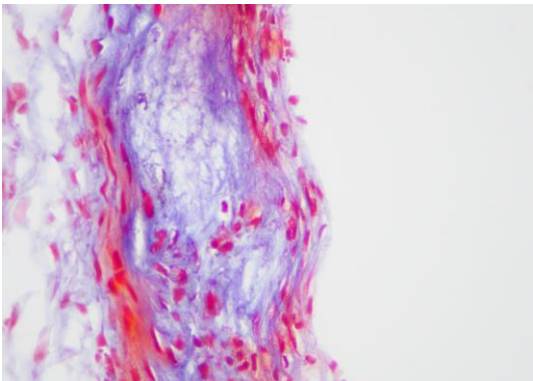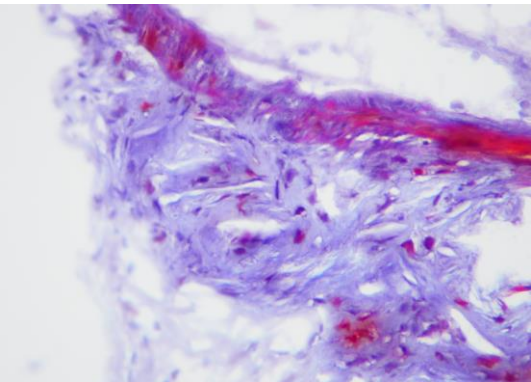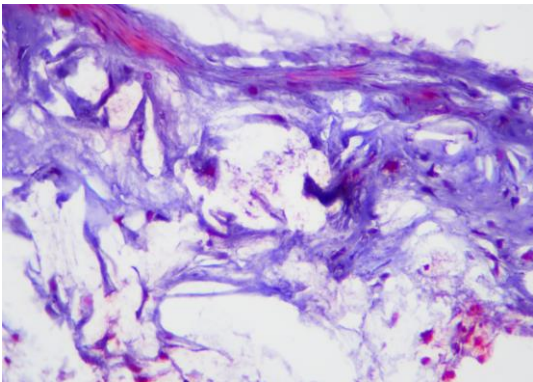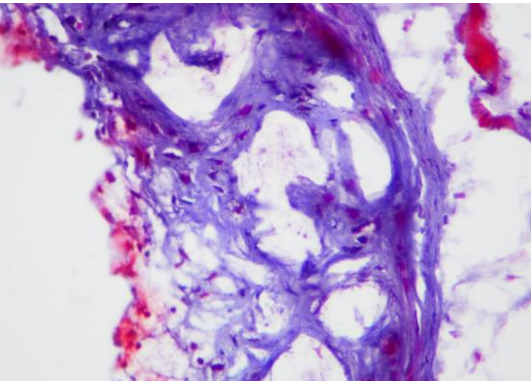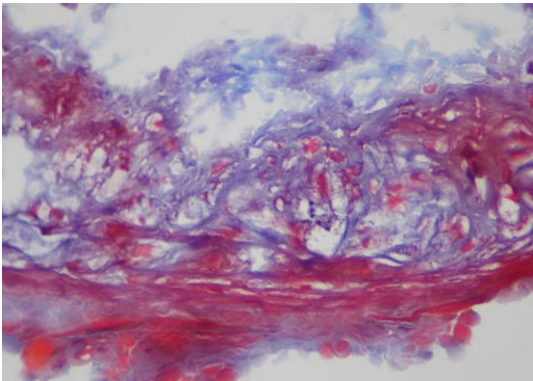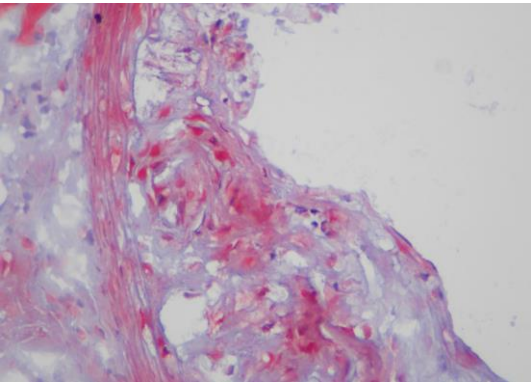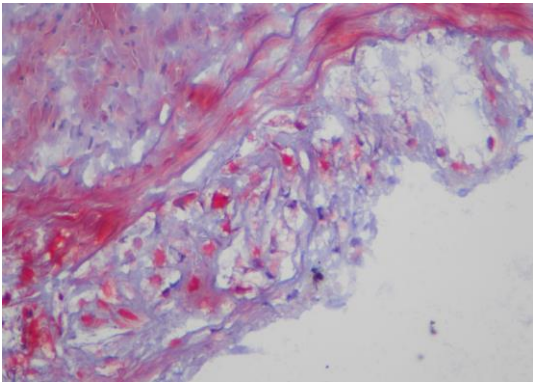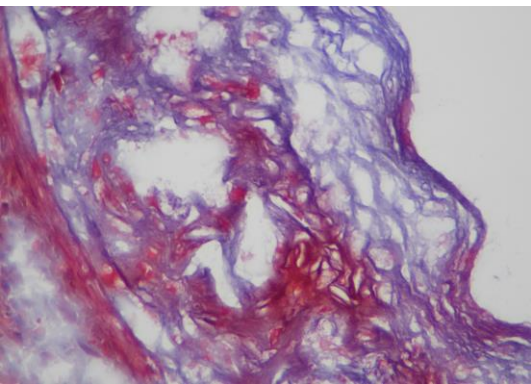

Figure 7F-HFD+DCLK1-IN-1

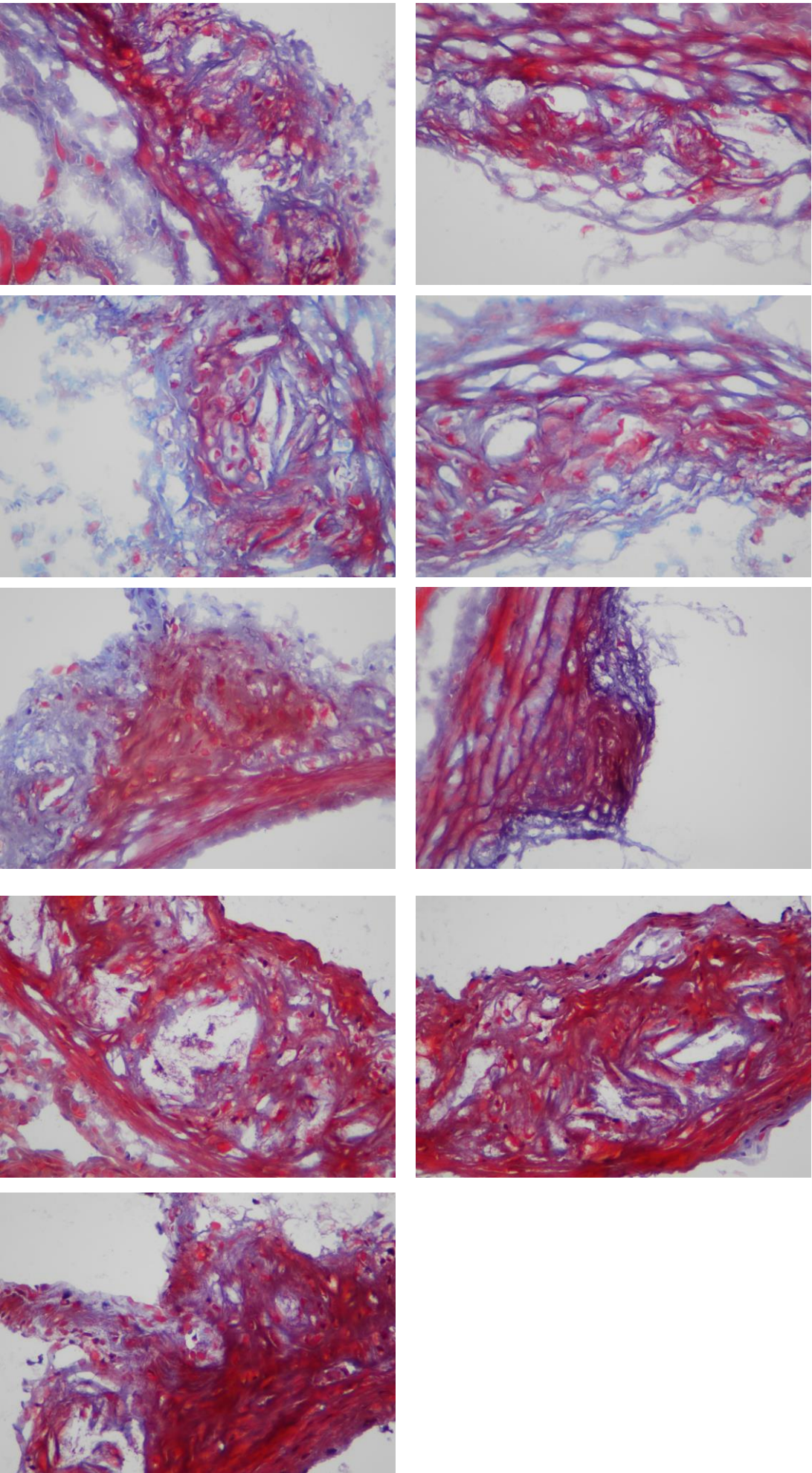

Supplement: Supplementary file 9 — Source Data for Figure 7 [file EMMM-15-e17198-s003.zip › EMM-2022-17198-V2-Figure_7_Source_Data-sd/7F/7F-Masson.pdf]

Figure 7H-LFD

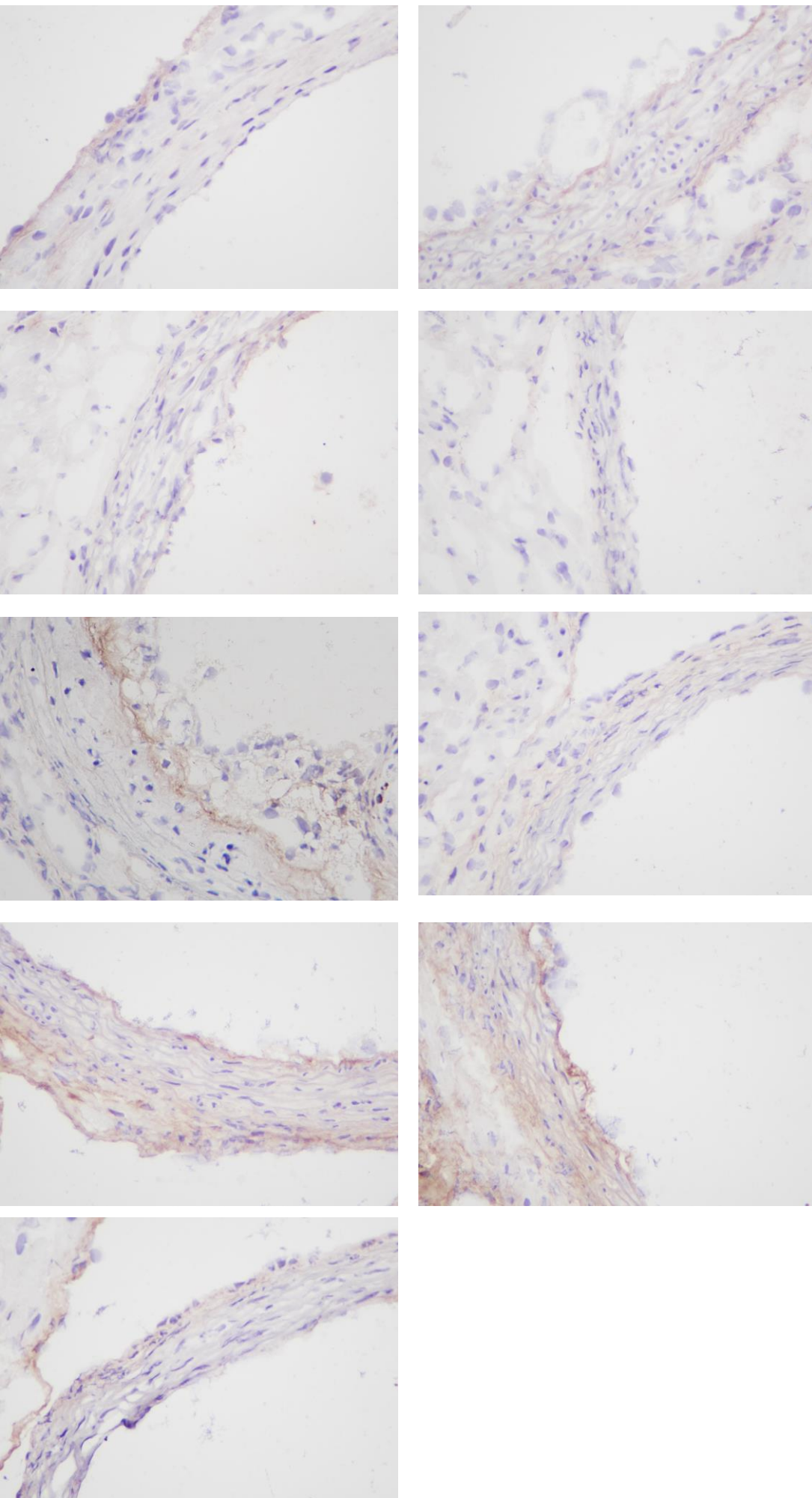

Figure 7H-HFD

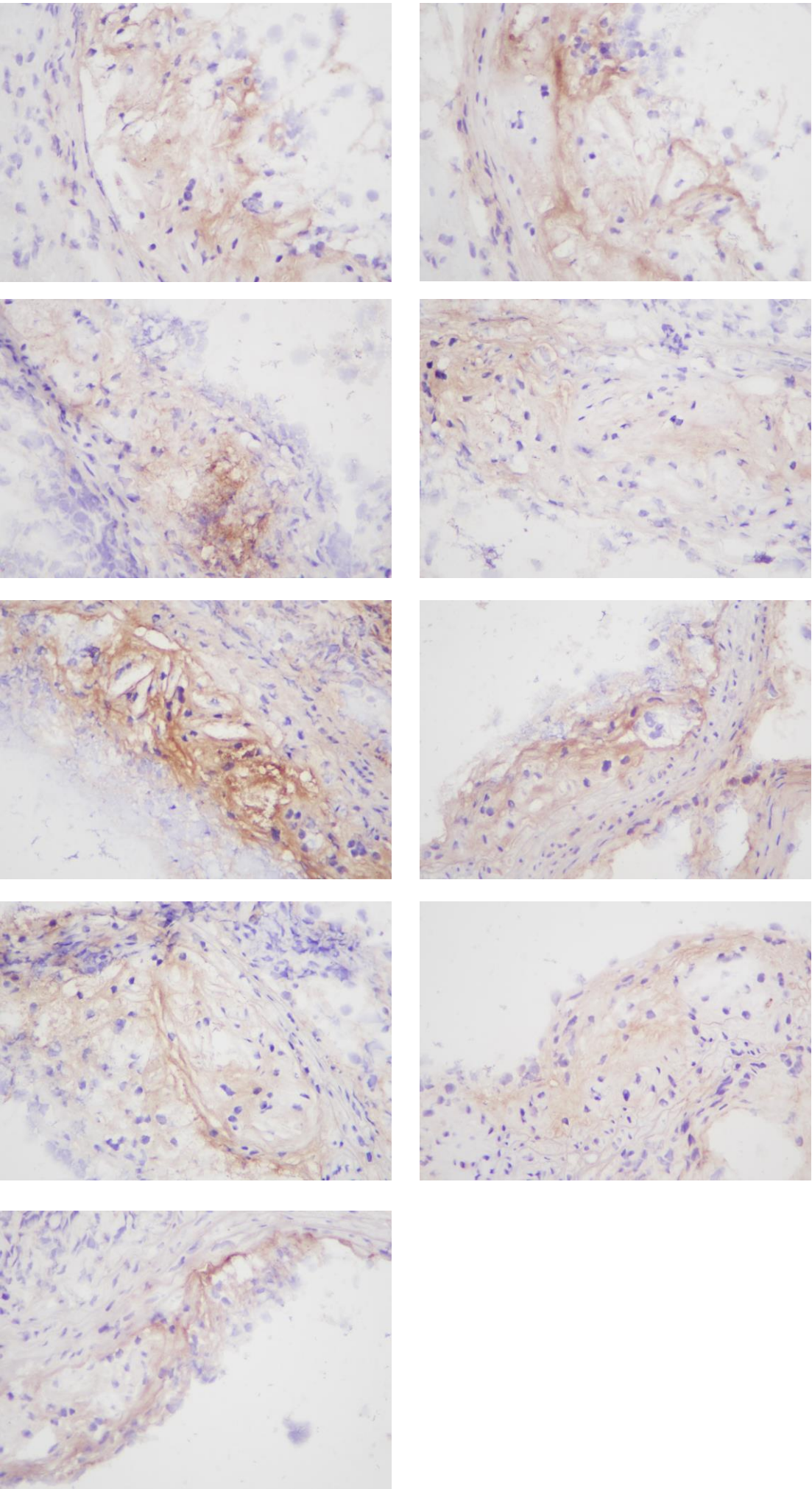

Figure 7H-HFD+DCLK1-IN-1

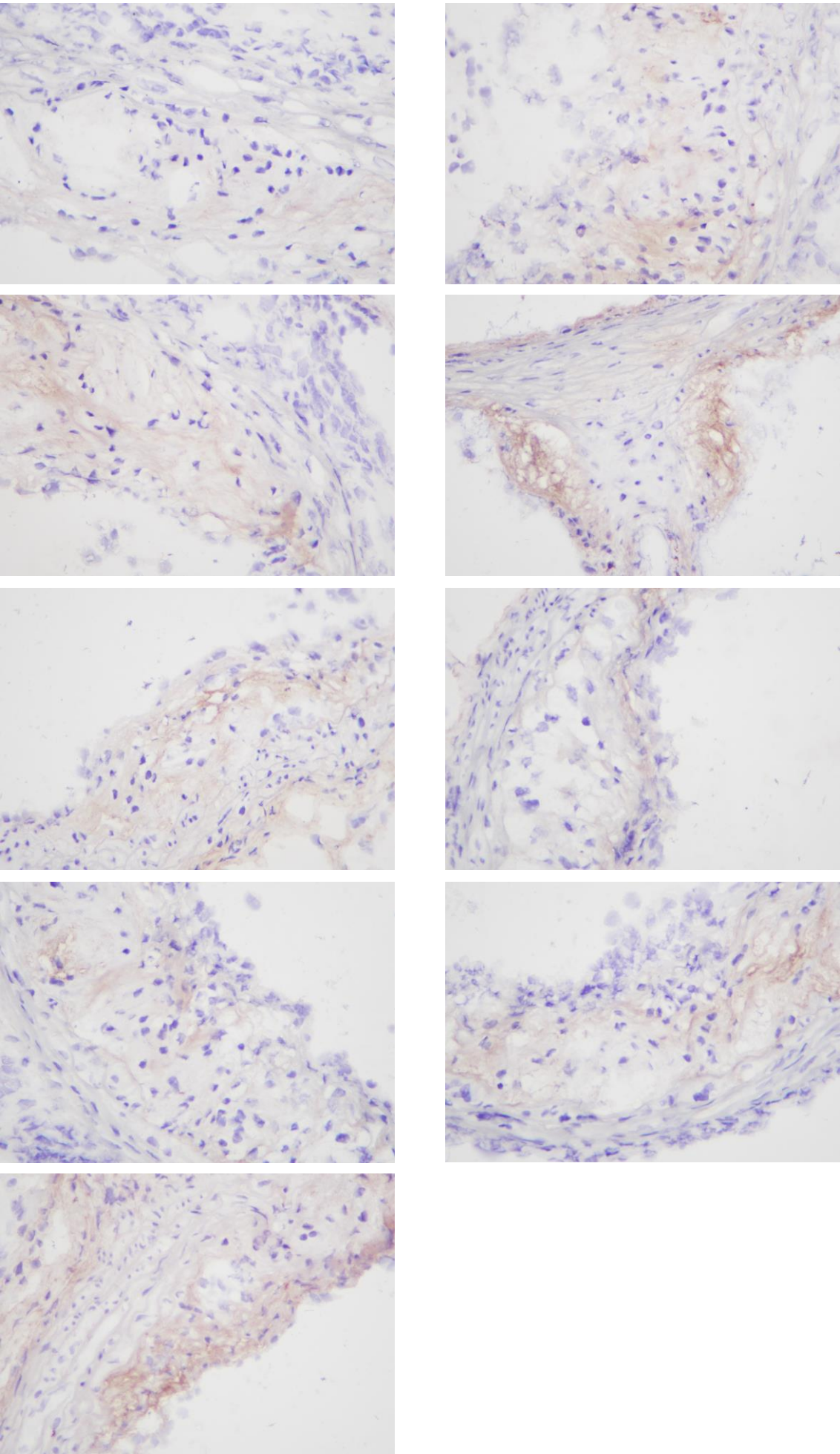

Supplement: Supplementary file 9 — Source Data for Figure 7 [file EMMM-15-e17198-s003.zip › EMM-2022-17198-V2-Figure_7_Source_Data-sd/7H/7H-Ly6G.pdf]
