# Supplementary material for: Macrophage DCLK1 promotes atherosclerosis via binding to IKKβ and inducing inflammatory responses
Source: EMBO Mol Med. 2023 Mar 10;15(5):e17198. doi: 10.15252/emmm.202217198 (PMC10165355; doi:10.15252/emmm.202217198)

Figure 2C-LFD-DCLK1$^{f/f}$

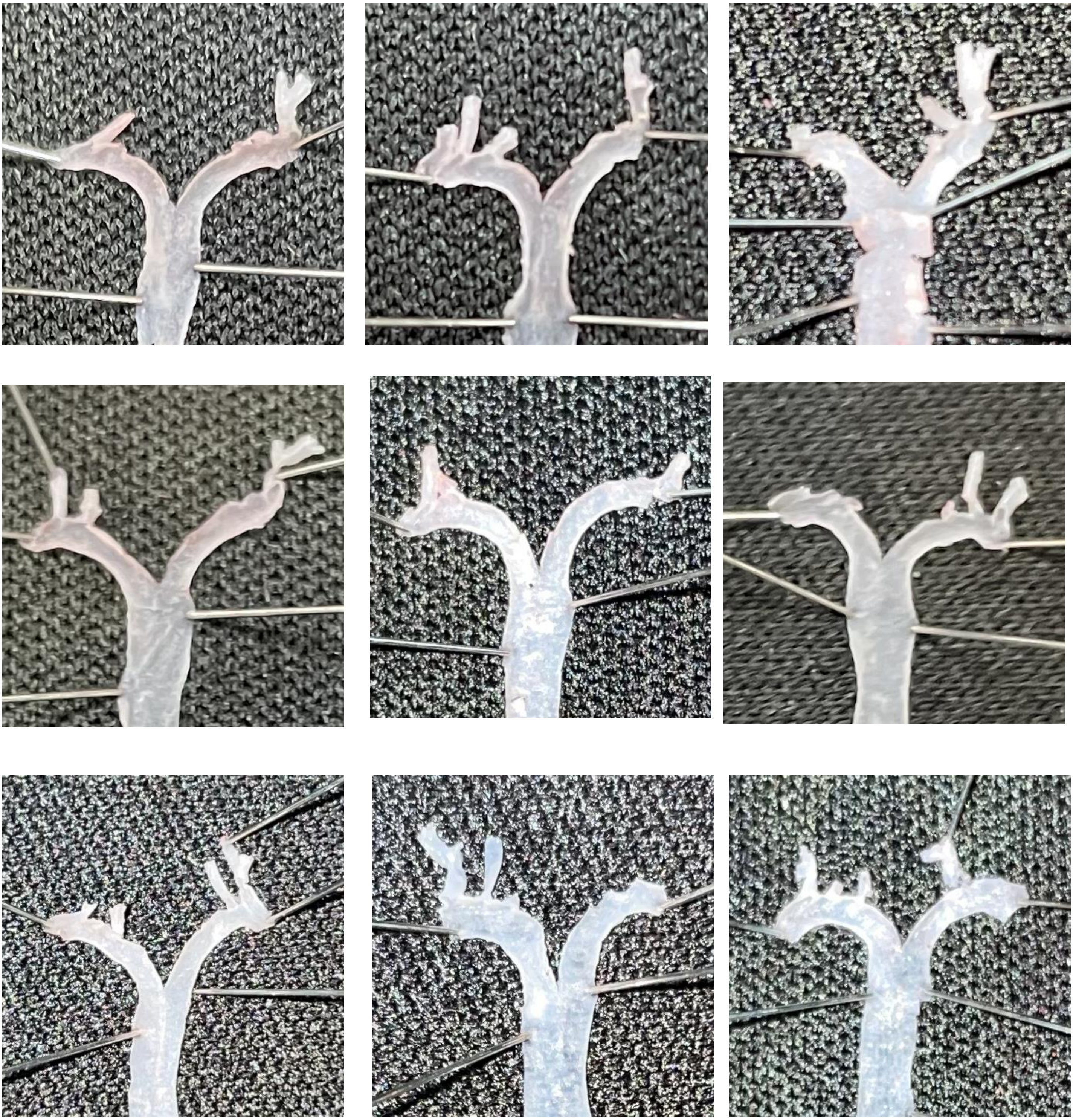

Figure 2C-LFD-DCLK1^{MCKO}

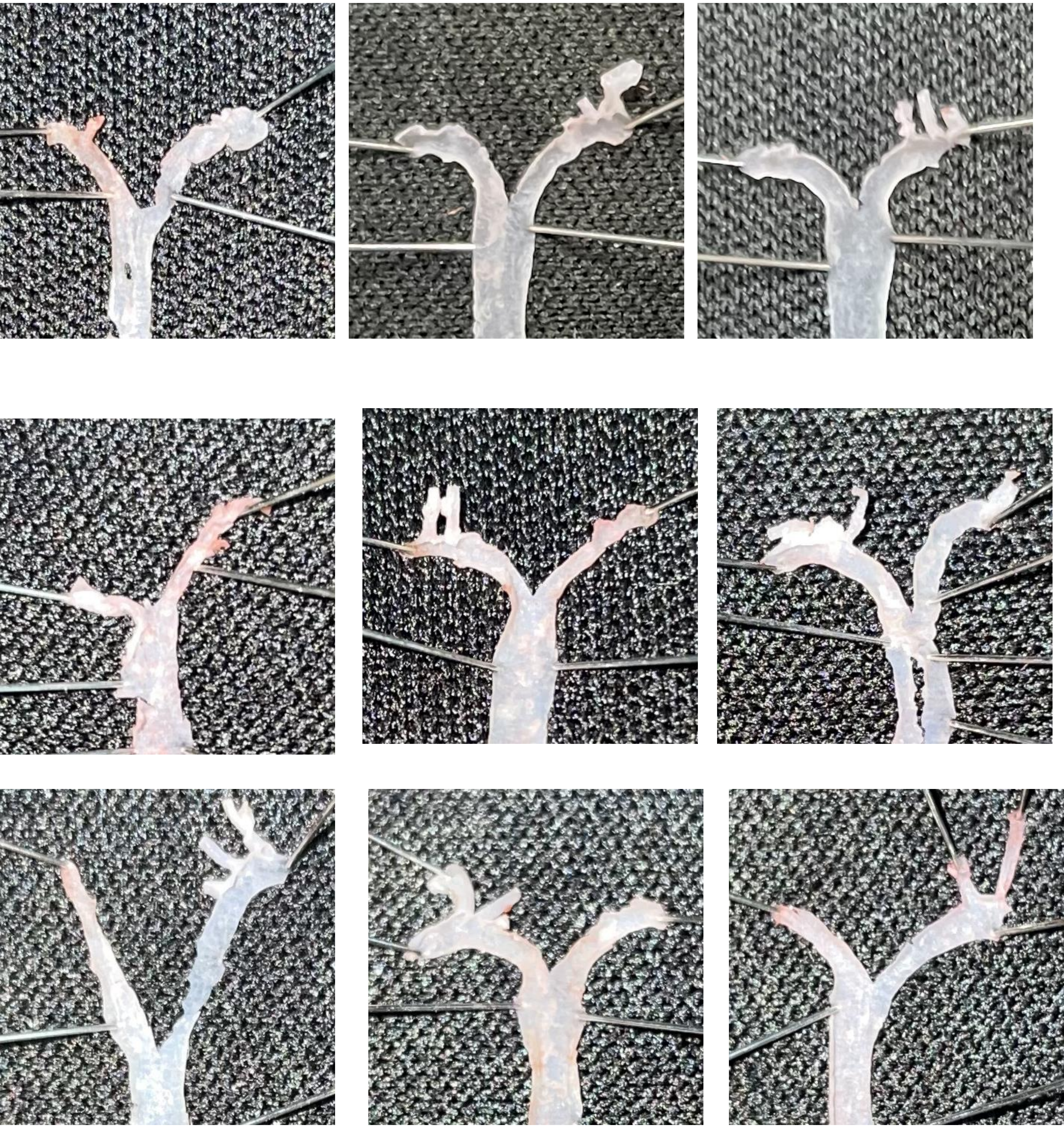

Figure 2C-HFD-DCLK1$^{f/f}$

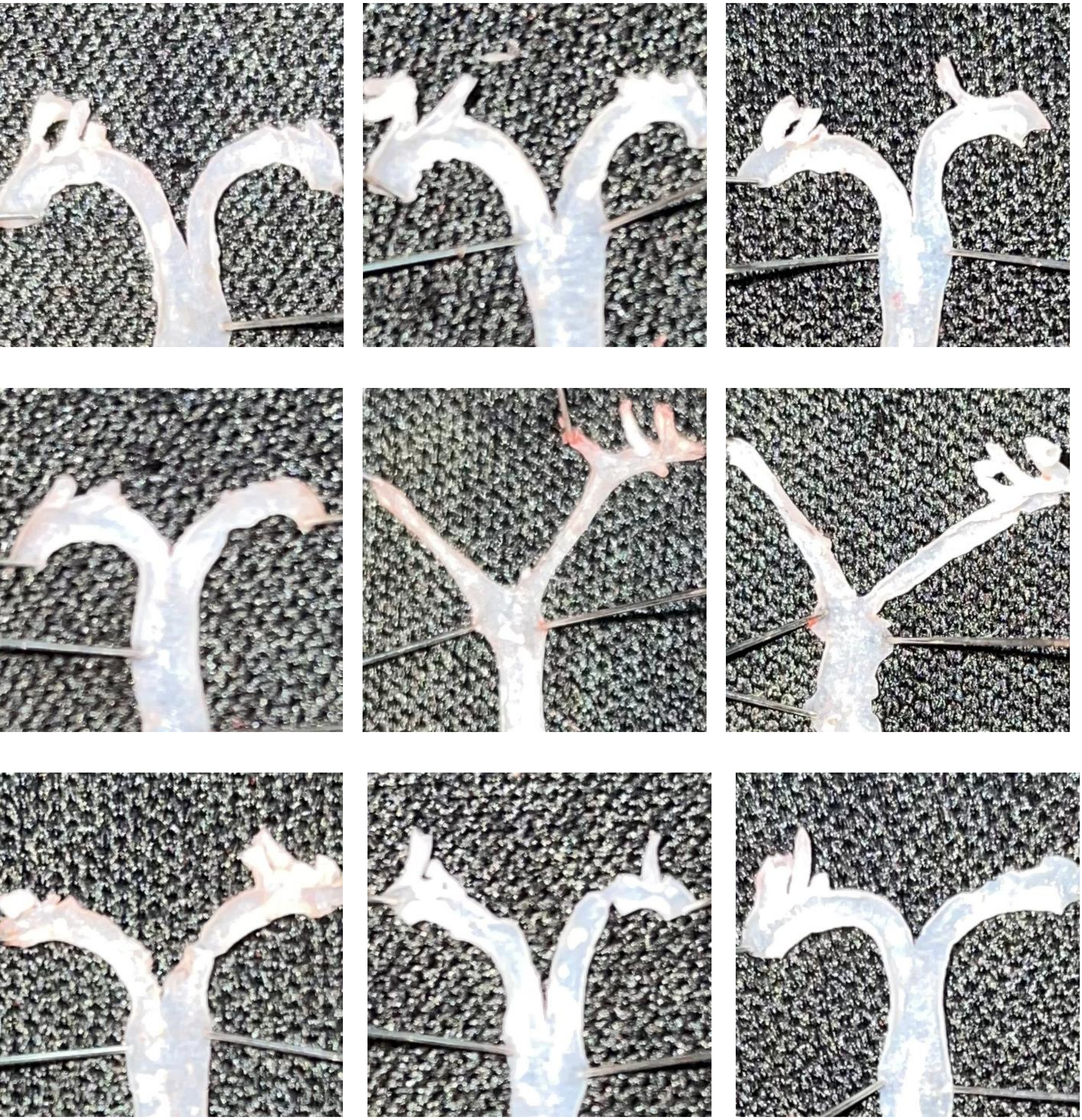

Figure 2C-HFD-DCLK1^{MCKO}

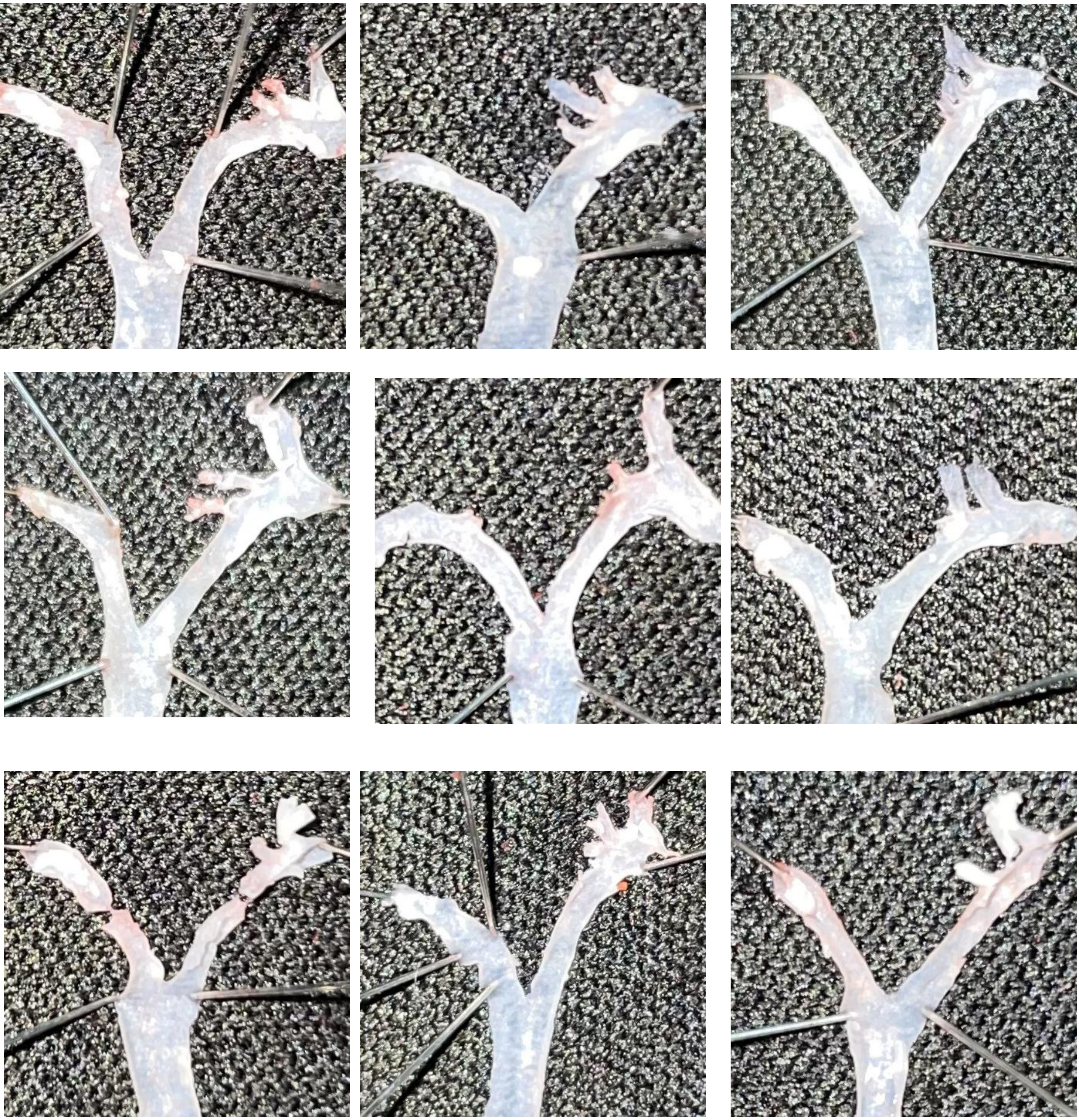

Supplement: Supplementary file 4 — Source Data for Figure 2 [file EMMM-15-e17198-s002.zip › EMM-2022-17198-V2-Figure_2_Source_Data-sd/2C-D/2C-aotic arches.pdf]
