# Supplementary material for: Macrophage DCLK1 promotes atherosclerosis via binding to IKKβ and inducing inflammatory responses
Source: EMBO Mol Med. 2023 Mar 10;15(5):e17198. doi: 10.15252/emmm.202217198 (PMC10165355; doi:10.15252/emmm.202217198)

Figure 4P-LFD-DCLK1$^{f/f}$

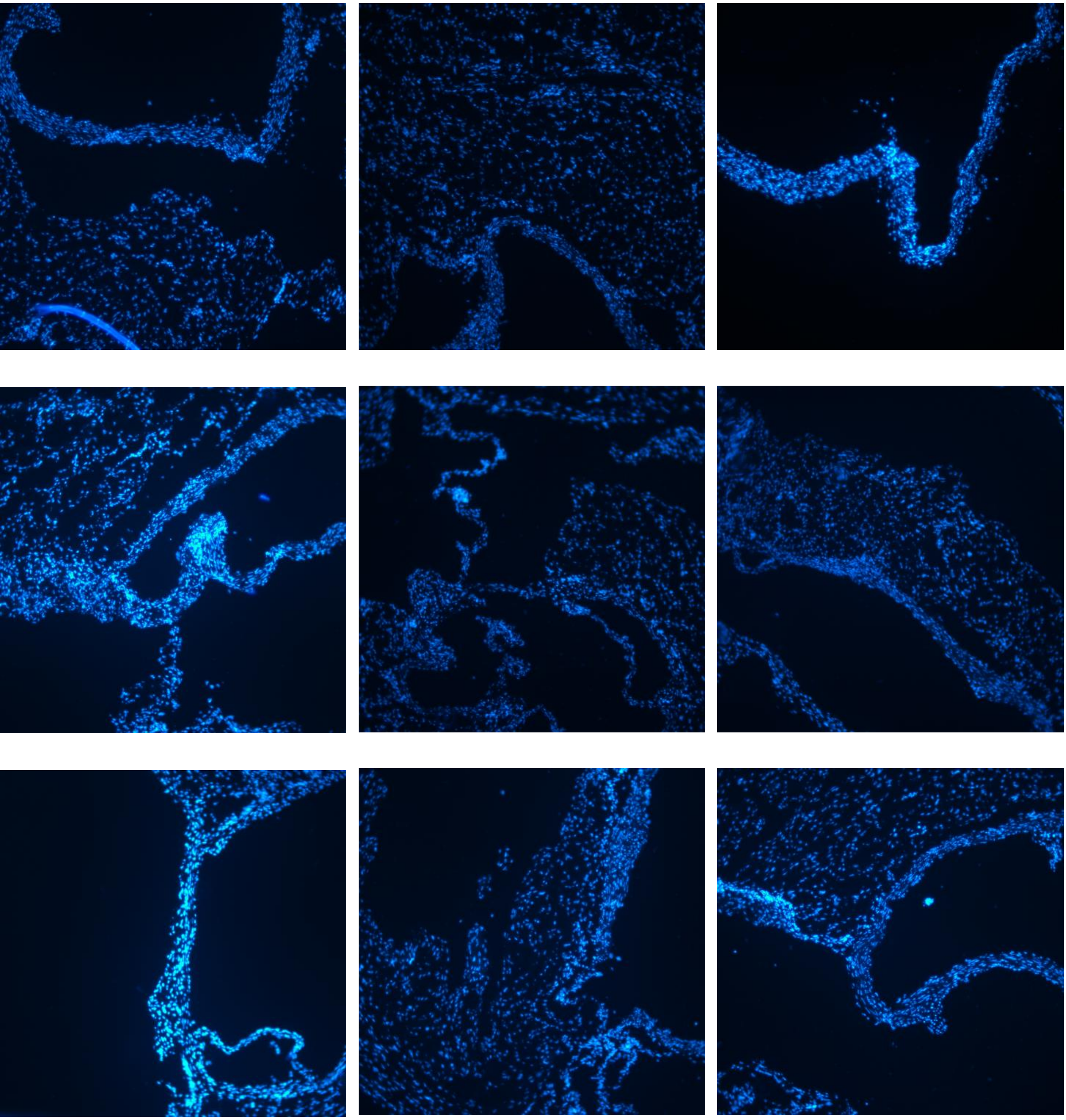

Figure 4P-LFD-DCLK1^{MCKO}

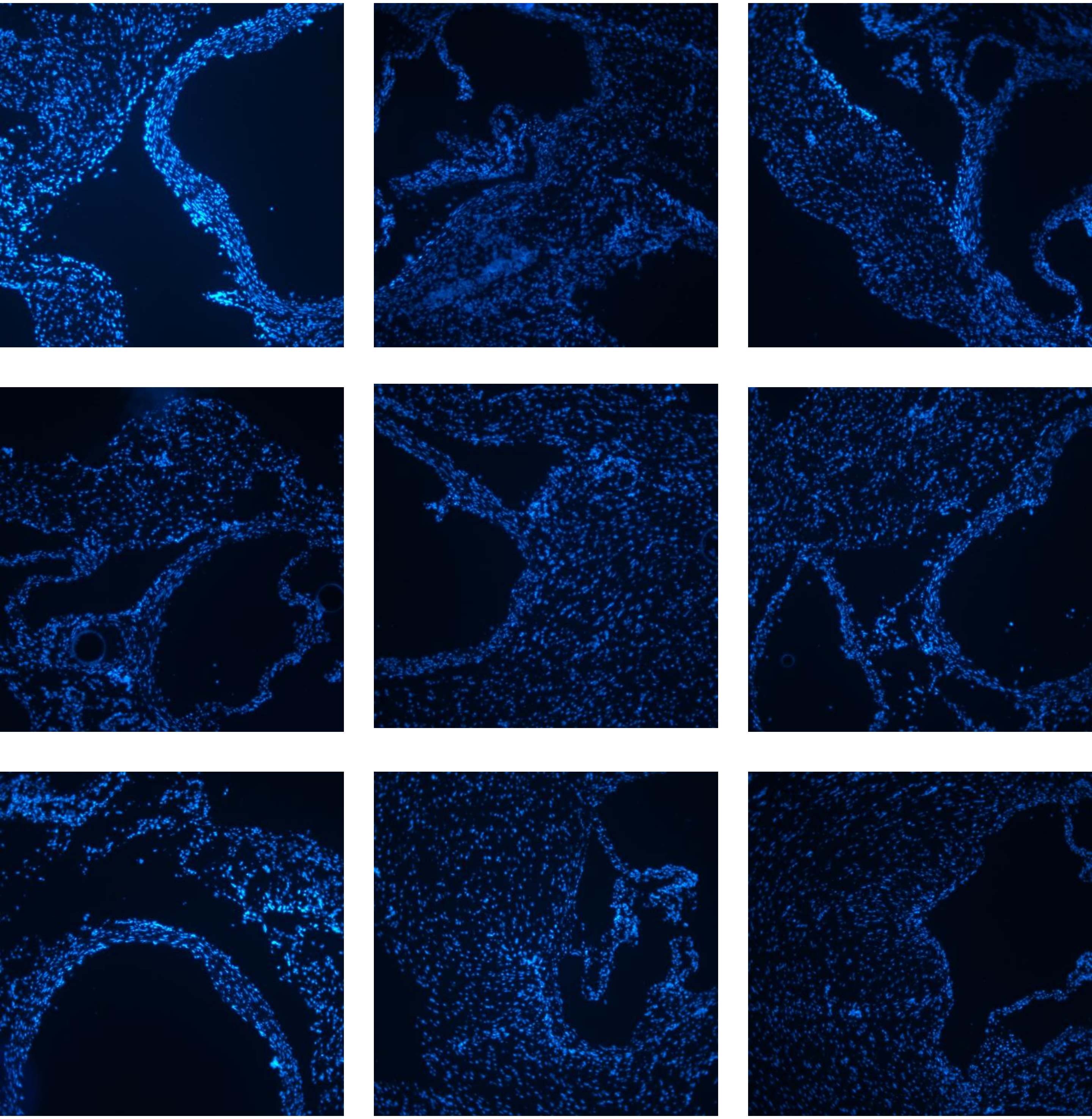

Figure 4P-HFD-DCLK1$^{f/f}$

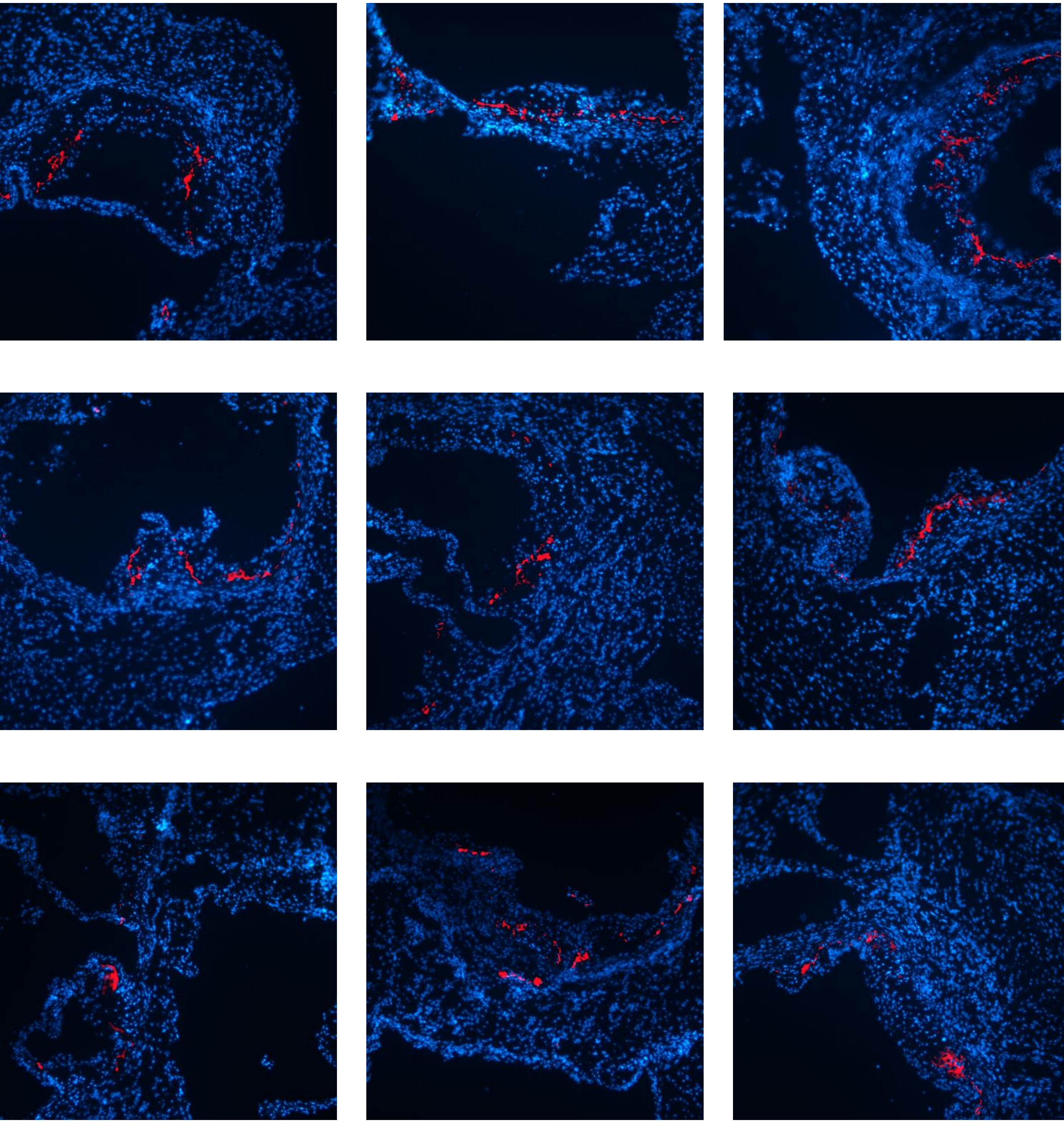

Figure 4P-HFD-DCLK1^{MCKO}

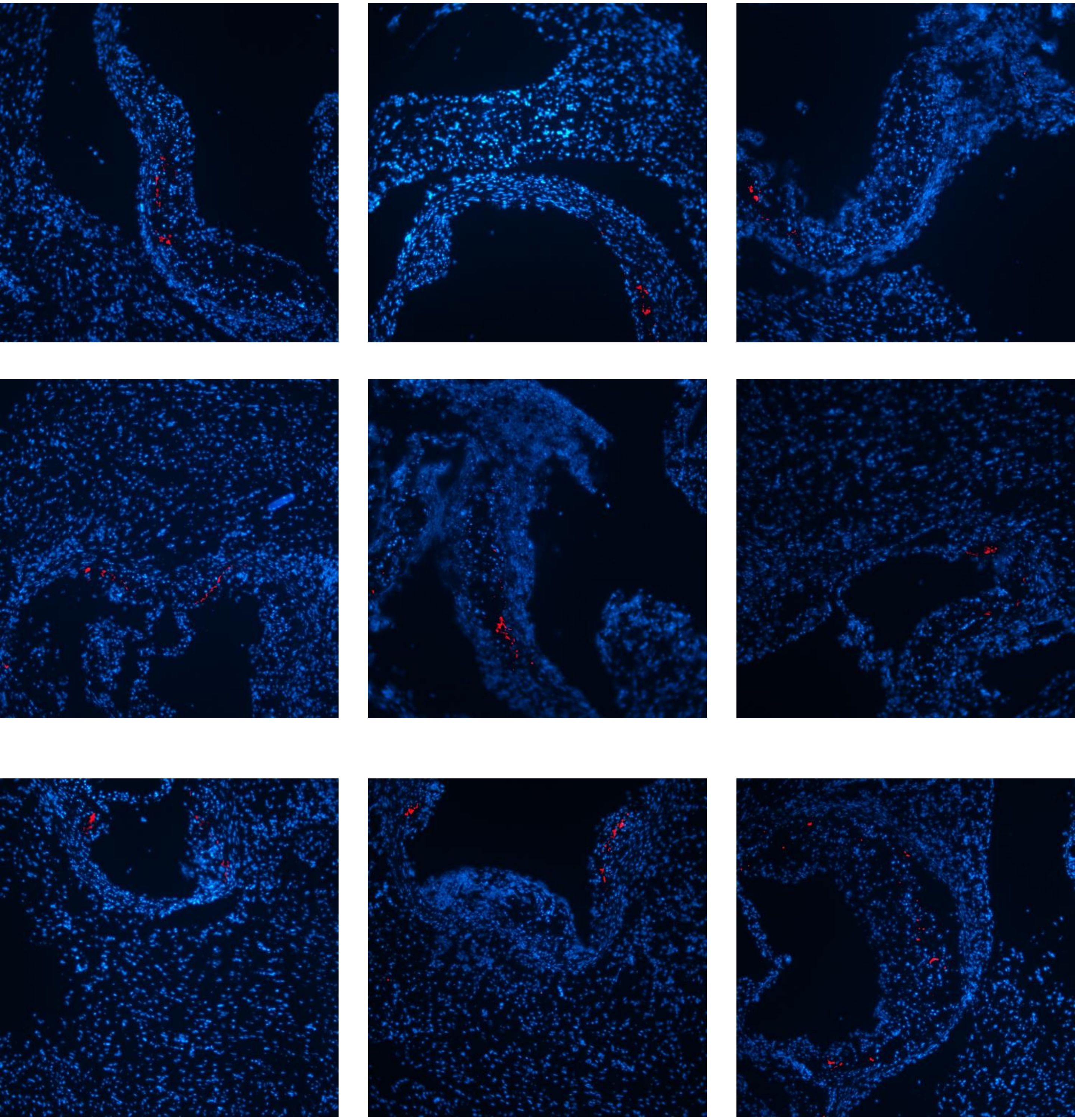

Supplement: Supplementary file 6 — Source Data for Figure 4 [file EMMM-15-e17198-s010.zip › EMM-2022-17198-V2-Figure_4_Source_Data-sd/4P-Q/4P-p65 staining.pdf]
