# Supplementary material for: Macrophage DCLK1 promotes atherosclerosis via binding to IKKβ and inducing inflammatory responses
Source: EMBO Mol Med. 2023 Mar 10;15(5):e17198. doi: 10.15252/emmm.202217198 (PMC10165355; doi:10.15252/emmm.202217198)

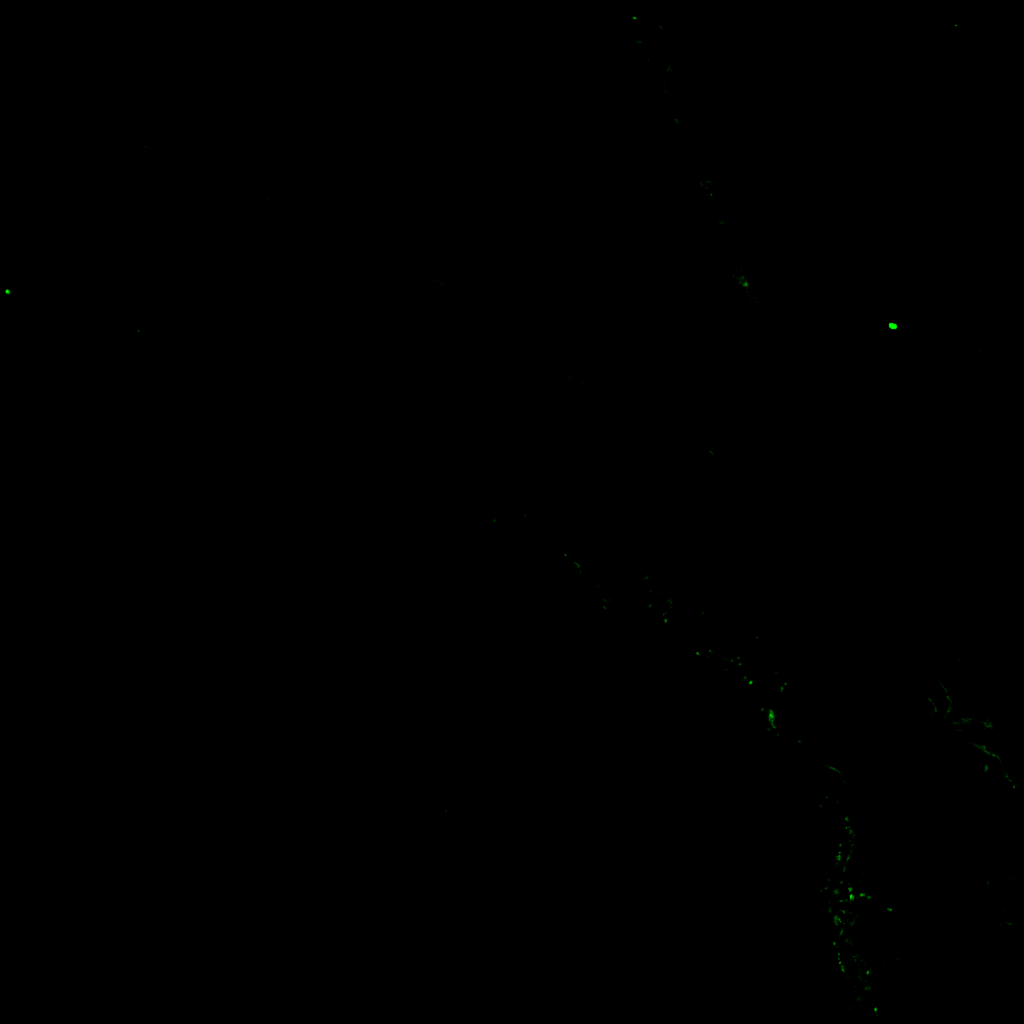

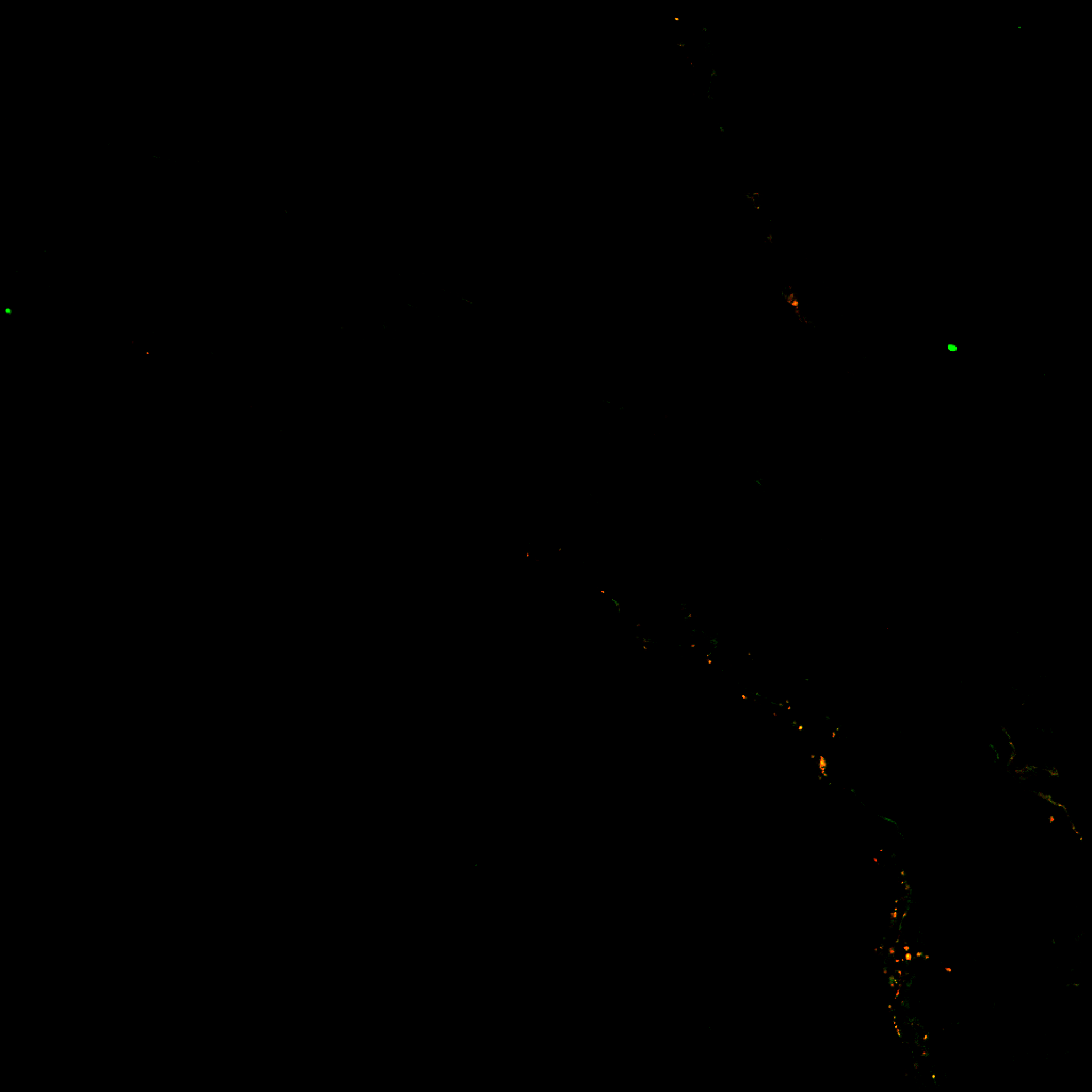

Supplement: Supplementary file 3 — Source Data for Figure 1 [file LFD-F480; DCLK1; Merge.pdf › EMM-2022-17198-V2-Figure_1_Source_Data-sd/1G/LFD-F480; DCLK1; Merge.pdf]
