# Supplementary material for: Macrophage DCLK1 promotes atherosclerosis via binding to IKKβ and inducing inflammatory responses
Source: EMBO Mol Med. 2023 Mar 10;15(5):e17198. doi: 10.15252/emmm.202217198 (PMC10165355; doi:10.15252/emmm.202217198)

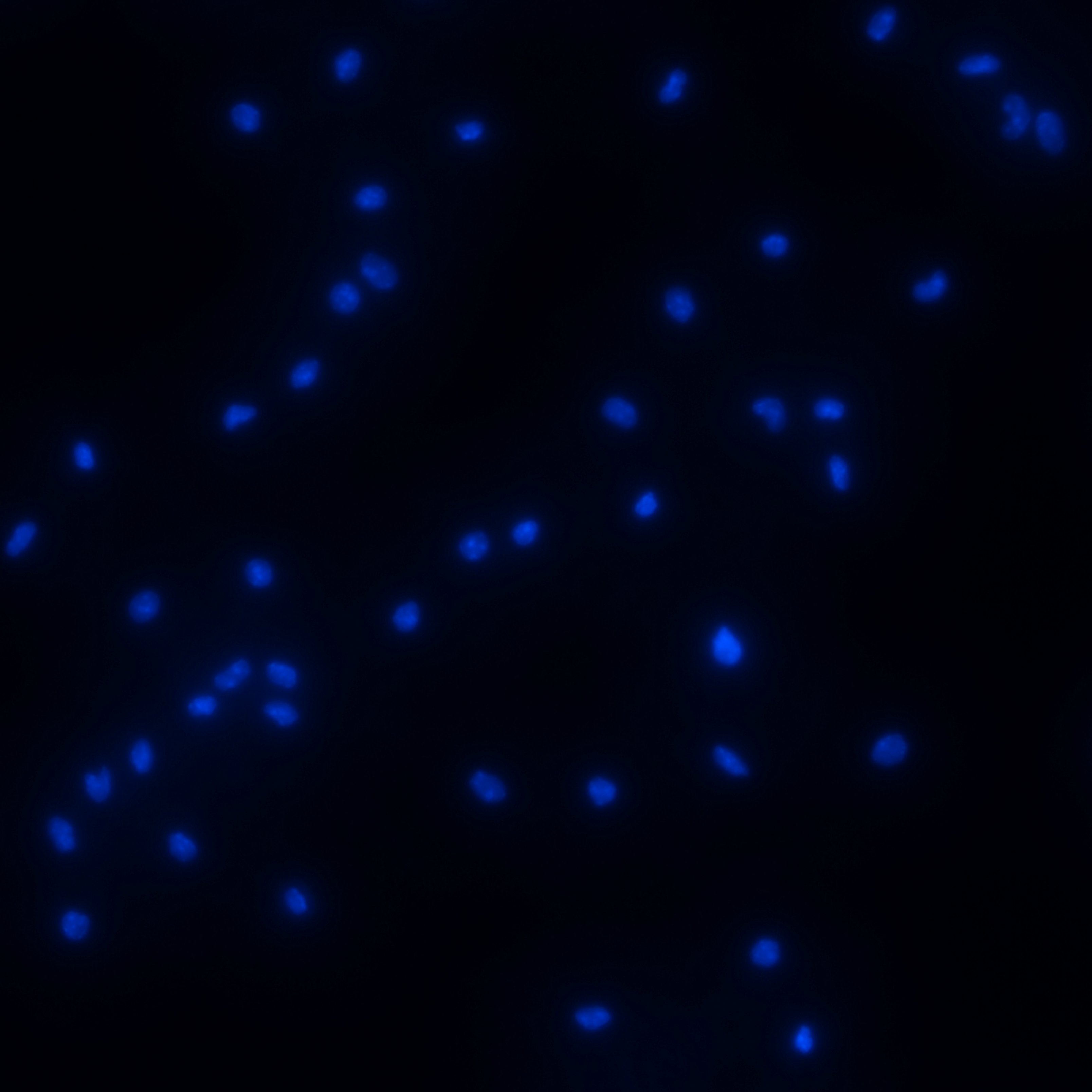

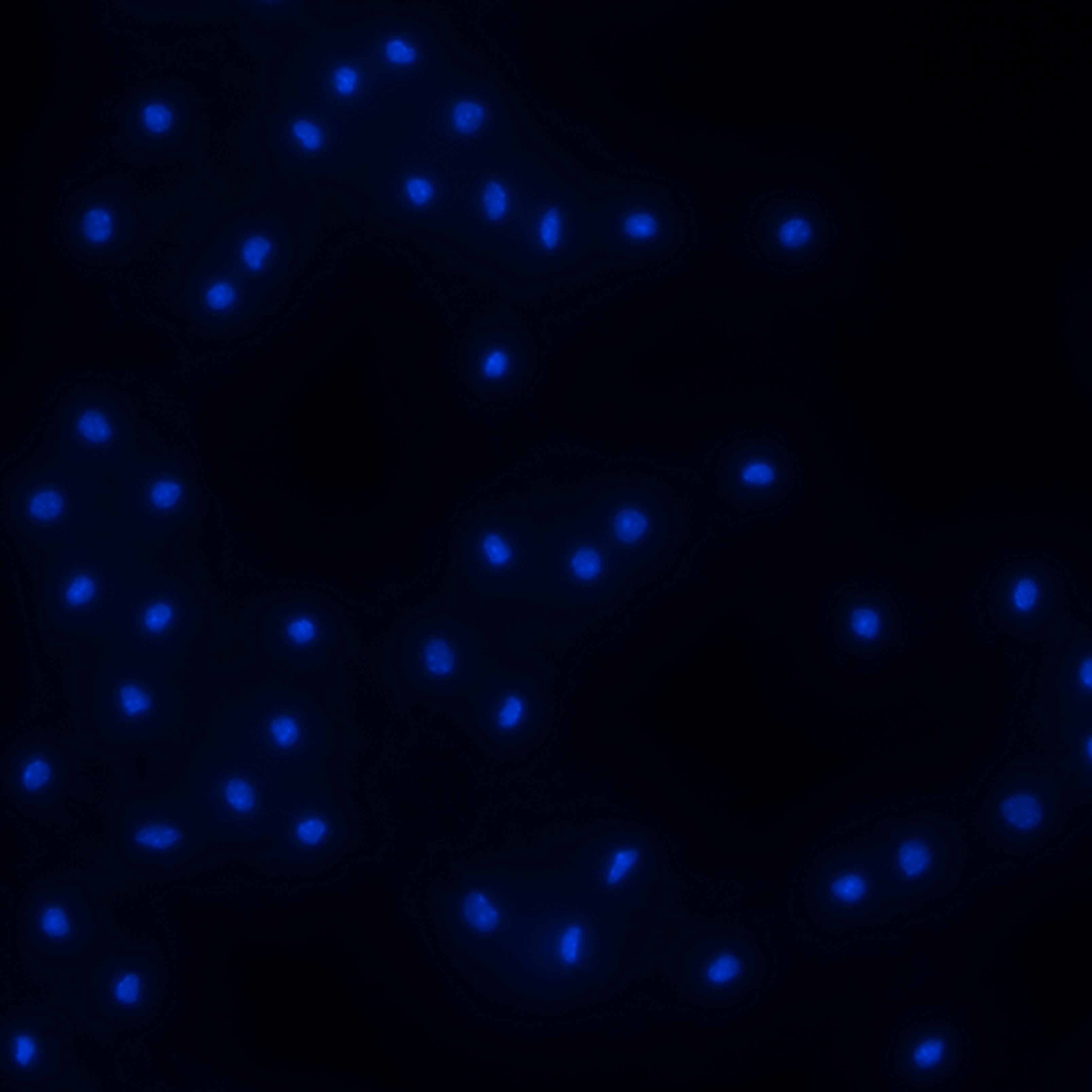

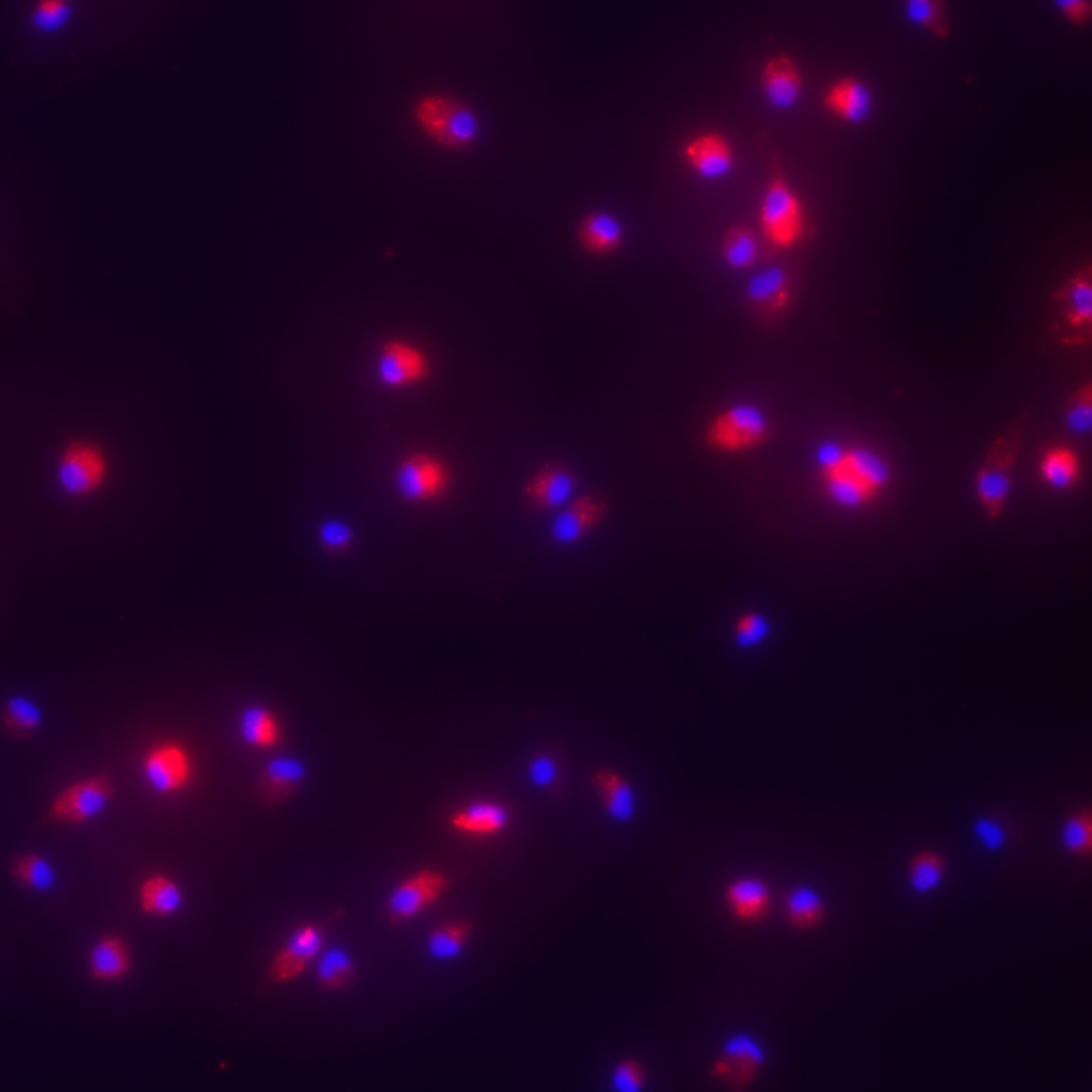

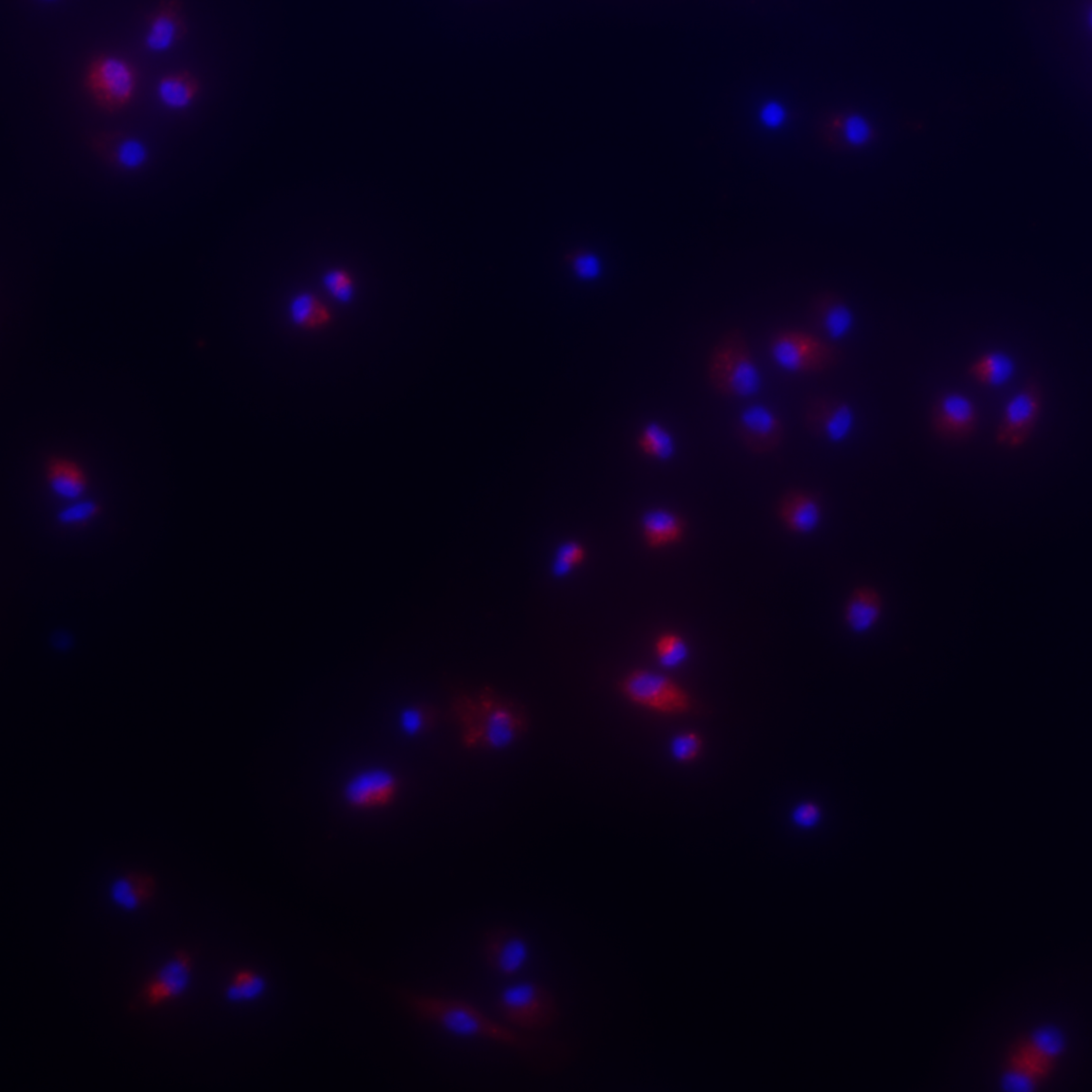

Supplement: Supplementary file 6 — Source Data for Figure 4 [file DiI-oxLDL.pdf › EMM-2022-17198-V2-Figure_4_Source_Data-sd/4E/DiI-oxLDL.pdf]
