# Supplementary material for: Macrophage DCLK1 promotes atherosclerosis via binding to IKKβ and inducing inflammatory responses
Source: EMBO Mol Med. 2023 Mar 10;15(5):e17198. doi: 10.15252/emmm.202217198 (PMC10165355; doi:10.15252/emmm.202217198)
Supplement: Supplementary file 10 [file EMMM-15-e17198-s007.pdf]

EMBO Molecular Medicine

Macrophage DCLK1 promotes atherosclerosis via binding to IKKβ and inducing inflammatory responses

Zhuqi Huang, Sirui Shen, Xue Han, Weixin Li, Wu Luo, Liming Lin, Mingjiang Xu, Yi Wang, Weijian Huang, Gaojun Wu, and Guang Liang
DOI: 10.15252/emmm.202217198

Corresponding author: Guang Liang (wzmcliangguang@163.com)

Review Timeline:

Submission Date:	18th Nov 22
Editorial Decision:	14th Dec 22
Revision Received:	18th Feb 23
Editorial Decision:	21st Feb 23
Revision Received:	22nd Feb 23
Accepted:	23rd Feb 23

Editor: Zeljko Durdevic

Transaction Report:

(Note: With the exception of the correction of typographical or spelling errors that could be a source of ambiguity, letters and reports are not edited. Depending on transfer agreements, referee reports obtained elsewhere may or may not be included in this compilation. Referee reports are anonymous unless the Referee chooses to sign their reports.)

14th Dec 2022

Dear Prof. Liang,

Thank you for the submission of your manuscript to EMBO Molecular Medicine. We have now received feedback from the three reviewers who agreed to evaluate your manuscript. As you will see from the reports below, the referees acknowledge the interest of the study but also raise serious concerns particularly regarding the reproducibility of the findings. The referee #1 is criticizing the small number of animals per experiment making the reproducibility of experiments unclear, referee #2 raises important but minor concerns, while the referee #3 raises a number of concerns particularly regarding antibodies specificity and unsatisfactory quality of histological images and wester blots.

Taking the in consideration it is clear that publication of the paper cannot be considered at this stage. After our cross-commenting session it became clear that the number of animals per experiment should be increased to at least 10, specificity of the antibodies should be confirmed, quantification methods for western blots and histological images should be better explained and complete images in high quality (uncropped western blots and full histological images, not only magnifications) should be provided for further consideration of the manuscript. I also note that addressing these aspects and the reviewers concerns in full appears to require a lot of additional work and experimentation. I am unsure whether you will be able or willing to address those and return a revised manuscript within the 6 months deadline. If you would like to discuss further the points raised by the referees, I am available to do so via email or video. Let me know if you are interested in this option.

Please note that further consideration of a revision that addresses reviewers' concerns in full will entail a second round of review. EMBO Molecular Medicine encourages a single round of revision only and therefore, acceptance or rejection of the manuscript will depend on the completeness of your responses included in the next, final version of the manuscript. For this reason, and to save you from any frustrations in the end, I would strongly advise against returning an incomplete revision and would also understand your decision if you chose to rather seek rapid publication elsewhere at this stage.

Should you find that the requested revisions are not feasible within the constraints outlined here and choose, therefore, to submit your paper elsewhere, we would welcome a message to this effect.

Yours sincerely,

Zeljko Durdevic

Zeljko Durdevic
Editor
EMBO Molecular Medicine

***** Reviewer's comments *****

Referee #1 (Remarks for Author):

Huang et al. identified macrophage DCLK1 as a regulator of inflammation in atherosclerosis. Mechanistically, DCLK1 phosphorylates IKKβ and thereby facilitates inflammatory NF-kappaB signaling. Interestingly, both the macrophage-specific KO as well as systemic inhibition with a small molecule inhibitor markedly alleviated atherosclerotic plaque burden. The strength of the paper is its novelty - DCLK1 has not been implicated in atherosclerosis so far - and the potential use of DCLK1 as a therapeutic target in atherosclerosis. The weaknesses are that the reproducibility of some of animal experiments are unclear. My major and minor points of criticism are listed below:

Major:

1. The atherosclerosis studies include an unusual small number of mice. How was the group size and statististical power calculated? How were mice included/excluded?
2. The authors demonstrate that DCLK1 mediates inflammation in macrophages via Nf-KB by phosphorylation of IKKβ. However, how this translate to a reduction in atherosclerotic plaques is insufficiently explained. I would assume that the KO macrophages are pushed towards a less inflammatory/infiltrating phenotype, that also secret less pro-inflammatory cytokines, which the authors also mention in the discussion. To make this claim, the authors should provide a more detailed characterization of the macrophage polarization type and the inflammatory milieu besides TNF and IL-6 level.

Minor:
3. The pictures in the manuscript have very low quality and should be replaced with a higher resolution. The authors should also provide pictures of en face stained aortas from all animals in the supplementary material. Also, the color code for the fluoresecent pictures is inappropriate (blue on black)
4. I respectfully point out that the language of the paper needs to be improved, as the authors have an unusual choice of verbs and there are missing words and grammatical errors.
5. The authors conclude in figure 2 that MCKO DCLK1 alleviates HFD-induced atherosclerotic development without affecting lipid metabolism. However, the authors do not did not provide any data on lipid metabolism besides blood lipid levels. Therefore, they should refer only to blood lipids.

Referee #2 (Comments on Novelty/Model System for Author):

The authors provide a comprehensive study, which looks technically sound - including all necessary controls.
The role of DCLK1 in atherosclerosis is novel. So far it cannot be judged, whether it has a high medical impact (as human data are not yet shown). The mouse model is adequat - and professional.

Referee #2 (Remarks for Author):

This study describes a proinflammatory role of the kinase DCLK1 in macrophages - and based on that a role in development of atherosclerosis in mice. It demonstrates that DCLK1 is upregulated in atherosclerotic plaques dependent on high fat diet (HFD) and oxLDL and that a macrophage-specific deletion of this gene attenuates atherosclerosis in HFD-fed ApoE knockout mice. Data shown by the authors indicate that macrophage-deletion of DCLK1 reduces infiltration of monocytes and neutrophils into the plaques. RNA-sequencing of macrophages derived from DCLK1-KO and control mice and treated with OxLDL revealed a significant downregulation of the NF-κB pathway (as shown by GSEA, as well as Western blot analysis of p65 phosphorylation and IKB degradation). Immunoprecipitation of flag-tagged DCLK1 overexpressed in 293 cells, followed by MS-based identification of bound proteins revealed IKKβ as binding partner. The authors then show that DCLK1 can phosphorylate the serine residues of the IKKβ activation loop. Pharmacological inhibition of DCLK1 resulted in a reduction of IKKβ activation, p65 phosphorylation and IKB degradation in vitro and also attenuated atherosclerosis in vivo.
Altogether the manuscript presents a comprehensive study with all the required controls.
There are just some points left to be clarified:

• Fig. 1D: The authors should specify in more detail, how quantitative PCR was done (which method was used to calculate fold induction, has the PCR efficiency been considered?)
• Fig. 3G: percentages of the various leukocyte subtypes should be shown in the scattergrams.
• Fig. 4H: The p-value color coding should be changed to obtain a better visualization (e.g., by setting 0.0 as red and 0.05 as blue (instead of 1.00). Alternatively, the bars might be labeled with the numerical p-value.
• Fig. 4P: Nuclear translocation is not obvious from the images. Images should be shown without the DAPI channel for better assessment of p65 nuclear translocation. The authors should specify in more detail how nuclear translocation was quantified. Ideally, nuclear/cytosol ratios should be determined for the cells and statistics calculated.

Referee #3 (Comments on Novelty/Model System for Author):

This is overall an impressive manuscript with novel and clinically potentially important data. I have listed some concerns/suggestions which the authors should be able to address.

Referee #3 (Remarks for Author):

Atherosclerosis is a chronic inflammatory disease that results in the formation of immune cell-rich plaques in the arterial wall, leading to high morbidity and mortality in the world. Inflammation is a process that plays an important role in the initiation and progression of atherosclerosis, involving multiple cell types, such as macrophages, T cells and other cells of the immune response.

The manuscript by Huang et al. focuses on the role of doublecortin-like serine/threonine kinase 1 (DCLK1) in inflammatory diseases, such as atherosclerosis. Recent studies have indicated a role of the microtubule-associated protein DCLK1 as a tumor stem cell marker as well as in neurogenesis and neuronal migration. So far, the function of DCLK1 in atherosclerosis has not been defined yet.

To determine if DCLK1 plays a role in this cardiovascular disease, Huang et al. investigated and reported up-regulated DCLK1 levels in macrophages of ApoE-/- mice fed a HFD and suggest that macrophage-specific DCLK1 deletion attenuates atherosclerosis by reducing inflammation in mice. Implemented RNA sequencing analysis indicated that DCLK1 could mediate oxLDL-induced inflammation via NF-κB signaling pathway in primary macrophages. Co-immunoprecipitation followed by LC-MS/MS analysis suggests IKKβ as a binding protein of DCLK1.

The three claimed key findings of the present study are as follows: (1) Macrophage-specific deletion or pharmacological inhibition of DCLK1 attenuates atherosclerotic progression; (2) macrophage-specific deletion or pharmacological inhibition of DCLK1 alleviates chronic inflammation by inhibiting NF-κB activation; (3) DCLK1 directly binds to IKKβ and phosphorylates IKKβ at S177/181.

Overall, the manuscript is well written, the results are well presented and discussed appropriately. The suggestions of DCLK1 as a new IKKβ regulator in inflammation and a potential therapeutic target for inflammatory atherosclerosis are very interesting and the novelty is very high since a correlation to atherosclerosis has not been shown in the literature yet. However, the study comprises some limitations. The following concerns and suggestions might help the authors to improve their manuscript:

Comments
- Introduction: In the introduction the authors state to have been engaged in finding new inflammatory mechanisms and targets for "many years" while citing two recent publications from 2020 and 2022. I would argue that this sentence should not be

included in the introduction as it does not help to introduce the topic or the study.

- Figure 1B shows a very tightly framed band for Western blot results and is cut off very close to the bands shown. We suggest that the authors provide entire Western blots.

- Figure 2E and 7E: Most examples of plaque in the sinus shown do not present intact sinus valves and hence cannot be used for comparisons. Here better sections and images would be helpful.

- Figure 2G, Figure 3C-E, Figure 7F-J: Shown extracts of the tissue samples are too small to validate the staining. The staining of, e.g., Ly6G, and Ly6C, shows a lot of background information. The authors need to demonstrate the specificity of the antibodies used with the chosen staining methods and the respective negative (e.g., isotype controls)?

- Figure 4P: The representative immunofluorescence stainings of p65 do not show clear differences. How was the quantification in Figure 4Q implemented? Can the authors provide proof of the specificity of the antibodies used in the respective staining procedures (negative controls, isotype controls, positive tissues). The same concern and consequent request apply to Figure 3A, and Figure 7J)?

- Figure 7C: The representative examples shown do not appear to be less than 5% as stated in the quantification in Figure 7D. In addition, the pictures represent different illumination between 3 examples given and do not provide a qualitative source for quantification.

- Figure 7A: The authors should provide the original pictures since the background of some of the pictures, e.g., Figure 7A, seem "really black". This would be helpful to convince the reviewer and the readers that the post-processing has not changed the quantification.

- Discussion: A schematic representation/ graphic abstract of the main findings is mentioned in the first paragraph of the Discussion. This graphic overview could not be found by this reviewer.

Response to Reviewers

Title: Macrophage DCLK1 promotes atherosclerosis via binding to IKKβ and inducing inflammatory responses
Journal: *EMBO Molecular Medicine*
Submission ID: EMM-2022-17198

Reviewer #1:

Synopsis: Huang et al. identified macrophage DCLK1 as a regulator of inflammation in atherosclerosis. Mechanistically, DCLK1 phosphorylates IKKβ and thereby facilitates inflammatory NF-kappaB signaling. Interestingly, both the macrophage-specific KO as well as systemic inhibition with a small molecule inhibitor markedly alleviated atherosclerotic plaque burden. The strength of the paper is its novelty - DCLK1 has not been implicated in atherosclerosis so far - and the potential use of DCLK1 as a therapeutic target in atherosclerosis. The weaknesses are that the reproducibility of some of animal experiments are unclear. My major and minor points of criticism are listed below:
Response: Thank you for your positive comments. We substantially revised the manuscript according to your suggestions point-by-point.

Reviewer #1-1
The atherosclerosis studies include an unusual small number of mice. How was the group size and statistical power calculated? How were mice included/excluded?
Response: We are sorry for this statistical confusion in the animal experiments. Actually, we utilized 10 mice per group in the animal experiments. Given that we need to use male eight-week-old mice with triple gene-editing ($ApoE^{-/-}DCLK1\text{-}Lyz^{Cre}$ and $ApoE^{-/-}DCLK1^{f/f}$), a limited number of mice could be obtained from one breeding batch. Therefore, we performed animal experiments using mice from two breeding batches (5 + 5 per group). In the original manuscript, we just showed the data and statistical points from one-batch mice to ensure consistency. According to the comment, we performed the additional examinations and increased the number of animals per group to 10 through using all mice from both batches together. We performed statistics using two-tailed unpaired Student's t-test and described this information in the 'Materials and Methods' section. In addition, male eight-week-old $ApoE^{-/-}DCLK1^{f/f}$ and $ApoE^{-/-}DCLK1^{MCKO}$ mice were included in our animal experiments after genotyping validation using PCR, while other mice were excluded. LFD and HFD groups were assigned in a randomized fashion. These information have been updated in the 'Materials and Methods' section.

Reviewer #1-2
The authors demonstrate that DCLK1 mediates inflammation in macrophages via Nf-KB by phosphorylation of IKKβ. However, how this translate to a reduction in atherosclerotic plaques is insufficiently explained. I would assume that the KO macrophages are pushed towards a less inflammatory/infiltrating phenotype, that also secret less pro-inflammatory

cytokines, which the authors also mention in the discussion. To make this claim, the authors should provide a more detailed characterization of the macrophage polarization type and the inflammatory milieu besides TNF and IL-6 level.

Response: Thanks for these suggestions. In atherosclerosis, infiltrated and activated macrophages secrete pro-inflammatory chemokines and cytokines that in turn facilitate further recruitment of inflammatory cells (neutrophils, monocytes, and macrophages) into the lesion area (Circ Res. 2019, 124(2): 315-327; Nat Rev Cardiol. 2019, 16(7): 389-406). These inflammatory cells promote plaque growth and make plaques become more advanced (Circ Res. 2022, 130(1): 130-148; Nat Rev Cardiol. 2022, 19(8): 522-542). IKKβ/NF-κB signaling pathway is the most common transcription factor in macrophages to regulate the expression of a series of inflammatory genes and macrophage polarization. We have added to discuss that DCLK1 blockage reduces macrophage inflammation via inhibiting IKKβ/NF-κB to alleviate the progress of atherosclerotic plaques. iNOS is a marker of inflammatory phenotype of macrophages (Annu Rev Pathol. 2020, 15: 123-147). According to the comment, we also performed immunofluorescence staining of iNOS to investigate the inflammatory phenotype of macrophages. As shown in the *new Appendix Figure S3*, DCLK1 deletion reduced iNOS protein level in aortic roots of HFD-fed $ApoE^{-/-}$ mice, indicating decreased pro-inflammatory macrophages in atherosclerotic plaques. Other inflammatory factors including IL-1β and IL-18 are also important in the progression of atherosclerosis (Nat Rev Cardiol. 2019, 16(7): 389-406). We also examined *Il-1β* and *Il-18* mRNA levels and found that macrophage-specific DCLK1 deletion reduced HFD-increased mRNA levels of these genes in aortas of $ApoE^{-/-}$ mice (*new Appendix Figure S4*). These new data provide a more detailed characterization of the macrophage polarization type and the inflammatory milieu, validating that macrophage-specific DCLK1 deficiency significantly reduced inflammation in atherosclerotic plaques of HFD-fed $ApoE^{-/-}$ mice. We added these new data in the revised manuscript.

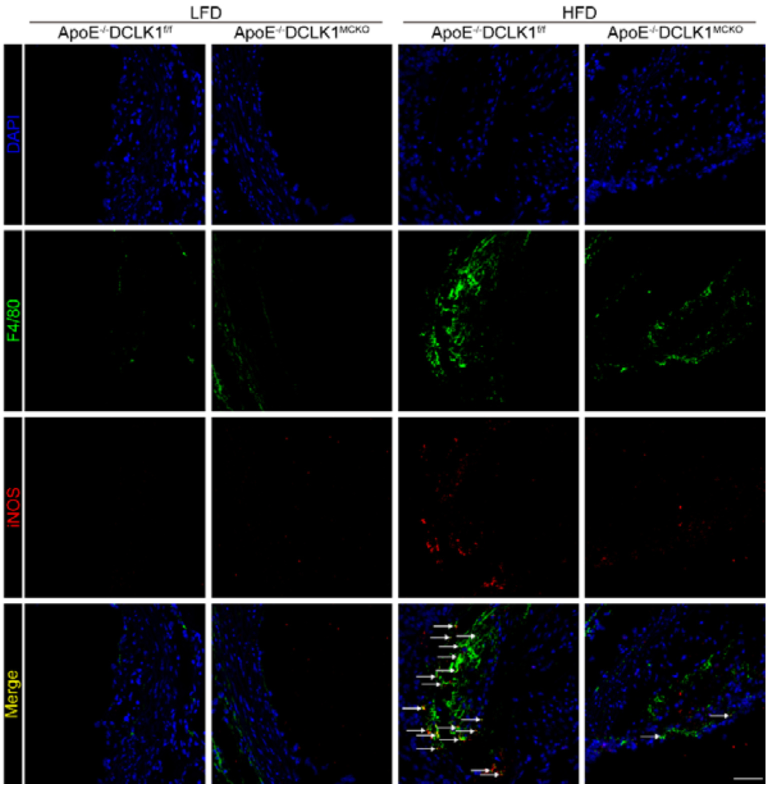

***New Appendix Figure S3*. DCLK1 deletion reduces pro-inflammatory macrophages in aortic roots of HFD-fed ApoE$^{-/-}$ mice.** Representative immunofluorescence staining of F4/80 (green) and iNOS (red) in aortic roots (scale bar=25 μm). Tissues were counterstained with DAPI (blue).

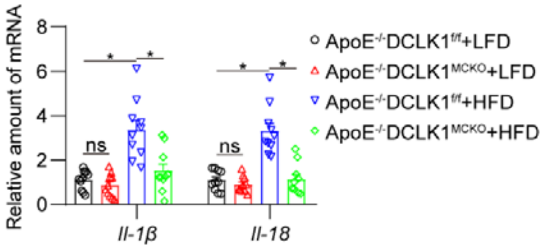

***New Appendix Figure S4*. mRNA levels of inflammatory cytokines *Il-1β* and *Il-18* in aortas.** The values of mRNA levels were normalized to *Rn18s* (n=10 biological replicates). Data were expressed as mean ± SEM; **P* < 0.05; ns, not significant, two-tailed unpaired Student's t-test.

Reviewer #1-3

The pictures in the manuscript have very low quality and should be replaced with a higher resolution. The authors should also provide pictures of en face stained aortas from all animals in the supplementary material. Also, the color code for the fluoresecent pictures is inappropriate (blue on black).

Response: Thanks for these suggestions. We replaced the pictures in the manuscript with a higher resolution in the revised figures. In addition, we provided all original pictures in a high resolution in 'Source Data' files. We have provided pictures of en face stained aortas from all groups in our atherosclerotic models (Figure 2A and Figure 7A). Many studies also showed

only one en face stained aorta from every groups and used blue to show DAPI staining on black background (*EBioMedicine* 2020, 53: 102706; *Nat Commun.* 2022, 13(1): 7500; *Nat Commun.* 2022, 13(1): 7721).

Reviewer #1-4

I respectfully point out that the language of the paper needs to be improved, as the authors have an unusual choice of verbs and there are missing words and grammatical errors.

Response: Thanks for the suggestion. We improved the language of the paper by language polishing and checked all grammatical errors in the manuscript.

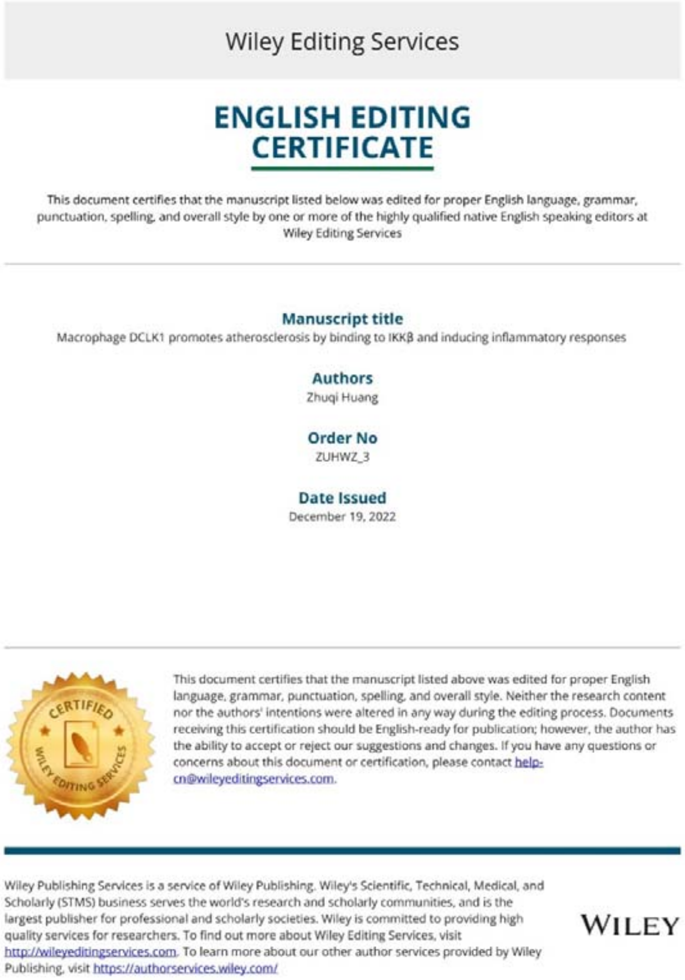
Wiley Editing Services

ENGLISH EDITING CERTIFICATE

This document certifies that the manuscript listed below was edited for proper English language, grammar, punctuation, spelling, and overall style by one or more of the highly qualified native English speaking editors at Wiley Editing Services

Manuscript title
Macrophage DCLK1 promotes atherosclerosis by binding to IKKβ and inducing inflammatory responses

Authors
Zhuqi Huang

Order No
ZUHWZ_3

Date Issued
December 19, 2022

This document certifies that the manuscript listed above was edited for proper English language, grammar, punctuation, spelling, and overall style. Neither the research content nor the authors' intentions were altered in any way during the editing process. Documents receiving this certification should be English-ready for publication; however, the author has the ability to accept or reject our suggestions and changes. If you have any questions or concerns about this document or certification, please contact help-cn@wileyeditingservices.com.

Wiley Publishing Services is a service of Wiley Publishing. Wiley's Scientific, Technical, Medical, and Scholarly (STMS) business serves the world's research and scholarly communities, and is the largest publisher for professional and scholarly societies. Wiley is committed to providing high quality services for researchers. To find out more about Wiley Editing Services, visit http://wileyeditingservices.com. To learn more about our other author services provided by Wiley Publishing, visit https://authorservices.wiley.com/

WILEY

Reviewer #1-5

The authors conclude in figure 2 that MCKO DCLK1 alleviates HFD-induced atherosclerotic development without affecting lipid metabolism. However, the authors do not did not provide any data on lipid metabolism besides blood lipid levels. Therefore, they should refer only to blood lipids.

Response: Thanks for the suggestion. We revised this sentence in the revised ‘Results’ section.

Reviewer #2:

Synopsis: The authors provide a comprehensive study, which looks technically sound - including all necessary controls. The role of DCLK1 in atherosclerosis is novel. So far it cannot be judged, whether it has a high medical impact (as human data are not yet shown). The mouse model is adequate - and professional. This study describes a proinflammatory role of the kinase DCLK1 in macrophages - and based on that a role in development of atherosclerosis in mice. It demonstrates that DCLK1 is upregulated in atherosclerotic plaques dependent on high fat diet (HFD) and oxLDL and that a macrophage-specific deletion of this gene attenuates atherosclerosis in HFD-fed ApoE knockout mice. Data shown by the authors indicate that macrophage-deletion of DCLK1 reduces infiltration of monocytes and neutrophils into the plaques. RNA-sequencing of macrophages derived from DCLK1-KO and control mice and treated with OxLDL revealed a significant downregulation of the NF-κB pathway (as shown by GSEA, as well as Western blot analysis of p65 phosphorylation and IKB degradation). Immunoprecipitation of flag-tagged DCLK1 overexpressed in 293 cells, followed by MS-based identification of bound proteins revealed IKKβ as binding partner. The authors then show that DCLK1 can phosphorylate the serine residues of the IKKβ activation loop. Pharmacological inhibition of DCLK1 resulted in a reduction of IKKβ activation, p65 phosphorylation and IKB degradation in vitro and also attenuated atherosclerosis in vivo. Altogether the manuscript presents a comprehensive study with all the required controls. There are just some points left to be clarified.

Response: Thank you for your positive comments. We substantially revised the manuscript according to your suggestions point-by-point.

Reviewer #2-1

Fig. 1D: The authors should specify in more detail, how quantitative PCR was done (which method was used to calculate fold induction, has the PCR efficiency been considered?)

Response: Thanks for the suggestion. We added how real-time quantitative PCR was done in the revised 'Materials and Methods' section.

Reviewer #2-2

Fig. 3G: percentages of the various leukocyte subtypes should be shown in the scattergrams.

Response: Thanks. Neutrophil and monocyte in plasma were measured by an automated blood cell analyzer (XN-1000, Sysmex Corporation, Kobe, Japan). The images in Figure 3G is the scatter diagrams of various leukocyte subtypes. We also showed statistical graph with statistical dots of percentages of neutrophil and monocyte in plasma (Figure 3H and Figure 3I).

Reviewer #2-3

Fig. 4H: The p-value color coding should be changed to obtain a better visualization (e.g., by setting 0.0 as red and 0.05 as blue (instead of 1.00). Alternatively, the bars might be labeled with the numerical p-value.

Response: Thanks for the suggestion. We labeled the bars with the numerical p-value in the *Updated Figure 4H*.

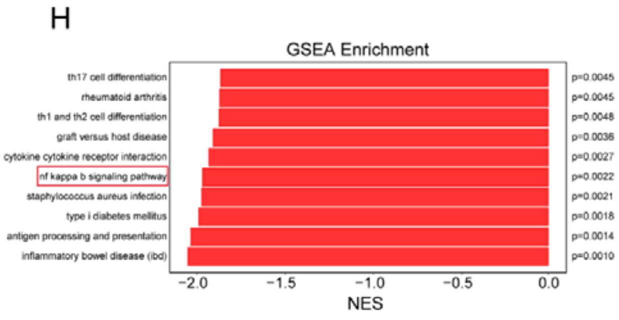

Updated Figure 4H

Reviewer #2-4

Fig. 4P: Nuclear translocation is not obvious from the images. Images should be shown without the DAPI channel for better assessment of p65 nuclear translocation. The authors should specify in more detail how nuclear translocation was quantified. Ideally, nuclear/cytosol ratios should be determined for the cells and statistics calculated.

Response: Thanks for this comment. Accordingly, we carefully checked the staining images for p65 nuclear translocation in Figure 4P and 7J. We fully agree with the reviewer that the current immunofluorescence staining images could not reflect the p65 nuclear translocation very well in in aortic root tissues, since the DAPI staining is not specific in the nuclear of infiltrated macrophages. Therefore, we have to find a new characterization to indicate NF-kB activity in aortic root tissues. NF-kB p65 activity contains two steps: p65 phosphorylation and p65 nuclear translocation. Thus, we performed the immunofluorescence staining for phosphorylated p65 (p-p65) to examine the profile of NF-kB p65 activity in the aortic root tissues. The p-p65 staining data are showed in the new Figure 4P-Q and Figure 7J. These new data indicate that HFD increased p-p65 level in aortic root tissues, while either DCLK1 deletion (new Figure 4P-Q) or inhibitor (new Figure 7J) significantly suppressed HFD-increased p65 phosphorylation in atherosclerotic mice. We consider that replacing the original p65 nuclear translocation staining images with these new data on p-p65 immunofluorescence staining is able to better indicate the profile of NF-kB p65 activity in the aortic root tissues of mice.

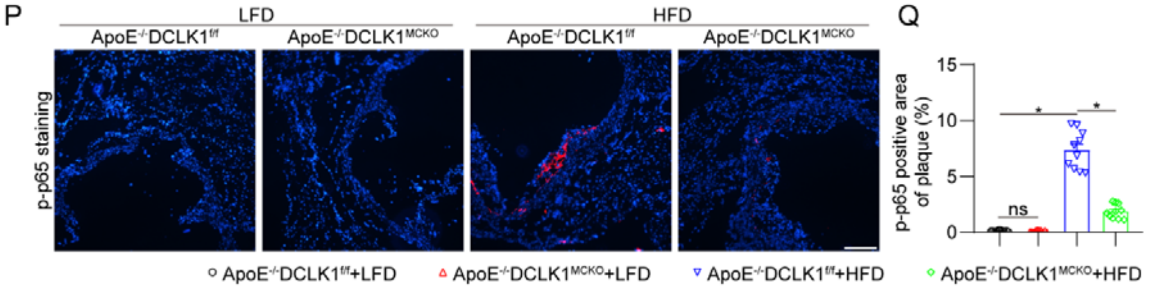

New Figure 4P-Q. Representative immunofluorescence staining images (P) and quantification (Q) of p-p65 (red) in aortic roots. Tissues were counterstained with DAPI (blue). Scale bar=100 μm, n=10 biological replicates. p-p65 area was quantified by the proportion of p-p65 positive area to plaque area. Data were shown as mean ± SEM; *, $P < 0.05$; ns, not significant, two-tailed unpaired Student's t-test.

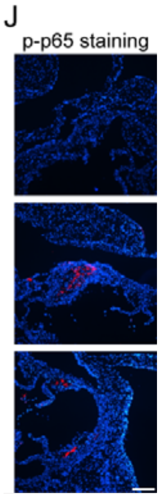

New Figure 7J. Representative immunofluorescence staining images of p-p65 (red) in aortic roots. Tissues were counterstained with DAPI (blue). Scale bar=100 μm.

Reviewer #3:

Synopsis: This is overall an impressive manuscript with novel and clinically potentially important data. I have listed some concerns/suggestions which the authors should be able to address. Atherosclerosis is a chronic inflammatory disease that results in the formation of immune cell-rich plaques in the arterial wall, leading to high morbidity and mortality in the world. Inflammation is a process that plays an important role in the initiation and progression of atherosclerosis, involving multiple cell types, such as macrophages, T cells and other cells of the immune response. The manuscript by Huang et al. focuses on the role of doublecortin-like serine/threonine kinase 1 (DCLK1) in inflammatory diseases, such as atherosclerosis. Recent studies have indicated a role of the microtubule-associated protein DCLK1 as a tumor stem cell marker as well as in neurogenesis and neuronal migration. So far, the function of DCLK1 in atherosclerosis has not been defined yet. To determine if DCLK1 plays a role in this cardiovascular disease, Huang et al. investigated and reported up-regulated DCLK1 levels in macrophages of ApoE-/- mice fed a HFD and suggest that macrophage-specific DCLK1 deletion attenuates atherosclerosis by reducing inflammation in mice. Implemented RNA sequencing analysis indicated that DCLK1 could mediate oxLDL-induced inflammation via NF-κB signaling pathway in primary macrophages. Co-immunoprecipitation followed by LC-MS/MS analysis suggests IKKβ as a binding protein of DCLK1. The three claimed key findings of the present study are as follows: (1) Macrophage-specific deletion or pharmacological inhibition of DCLK1 attenuates atherosclerotic progression; (2) macrophage-specific deletion or pharmacological inhibition of DCLK1 alleviates chronic inflammation by inhibiting NF-κB activation; (3) DCLK1 directly binds to IKKβ and phosphorylates IKKβ at S177/181. Overall, the manuscript is well written, the results are well presented and discussed appropriately. The suggestions of DCLK1 as a new IKKβ regulator in inflammation and a potential therapeutic target for inflammatory atherosclerosis are very interesting and the novelty is very high since a correlation to atherosclerosis has not been shown in the literature yet. However, the study comprises some limitations. The following concerns and suggestions might help the authors to improve their manuscript.

Response: Thank you for your positive comments. We substantially revised the manuscript according to your suggestions point-by-point.

Reviewer #3-1

Introduction: In the introduction the authors state to have been engaged in finding new inflammatory mechanisms and targets for "many years" while citing two recent publications from 2020 and 2022. I would argue that this sentence should not be included in the introduction as it does not help to introduce the topic or the study.

Response: Thanks for the suggestion. We deleted this sentence in the revised 'Introduction' section.

Reviewer #3-2

Figure 1B shows a very tightly framed band for Western blot results and is cut off very close to the bands shown. We suggest that the authors provide entire Western blots.

Response: Thanks for the suggestion. We replaced the western blot band with more appropriate one in the updated Figure 1B. We also provided the uncropped western blots in the "Source Data" files.

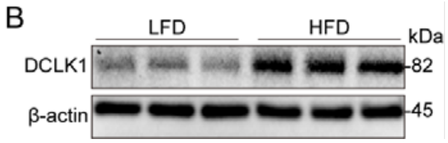

Updated Figure 1B

Reviewer #3-3

Figure 2E and 7E: Most examples of plaque in the sinus shown do not present intact sinus valves and hence cannot be used for comparisons. Here better sections and images would be helpful.

Response: Thanks for this suggestion. We replaced some images with more representative images presenting intact sinus valves in the *Updated Figure 2E and 7E*. We also quantified plaque area by the proportion of plaque area to aortic root area. We added these images in the revised manuscript.

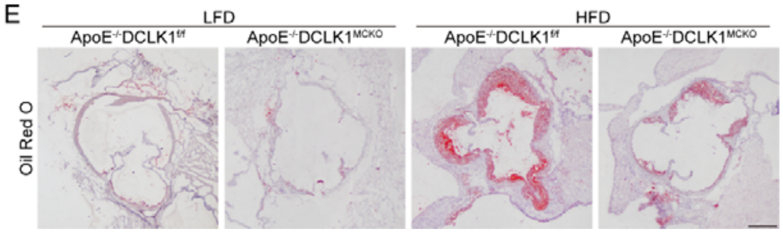

Updated Figure 2E

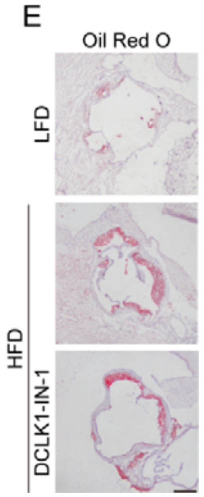

Updated Figure 7E

Reviewer #3-4

Figure 2G, Figure 3C-E, Figure 7F-J: Shown extracts of the tissue samples are too small to validate the staining. The staining of, e.g., Ly6G, and Ly6C, shows a lot of background information. The authors need to demonstrate the specificity of the antibodies used with the chosen staining methods and the respective negative (e.g., isotype controls)?

Response: Thanks for the suggestion. We provided all original pictures with big size and high resolution in 'Source Data' files. We demonstrated the specificity of the antibodies against Ly6G (#sc-53515) and Ly6C (#sc-271811) (*Figure R1*). These antibodies have high reputation and good quality, and many previous studies utilized these antibodies for immunostaining examinations (*Cardiovasc Res.* 2022, 118(9): 2165-2178; *EBioMedicine*. 2022, 84: 104275; *Acta Pharmacol Sin.* 2022, Jun 16; *J Neuroinflammation*. 2019, 16(1): 198). Antibodies against normal mouse IgG (#sc-2025), normal rat IgG (#sc-2026), Ly6G (#sc-53515), and

Ly6C (#sc-271811) were purchased from Santa Cruz Biotechnology (Santa Cruz, CA, USA).

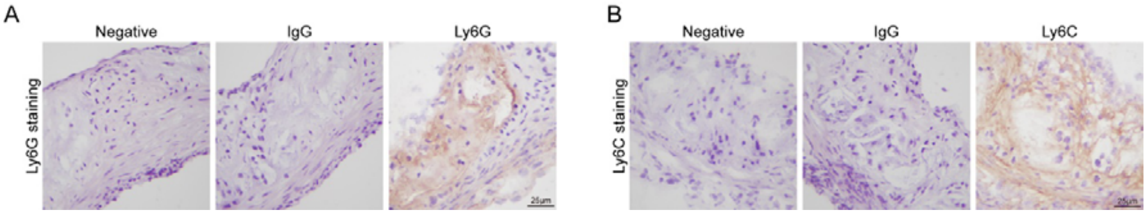

Figure R1 (only for review). Representative immunohistochemistry staining images of Ly6G (A) and Ly6C (B) in aortic roots (scale bar=25 μm).

Reviewer #3-5

Figure 4P: The representative immunofluorescence stainings of p65 do not show clear differences. How was the quantification in Figure 4Q implemented? Can the authors provide proof of the specificity of the antibodies used in the respective staining procedures (negative controls, isotype controls, positive tissues). The same concern and consequent request apply to Figure 3A, and Figure 7J)?

Response: Thanks for these suggestions.

(1) The question on p65 immunofluorescence staining is also raised by Reviewer #2. Please see our response to Reviewer #2-4.

(2) We quantified F4/80 area by the proportion of F4/80-positive area to aortic root area. We added the methodological information in the revised Figure Legend.

(3) We demonstrated the specificity of these antibodies against p-p65 (#3033) and F4/80 (#ab6640) (*Figure R2*). These antibodies have high reputation and good quality, and many previous studies utilized these antibodies for immunofluorescence staining examinations (Nat Med. 2018, 24(6): 770-781; Nature 2019, 574 (7778): 428-431; Nat Commun. 2021, 12(1): 1394; Sci Adv. 2021, 7(9): eabf4398). The antibody against normal rat IgG (#sc-2026) was purchased from Santa Cruz Biotechnology (Santa Cruz, CA, USA) and the antibody against normal rabbit IgG (#30000-0-AP) was purchased from Proteintech (Wuhan, China).

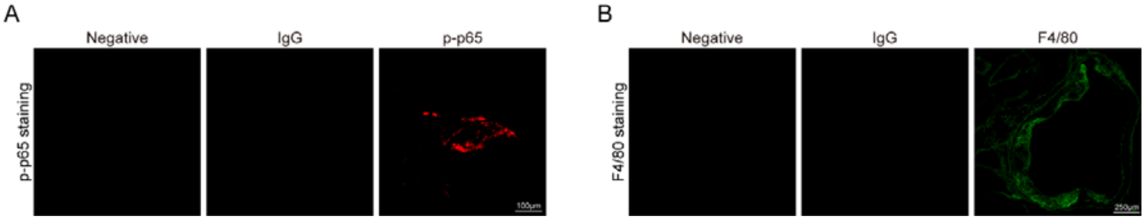

Figure R2 (only for review). (A) Representative immunofluorescence staining images of p-p65 (A) and F4/80 (B) in aortic roots. Scale bar=100 μm and 250 μm, respectively.

Reviewer #3-6

Figure 7C: The representative examples shown do not appear to be less than 5% as stated in the quantification in Figure 7D. In addition, the pictures represent different illumination between 3 examples given and do not provide a qualitative source for quantification.

Response: Thanks. We carefully checked the original images in Figure 7C and acknowledged that, in the image of LFD group, there were some connective tissue and adipose tissue in the perivascular area, which may confuse the readers. In addition, we are sorry for the brightness

difference in this picture because we captured it using a mobile camera. Therefore, we replaced the image of LFD group with a more representative image, which was taken from the same illumination as the pictures of other two groups (HFD group and HFD+DCLK1-IN-1 group) in the *Updated Figure 7C*. We also re-performed the statistics (*Updated Figure 7D*). The plaque area was quantified by the proportion of plaque area to aortic arch area. We updated these results in the revised manuscript.

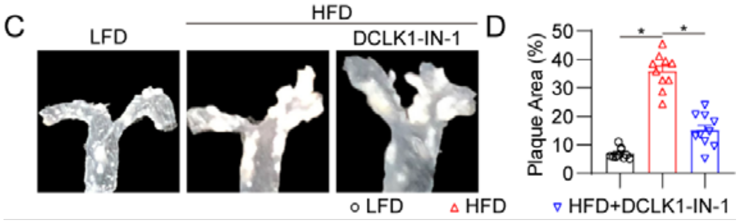

Updated Figure 7C-D

Reviewer #3-7

Figure 7A: The authors should provide the original pictures since the background of some of the pictures, e.g., Figure 7A, seem "really black". This would be helpful to convince the reviewer and the readers that the post-processing has not changed the quantification.

Response: Thanks for the suggestion. We provided all original pictures in 'Source Data' files.

Reviewer #3-8

Discussion: A schematic representation/ graphic abstract of the main findings is mentioned in the first paragraph of the Discussion. This graphic overview could not be found by this reviewer.

Response: Thanks. The graphic abstract has been included in the 'Synopsis' file.

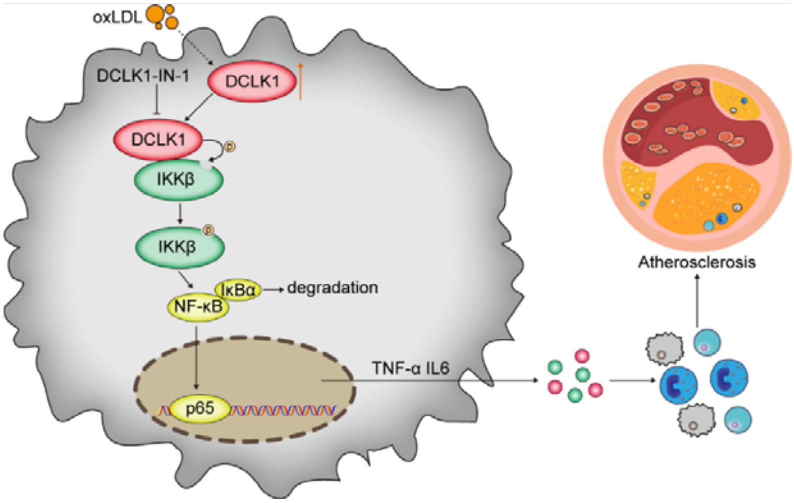

The graphic abstract in the 'Synopsis' file

1st Revision - Editorial Decision 21st Feb 2023

Dear Prof. Liang,

Thank you for the submission of your revised manuscript to EMBO Molecular Medicine. We appreciate providing original images used for quantifications from all 10 mice. I am pleased to inform you that we will be able to accept your manuscript pending the following final amendments:

1) Please address the referee #1 concern about mouse data reproducibility, particularly regarding power calculation.
2) In the main manuscript file, please do the following:

- Correct/answer the track changes suggested by our data editors by working from the attached document.
- Remove abbreviation list.
- In M&M, provide the antibody dilutions that were used for each antibody.
- In M&M, the statistical paragraph should reflect all information that you have filled in the Authors Checklist, especially regarding randomization, blinding, replication.
- Author contributions: Please remove it from the manuscript and specify author contributions in our submission system. CRediT has replaced the traditional author contributions section because it offers a systematic machine-readable author contributions format that allows for more effective research assessment. You are encouraged to use the free text boxes beneath each contributing author's name to add specific details on the author's contribution. More information is available in our guide to authors:
https://www.embopress.org/page/journal/17574684/authorguide#authorshipguidelines
- Add citations for all public datasets used in the study (GSE94044 and GSE137581) in the text and reference list.
- Data availability statement: Remove information about appendix. Please be aware that all deposited datasets should be made freely available upon acceptance, without restriction. PXD039083 is currently not accessible.
Please check "Author Guidelines" for more information.
https://www.embopress.org/page/journal/17574684/authorguide#availabilityofpublishedmaterial

3) The Paper Explained: Please add it to the main manuscript file.
4) Synopsis: Please check your synopsis text and image before submission with your revised manuscript. Please be aware that in the proof stage minor corrections only are allowed (e.g., typos).
5) For more information: This space should be used to list relevant web links for further consultation by our readers. Could you identify some relevant ones and provide such information as well? Some examples are patient associations, relevant databases, OMIM/proteins/genes links, author's websites, etc...
6) As part of the EMBO Publications transparent editorial process initiative (see our Editorial at http://embomolmed.embopress.org/content/2/9/329), EMBO Molecular Medicine will publish online a Review Process File (RPF) to accompany accepted manuscripts. This file will be published in conjunction with your paper and will include the anonymous referee reports, your point-by-point response and all pertinent correspondence relating to the manuscript. Let us know whether you agree with the publication of the RPF and as here, if you want to remove or not any figures from it prior to publication. Please note that the Authors checklist will be published at the end of the RPF.
7) Please provide a point-by-point letter INCLUDING my comments as well as the reviewer's reports and your detailed responses (as Word file).

I look forward to reading a new revised version of your manuscript as soon as possible.

Yours sincerely,

Zeljko Durdevic
Editor
EMBO Molecular Medicine

***** Reviewer's comments *****

Referee #1 (Remarks for Author):

The explanations on mouse experiment are questionable and the data surprisingly tight for such low mouse numbers, especially as so many alleles are being interbred, which usually creates even more biological variation in atherosclerosis. No power calculation was performed so it remains magic that the desired outcome was observed. Essentially, we are looking at 1-2 round of litters. This raises doubts about the mouse data reproducibility.

Referee #3 (Remarks for Author):

The authors have addressed my previous comments adequately.

Reviewer #1-1

The explanations on mouse experiment are questionable and the data surprisingly tight for such low mouse numbers, especially as so many alleles are being interbred, which usually creates even more biological variation in atherosclerosis. No power calculation was performed so it remains magic that the desired outcome was observed. Essentially, we are looking at 1-2 round of litters. This raises doubts about the mouse data reproducibility.

Response: Thanks for these suggestions.

(1) We have increased the number of mice per group to 10 in all animal experiments. We also uploaded all original images (histological and western blots) used for quantifications in the animal experiments in the “Source data” file. Although the mice were with triple gene-editing ($ApoE^{-/-}DCLK1^{f/f}$-$Lyz2^{Cre}$ and $ApoE^{-/-}DCLK1^{f/f}$), the method of $ApoE^{-/-}$ mice crossed with other gene-knockout mice were generally accepted model to investigate atherosclerosis (*Circulation*. 2022 Mar;145(9):675-687; *Circ Res*. 2020 Aug 28;127(6):811-823). In addition, we have already showed the genotyping validation of $ApoE^{-/-}DCLK1^{f/f}$ and $ApoE^{-/-}DCLK1^{MCKO}$ mice and the DCLK1 expression examination in Appendix Figure S1.

(2) Sample sizes were defined by a priori power calculation with G-Power 3.1.9 software (http://www.gpower.hhu.de/), considering a statistical power of 80% and α=0.05. We updated this information in the ‘Materials and Methods’ section.

Reviewer #3-1

The authors have addressed my previous comments adequately.

Response: Thank you very much.

2nd Revision - Editorial Decision 23rd Feb 2023

We are pleased to inform you that your manuscript is accepted for publication and is now being sent to our publisher to be included in the next available issue of EMBO Molecular Medicine.

EMBO Press Author Checklist

Corresponding Author Name: Guang Liang
Journal Submitted to: EMBO Molecular Medicine
Manuscript Number: EMM-2022-17198

USEFUL LINKS FOR COMPLETING THIS FORM
The EMBO Journal - Author Guidelines
EMBO Reports - Author Guidelines
Molecular Systems Biology - Author Guidelines
EMBO Molecular Medicine - Author Guidelines

Reporting Checklist for Life Science Articles (updated January 2022)
This checklist is adapted from Materials Design Analysis Reporting (MDAR) Checklist for Authors. MDAR establishes a minimum set of requirements in transparent reporting in the life sciences (see Statement of Task: 10.31222/osf.io/9sm4x). Please follow the journal's guidelines in preparing your manuscript.
Please note that a copy of this checklist will be published alongside your article.

Abridged guidelines for figures
1. Data
The data shown in figures should satisfy the following conditions:
- the data were obtained and processed according to the field's best practice and are presented to reflect the results of the experiments in an accurate and unbiased manner.
- ideally, figure panels should include only measurements that are directly comparable to each other and obtained with the same assay.
- plots include clearly labeled error bars for independent experiments and sample sizes. Unless justified, error bars should not be shown for technical replicates.
- if n<5, the individual data points from each experiment should be plotted. Any statistical test employed should be justified.
- Source Data should be included to report the data underlying figures according to the guidelines set out in the authorship guidelines on Data Presentation.

2. Captions
Each figure caption should contain the following information, for each panel where they are relevant:
- a specification of the experimental system investigated (eg cell line, species name).
- the assay(s) and method(s) used to carry out the reported observations and measurements.
- an explicit mention of the biological and chemical entity(ies) that are being measured.
- an explicit mention of the biological and chemical entity(ies) that are altered/varied/perturbed in a controlled manner.
- the exact sample size (n) for each experimental group/condition, given as a number, not a range;
- a description of the sample collection allowing the reader to understand whether the samples represent technical or biological replicates (including how many animals, litters, cultures, etc.).
- a statement of how many times the experiment shown was independently replicated in the laboratory.
- definitions of statistical methods and measures:
  - common tests, such as t-test (please specify whether paired vs. unpaired), simple χ2 tests, Wilcoxon and Mann-Whitney tests, can be unambiguously identified by name only, but more complex techniques should be described in the methods section;
  - are tests one-sided or two-sided?
  - are there adjustments for multiple comparisons?
  - exact statistical test results, e.g., P values = x but not P values < x;
  - definition of 'center values' as median or average;
  - definition of error bars as s.d. or s.e.m.

Please complete ALL of the questions below.
Select "Not Applicable" only when the requested information is not relevant for your study.

Materials

Newly Created Materials	Information included in the manuscript?	In which section is the information available? (Reagents and Tools Table, Materials and Methods, Figures, Data Availability Section)
New materials and reagents need to be available; do any restrictions apply?	Not Applicable	

Antibodies	Information included in the manuscript?	In which section is the information available? (Reagents and Tools Table, Materials and Methods, Figures, Data Availability Section)
For **antibodies** provide the following information:<br>- Commercial antibodies: RRID (if possible) or supplier name, catalogue number and or/clone number<br>- Non-commercial: RRID or citation	Yes	Materials and Methods

DNA and RNA sequences	Information included in the manuscript?	In which section is the information available? (Reagents and Tools Table, Materials and Methods, Figures, Data Availability Section)
**Short novel DNA or RNA including primers, probes**: provide the sequences.	Yes	Materials and Methods

Cell materials	Information included in the manuscript?	In which section is the information available? (Reagents and Tools Table, Materials and Methods, Figures, Data Availability Section)
**Cell lines:** Provide species information, strain. Provide accession number in repository **OR** supplier name, catalog number, clone number, and/**OR** RRID.	Yes	Materials and Methods
**Primary cultures:** Provide species, strain, sex of origin, genetic modification status.	Yes	Materials and Methods
Report if the cell lines were recently **authenticated** (e.g., by STR profiling) and tested for mycoplasma contamination.	Not Applicable	

Experimental animals	Information included in the manuscript?	In which section is the information available? (Reagents and Tools Table, Materials and Methods, Figures, Data Availability Section)
**Laboratory animals or Model organisms:** Provide species, strain, sex, age, genetic modification status. Provide accession number in repository **OR** supplier name, catalog number, clone number, **OR** RRID.	Yes	Materials and Methods
**Animal observed in or captured from the field:** Provide species, sex, and age where possible.	Not Applicable	
Please detail **housing and husbandry conditions**.	Yes	Materials and Methods

Plants and microbes	Information included in the manuscript?	In which section is the information available? (Reagents and Tools Table, Materials and Methods, Figures, Data Availability Section)
**Plants:** provide species and strain, ecotype and cultivar where relevant, unique accession number if available, and source (including location for collected wild specimens).	Not Applicable	
**Microbes:** provide species and strain, unique accession number if available, and source.	Not Applicable	

Human research participants	Information included in the manuscript?	In which section is the information available? (Reagents and Tools Table, Materials and Methods, Figures, Data Availability Section)
If collected and within the bounds of privacy constraints report on age, sex and gender or ethnicity for all study participants.	Not Applicable	

Core facilities	Information included in the manuscript?	In which section is the information available? (Reagents and Tools Table, Materials and Methods, Figures, Data Availability Section)
If your work benefited from core facilities, was their service mentioned in the acknowledgments section?	Not Applicable	

Design

Study protocol	Information included in the manuscript?	In which section is the information available? (Reagents and Tools Table, Materials and Methods, Figures, Data Availability Section)
If study protocol has been **pre-registered, provide DOI in the manuscript**. For clinical trials, provide the trial registration number **OR** cite DOI.	Not Applicable	
Report the **clinical trial registration number** (at ClinicalTrials.gov or equivalent), where applicable.	Not Applicable	

Laboratory protocol	Information included in the manuscript?	In which section is the information available? (Reagents and Tools Table, Materials and Methods, Figures, Data Availability Section)
Provide DOI OR other citation details if **external detailed step-by-step protocols** are available.	Not Applicable	

Experimental study design and statistics	Information included in the manuscript?	In which section is the information available? (Reagents and Tools Table, Materials and Methods, Figures, Data Availability Section)
Include a statement about **sample size** estimate even if no statistical methods were used.	Yes	Figure Legends
Were any steps taken to minimize the effects of subjective bias when allocating animals/samples to treatment (e.g. **randomization procedure**)? If yes, have they been described?	Yes	Materials and Methods
Include a statement about **blinding** even if no blinding was done.	Yes	Materials and Methods
Describe **inclusion/exclusion criteria** if samples or animals were excluded from the analysis. Were the criteria pre-established?<br><br>If sample or data points were omitted from analysis, report if this was due to attrition or intentional exclusion and provide justification.	Not Applicable	
For every figure, are **statistical tests** justified as appropriate? Do the data meet the assumptions of the tests (e.g., normal distribution)? Describe any methods used to assess it. Is there an estimate of variation within each group of data? Is the variance similar between the groups that are being statistically compared?	Yes	Figure Legends

Sample definition and in-laboratory replication	Information included in the manuscript?	In which section is the information available? (Reagents and Tools Table, Materials and Methods, Figures, Data Availability Section)
In the figure legends: state number of times the experiment was **replicated** in laboratory.	Yes	Figure Legends
In the figure legends: define whether data describe **technical or biological replicates**.	Yes	Figure Legends

Ethics

Ethics	Information included in the manuscript?	In which section is the information available? (Reagents and Tools Table, Materials and Methods, Figures, Data Availability Section)
Studies involving **human participants**: State details of **authority granting ethics approval** (IRB or equivalent committee(s), provide reference number for approval.	Not Applicable	
Studies involving **human participants**: Include a statement confirming that **informed consent** was obtained from all subjects and that the experiments conformed to the principles set out in the WMA Declaration of Helsinki and the Department of Health and Human Services Belmont Report.	Not Applicable	
Studies involving **human participants:** For publication of **patient photos**, include a statement confirming that consent to publish was obtained.	Not Applicable	
Studies involving experimental **animals**: State details of **authority granting ethics approval** (IRB or equivalent committee(s), provide reference number for approval. Include a statement of compliance with ethical regulations.	Yes	Materials and Methods
Studies involving **specimen and field samples:** State if relevant **permits** obtained, provide details of authority approving study; if none were required, explain why.	Not Applicable	

Dual Use Research of Concern (DURC)	Information included in the manuscript?	In which section is the information available? (Reagents and Tools Table, Materials and Methods, Figures, Data Availability Section)
Could your study fall under dual use research restrictions? Please check biosecurity documents and list of **select agents and toxins** (CDC): https://www.selectagents.gov/sat/list.htm	Not Applicable	
If you used a select agent, is the security level of the lab appropriate and reported in the manuscript?	Not Applicable	
If a study is subject to dual use research of concern regulations, is the name of the **authority granting approval and reference number** for the regulatory approval provided in the manuscript?	Not Applicable	

Reporting

The MDAR framework recommends adoption of discipline-specific guidelines, established and endorsed through community initiatives. Journals have their own policy about requiring specific guidelines and recommendations to complement MDAR.

Adherence to community standards	Information included in the manuscript?	In which section is the information available? (Reagents and Tools Table, Materials and Methods, Figures, Data Availability Section)
State if relevant guidelines or checklists (e.g., **ICMJE, MIBBI, ARRIVE, PRISMA**) have been followed or provided.	Not Applicable	
For **tumor marker prognostic studies,** we recommend that you follow the **REMARK** reporting guidelines (see link list at top right). See author guidelines, under 'Reporting Guidelines'. Please confirm you have followed these guidelines.	Not Applicable	
For **phase II and III randomized controlled trials**, please refer to the **CONSORT** flow diagram (see link list at top right) and submit the CONSORT checklist (see link list at top right) with your submission. See author guidelines, under 'Reporting Guidelines'. Please confirm you have submitted this list.	Not Applicable	

Data Availability

Data availability	Information included in the manuscript?	In which section is the information available? (Reagents and Tools Table, Materials and Methods, Figures, Data Availability Section)
Have **primary datasets** been deposited according to the journal's guidelines (see 'Data Deposition' section) and the respective accession numbers provided in the Data Availability Section?	Yes	Data Availability
Were **human clinical and genomic datasets** deposited in a public access-controlled repository in accordance to ethical obligations to the patients and to the applicable consent agreement?	Not Applicable	
Are **computational models** that are central and integral to a study available without restrictions in a machine-readable form? Were the relevant accession numbers or links provided?	Not Applicable	
If publicly available data were reused, provide the respective **data citations in the reference list**.	Yes	Reference
